# The application of artificial intelligence techniques in predicting game outcomes of professional basketball league: A systematic review

**DOI:** 10.1371/journal.pone.0326326

**Published:** 2025-06-26

**Authors:** Chuxuan Li, Hao Zhang, Yuan Zhang, Jing Shen, Ruopeng An

**Affiliations:** 1 Department of Physical Education, China University of Geosciences, Beijing, China; 2 Constance and Martin Silver Center on Data Science and Social Equity, New York University Silver School of Social Work, New York, United States of America; Sapienza University of Rome, ITALY

## Abstract

**Background:**

Predicting basketball game outcomes is a critical area in sports science and data analysis, providing concrete benefits for optimizing coaching strategies, improving team management, and informing betting decisions.

**Objective:**

This methodological review systematically evaluates the effectiveness of specific artificial intelligence technologies in predicting professional basketball game outcomes over the past five years from 2019 to 2024, providing detailed insights into current methodologies and identifying emerging trends and challenges in this domain.

**Methods:**

Following PRISMA-SCR guidelines, a comprehensive keyword search was conducted across four electronic bibliographic databases: PubMed, Web of Science, Scopus, and EBSCO. Studies were included if they utilized artificial intelligence techniques, focused on professional leagues, and aimed to predict game outcomes.

**Results:**

This review incorporated 34 studies that met the predefined eligibility criteria, examining various artificial intelligence techniques used to predict professional basketball game outcomes over the past five years. The findings reveal that artificial intelligence models, particularly the multilayer perceptron neural network, achieved a high prediction accuracy of 98.90%. The random forest model, based on four factors, reached an accuracy of 93.81%, while the voting regression ensemble model achieved 93.3%. The studies underscore the importance of effective data processing and feature selection in enhancing model performance. Additionally, dynamic prediction models that adapt to real-time changes in the game were shown to be particularly useful for tactical decisions and betting strategies.

**Conclusions:**

Artificial intelligence significantly improves the accuracy of predicting outcomes in professional basketball games. Future research should include diverse basketball leagues and employ more advanced validation techniques to enhance model robustness and applicability. Integrating real-time data and exploring transfer learning will likely improve prediction accuracy and decision-making support.

## Introduction

Basketball is a widely followed sport globally and the National Basketball Association (NBA) is the premier professional basketball league in the United States [1]. In these competitive environments, accurate predictions of game outcomes can provide valuable insights for coaching staff, helping them to better understand opponents and develop effective strategies [2]. Additionally, these predictions can inform team management decisions regarding player acquisitions, lineup adjustments, and other strategic areas [3]. For bettors and investors, reliable prediction models are important tools for making informed decisions [4]. As a result, the technology for forecasting outcomes in professional basketball games has become a critical area of research within sports analytics.

As the volume and complexity of game data increased, the limitations of traditional methods in predicting match outcomes became increasingly evident. Traditional sports science relies heavily on the expertise of coaches, team leaders, and analysts [5]. Initially, data collection for competitions was primarily manual [6]. However, as data volumes rapidly increased, traditional methods proved insufficient data processing capacity, often resulting in incomplete data and low prediction accuracy [7]. Over the past few decades, advancements in computer technology have driven scholars to explore more efficient methods to predict match outcomes. In 1997, the PC-based Advanced Scout system was developed, marking the entry of NBA data analytics into data mining and machine learning (ML) [8]. As artificial intelligence (AI) technology advances, AI has become a popular research focus in the field of sports outcome prediction, encompassing a variety of sports, including basketball [9–13].

Among various sports events, the prediction of basketball game outcomes has garnered particular attention due to its complexity and high level of competitiveness. Existing research has explored the application of AI in various areas, including performance prediction [14–16], injury risk assessment [17–19], game outcome prediction [20–24], player performance evaluation [25–27], team performance evaluation [28–30], tactical lineup optimization [31–33], action recognition [34–36], and tactical decision-making [37–39]. Among these, researchers have made significant progress in predicting basketball game outcomes using various AI models [1–3,9,20,40,41]. For example, some studies have developed hybrid ensemble learning frameworks that effectively addressed issues such as feature redundancy, sample noise, and dataset imbalance by integrating bagging strategies and random subspace algorithms, achieving an accuracy rate of 84.00% [3]. Similarly, an intelligent framework based on ML and feature selection was proposed to predict NBA game outcomes [9]. Using naive bayes (NB), artificial neural networks (ANN), and decision tree (DT) models, the study identified defensive rebounding as the most significant factor influencing game outcomes [9]. Additionally, seven different ML models were utilized to predict NBA game outcomes, with the k-nearest neighbors (KNN) algorithm yielded the best overall prediction results, with an average accuracy of 60.01% [41].

Despite considerable research on AI applications for predicting basketball match outcomes, the effectiveness of these methods varies significantly, and comprehensive review studies remain scarce. The few existing reviews suffer from several limitations, including a lack of focus on game outcome prediction, the absence of a systematic literature review methodology, the inclusion of a limited number of studies, and the frequent omission of recent literature, particularly literature that reflects the rapid advancements in AI technology in recent two years [42,43]. Additionally, the lack of comprehensive evaluation of different AI methods’ effectiveness across various leagues has hindered the practical determination of the most suitable AI techniques for different scenarios. Notably, these reviews often overlook the uniqueness of professional basketball, which features higher competition levels, more complex tactical systems, and closer teamwork.

This review aims to addressed the gaps identified by providing a comprehensive and systematic evaluation of AI technologies in predicting professional basketball match outcomes, specifically focusing on developments from the past five years. Over this period, AI technology has made significant advancements in algorithms, models, and applications, making it an essential timeframe for capturing the current state and emerging trends in the field. By focusing on this period, we aim to better reflect the latest technological progress and application trends. Specifically, this study will make contributions in the following three areas: first, we will summarize the specific application areas of existing AI technologies in predicting basketball match outcomes, providing a comprehensive and accessible introduction to core AI methods to facilitate their widespread dissemination and application in both academic and practical sports analytics. Second, we will conduct an in-depth examination of the application of AI technologies in data collection and processing, feature selection and extraction, identifying the key factors that influence prediction accuracy. By analyzing how these factors impact AI model performance across different scenarios, we aim to propose optimization strategies for improving prediction accuracy. Third, we will conduct a thorough evaluation of the accuracy and applicability of different AI methods for predicting professional basketball match outcomes. By comparing various models in detail, we will identify the strengths and limitations of each model across different scenarios, providing a scientific basis for optimizing and selecting the most appropriate AI models.

The remainder of this review is structured as follows: Section 2 (Methods) describes the methodology used in this review, including study selection criteria, search strategy, data extraction and synthesis, and study quality assessment. Section 3 (Results) presents the findings from the reviewed studies, including identified AI methodologies, predictive performance, and key influencing factors. Section 4 (Discussion) provides a comparative analysis of AI techniques, the impact of feature selection and data preprocessing, model validation methods, and a discussion of static versus dynamic predictions. This section also compares our findings with previous studies and highlights limitations and future research directions. Finally, Section 5 (Conclusion) summarizes the key findings of this review, emphasizing the effectiveness of AI in predicting basketball game outcomes and the potential for future advancements in this field.

## Methods

This scoping review followed the guidelines set by PRISMA-SCR (Preferred Reporting Items for Systematic Review and Meta-Analysis Extensions for Scoping Reviews) guidelines [44].

### Study selection criteria

Studies were included in the review if they met all of the following criteria: (1) Study Design: Experimental or observational studies; (2) Analytic Approach: Utilized AI technology (e.g., ML, deep learning (DL)) to predict the outcome of basketball games; (3) Research Samples: Focused on professional competitive basketball leagues worldwide (e.g., NBA, Chinese Basketball Association (CBA), Euroleague, Turkish League); (4) Outcomes: Aimed at predicting the results of basketball games (e.g., game outcome, point spread, win percentage); (5) Article Type: Original, empirical, and peer-reviewed journal publications; (6) Time Window of Search: Covered articles published from January 1, 2019 to March 29, 2024, focusing on the last five years; and (7) Language: articles written in English.

Studies were excluded from the review if they met any of the following criteria: (1) Focused on games other than basketball professional leagues (e.g., intramural or intercollegiate competition in high school or college); (2) Centered on predictions unrelated to outcomes (e.g., predicting or evaluating sports performance or sports injuries); (3) Pertained to sports other than basketball (e.g., football, other team sports); (4) Lacked AI technology in the predictive modeling; (5) Failure to report data sources for basketball games; (6) Involved student-athletes rather than professional competitive athletes (e.g., NCAA participants); (7) Were not written in English; (8) Full-text articles or review articles cannot be found; and (9) Were published before 2019.

### Search strategy

A comprehensive keyword search was conducted across four electronic bibliographic databases: PubMed, Web of Science, Scopus, and EBSCO. The search algorithm comprised all possible combinations of keywords from two groups: (1) “artificial intelligence,” “computational intelligence,” “AI,” “machine intelligence,” “computer reasoning,” “machine learning,” “deep learning,” “neural network,” “neural networks,” “reinforcement learning,” and “intelligent systems;” and (2) “basketball.” Additionally, the Medical Subject Headings (MeSH) terms “artificial intelligence” and “basketball” were incorporated into the PubMed search. The detailed search algorithm is provided in S1 File.

Two coauthors independently screened the titles and abstracts of the articles identified through the keyword search, retrieving those that appeared to meet the inclusion criteria, and then thoroughly evaluated the full texts. Additionally, for the included articles, their references and citations were further screened, following a snowballing approach, until no new relevant studies were identified. The interrater agreement between the two coauthors was quantified using Cohen’s kappa (κ = 0.75), indicating substantial agreement. Any disagreements were resolved through discussion.

### Data extraction and synthesis

A standardized data extraction form was used to collect each study’s key methodological and outcome variables. These variables included the first author, year of publication, country, AI techniques used, datasets source, number and types of seasons, number of teams, volume and types of data, model validation approaches, study quality assessment, feature selection, feature extraction, number and types of input features, predicted outcomes, performance metrics, and key findings. During the data synthesis process, following a thematic analysis of the included studies, the results were categorized and synthesized based on various types of predicted match outcomes, including binary outcomes such as win/loss, as well as continuous metrics like winning percentages, final scores, and score differentials. Furthermore, recognizing the significance of the AI methodologies employed, this review systematically synthesized the applied methods, organizing them into broader categories such as general AI approaches, traditional statistical techniques, ML algorithms, and DL models.

### Study quality assessment

We used the National Institutes of Health’s Quality Assessment Tool for Observational Cohort and Cross-Sectional Studies to evaluate the quality of each included study [45]. This instrument scores each study based on 14 criteria. For each criterion, awarding a point for a “yes” response and zero for any other response, including “no,” “not applicable,” “not reported,” or “unable to determine.” By summing up the scores across all criteria, a study-specific overall score ranging from 0 to 14 is derived. This quality assessment of studies aids in gauging the strength of scientific evidence but does not determine the inclusion of studies. Two co-authors of this review independently conducted the quality assessments, with discrepancies resolved through discussion with the third co-author.

## Results

### Identification of studies

**Fig 1:**
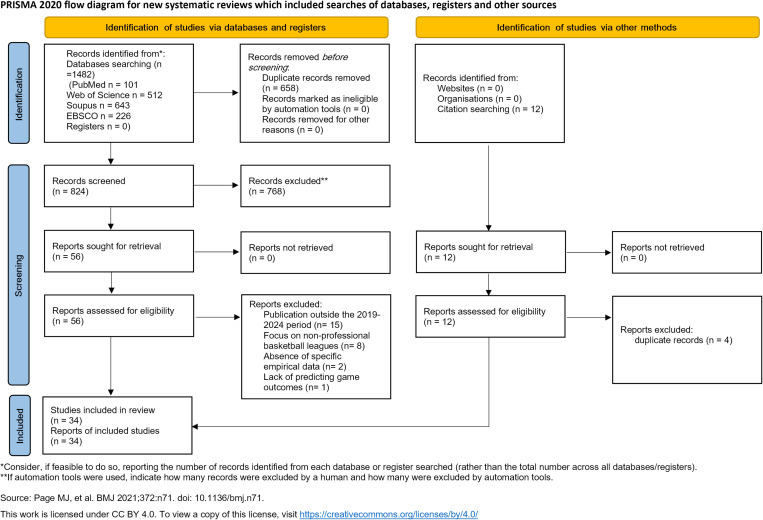
Study selection flowchart.

Fig 1 presents the PRISMA flow diagram. An initial keyword search identified 1,482 articles, and after the removal of duplicates, 824 unique articles remained for screening based on their titles and abstracts. Of these, 768 articles were considered irrelevant and subsequently excluded from the review. The study selection criteria were then applied to the remaining 56 articles, leading to the exclusion of 30 studies for various reasons, such as publication outside the 2019–2024 period (n = 15), focus on non-professional basketball leagues (n = 8), absence of specific empirical data (n = 2), and lack of predicting game outcomes (n = 1). After a comprehensive review of the references and cited literature of the 26 included articles, 8 additional articles were identified as relevant and included in the review. Consequently, a total of 34 articles were ultimately included in this study, guaranteeing a more thorough and in-depth analysis of the subject [1–7,9,20–24,40,41,46–64].

### Study characteristics

**Fig 2:**
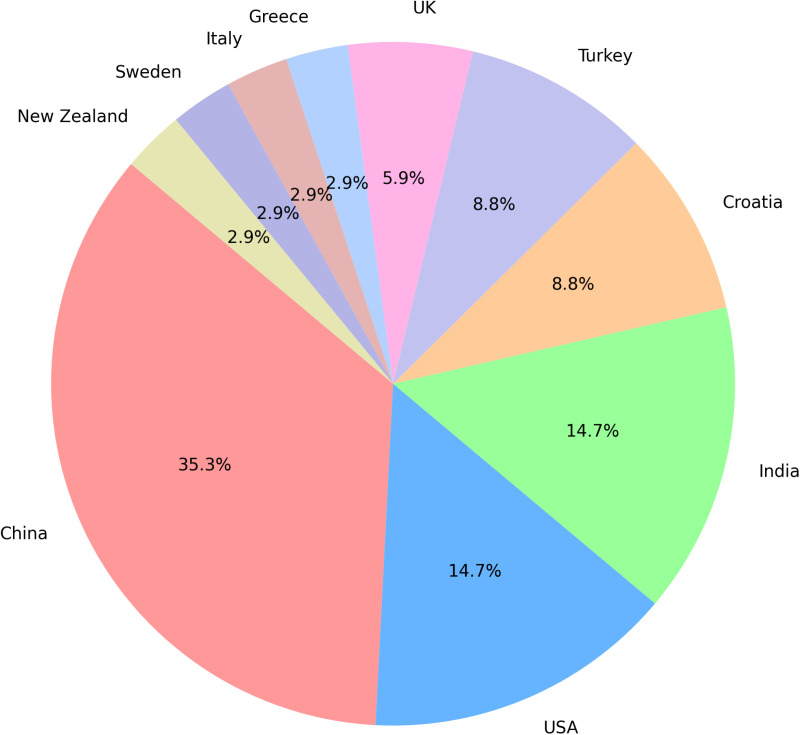
Country frequency distribution.

**Fig 3:**
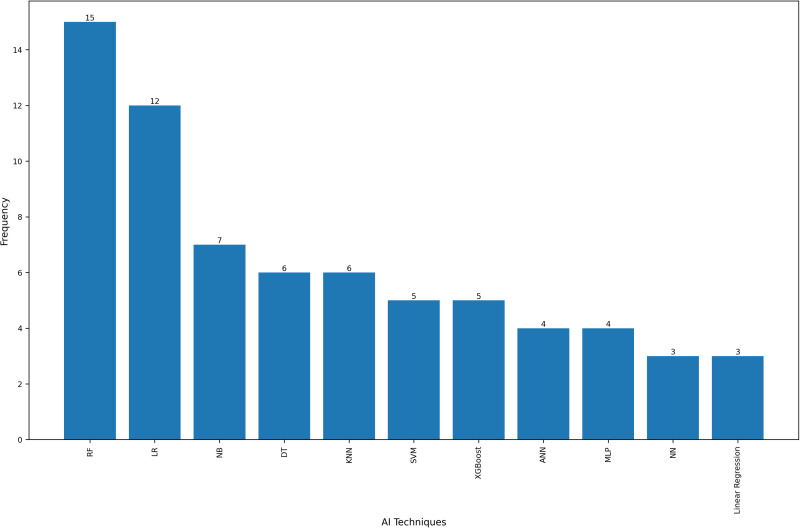
Frequency of AI techniques used (frequency >2).

Table 1 comprehensively summarizes 34 studies that leverage various AI techniques to analyze basketball game data across different countries and periods. These studies were conducted in China (n = 12), the USA (n = 5), India (n = 5), Croatia (n = 3), Turkey (n = 3), the UK (n = 2), New Zealand (n = 1), Italy (n = 1), Sweden (n = 1) and Greece (n = 1) (Fig 2). The studies employ a diverse array of AI techniques, including random forests (RF) (n = 14), logistic regression (LR) (n = 12), NB (n = 7), support vector machines (SVM) (n = 6), KNN (n = 6), linear regression (n = 5), eXtreme Gradient Boosting (XGBoost) (n = 5), among others (Fig 3). Several studies utilize other ensemble methods and hybrid models, combining multiple AI techniques to enhance predictive accuracy and robustness. The primary focus is on NBA data (n = 29), with additional datasets from the CBA (n = 1), EuroLeague (n = 2), the Turkish Basketball Super League (n = 1), and combined datasets from both the NBA and the Turkish league (n = 1).

**Table 1:** Basic information of included studies.

ID	First Author, Year	Country	AI Techniques Used	Datasets source	ID	First Author, Year	Country	AI Techniques Used	Datasets source	ID	First Author, Year	Country	AI Techniques Used	Datasets source
**1**	Alameda-Basora et al. 2019 [4]	USA	Expert Bayesian Network	NBA 2013/2014- 2018/2019	**13**	Ozkan, 2020 [5]	Turkey	ANN, CNFS	Turkish Basketball Super League 2015–2016	**25**	Santos et al. 2022 [58]	Sweden	LR, Linear SVM, RF, MLP	NBA 2008/2009- 2017/2018
**2**	Cai et al. 2019 [3]	China	Hybrid Ensemble Learning	CBA 2016/2017	**14**	Song et al. 2020 [1]	China	Gamma process model, Hybrid Adjustment with Betting Line	NBA 2015/2016- 2017/2018	**26**	Wang et al. 2022 [59]	UK	LIME, RF, FNN	NBA 1980-2019
**3**	Horvat et al. 2019 [20]	Croatia	Naive ML Algorithm	NBA 2009/2010- 2017/2018	**15**	Ballı et al. 2021 [53]	Turkey	KNN, LR, MLP, NB, DT(J48), Voting	EuroLeague 2012/2013- 2016/2017	**27**	Zheng, 2022 [60]	China	RF, NB, SVM, LR, FNN	NBA 2012/2013- 2021/2022
**4**	Kayhan et al. 2019 [46]	USA	Data Snapshot Approach, LSTM, GLM	NBA 2009-2017	**16**	Chen et al. 2021 [40]	Taiwan, China	ELM, MARS, KNN, XGBoost, SGB	NBA 2018-2019	**28**	Daundkar et al. 2023 [61]	India	NB, KNN, RF, DT, LR, SVM	NBA 2018-2019
**5**	Lu et al. 2019 [47]	China	Ordinary Least Squares, Weighted Linear Regression	NBA 2012/2013- 2016/2017	**17**	Lu et al. 2021 [2]	Taiwan, China	CART, RF, SGB, XGBoost, ELM	NBA 2018-2019	**29**	Horvat et al. 2023 [6]	Croatia	LR, NB, DT, MLP, KNN, RF, LogitBoost	NBA 2013/2014- 2017/2018
**6**	Thabtah et al. 2019 [9]	New Zealand	NB, ANN, DT(J48), Logistic Model Tree	NBA 1980-2017	**18**	Chen et al. 2022 [21]	China	Fuzzy Theory	NBA 2018-2019	**30**	Lampis et al. 2023 [62]	Greece	Ensemble Learning, LR, RF, XGBoost	EuroLeague, Eurocup, Greek Basket League, and Spanish Liga ACB 2013-2018
**7**	Yao, 2019 [48]	USA	MLR, NN	NBA 1992-2019	**19**	Khanmohammadi et al. 2022 [54]	USA	Hybrid neural network	NBA 2017/2018- 2021/2022 Iranian Super League playoffs 2020–2021	**31**	Patrot et al. 2023 [24]	India	Linear Regression, SVM, DT	NBA seasons
**8**	Giasemidis, 2020 [49]	UK	LR, SVM with linear and RBF Kernels, DT, RF, NB, GB, KNN, Discriminant Analysis, AdaBoost	EuroLeague 2016/2017- 2018/2019	**20**	Krishnan et al. 2022 [55]	India	LR, ANN	NBA 2010/2011- 2020/2021	**32**	Wang, 2023 [63]	USA	LR, SVM, RF, DNN, RNN (LSTM)	NBA 2004/2005- 2020/2021
**9**	Horvat et al. 2020 [41]	Croatia	LR, NB, DT, MLP, RF, KNN, LogitBoost	NBA 2009/2010- 2017/2018	**21**	Ma et al. 2022 [22]	China	Linear Regression, XGBoost, NN	NBA 2013-2018	**33**	Zhao et al. 2023 [7]	China	GCN, RF	NBA 2012/2013- 2017/2018
**10**	Huang et al. 2020 [50]	Taiwan, China	RT, Linear regression, Support vector regression	NBA 2017/2018	**22**	Osken et al. 2022 [23]	Turkey	ANN, GA, Clustering Techniques	NBA 2012/2013- 2017/2018	**34**	Kandhway, 2024 [64]	India	RF, NN, Support Vector Classifier, LR	NBA 2022/2023
**11**	Li, 2020 [51]	China	Linear Regression, LR, SVM	NBA 2012/2013–2017/2018	**23**	Sikka et al. 2022 [56]	India	Voting Ensembling Regression	NBA 2016/2017- 2020/2021					
**12**	Migliorati, 2020 [52]	Italy	CART, RF	NBA 2004/2005- 2017/2018	**24**	Su et al. 2022 [57]	China	XGBoost, RF, BPNN, GRNN	NBA 2011/2012- 2017/2018					

NBA: National Basketball Association, CBA: Chinese Basketball Association, ML: machine learning, LSTM: Long Short-Term Memory, GLM: general linear model, NB: naive bayes, ANN: artificial neural networks, DT: decision tree, MLR: multiple linear regression, NN: neural network, LR: logistic regression, SVM: support vector machine, RBF kernel: radial basis function kernel, RF: random forest, NB: naive bayes, GB: gradient boosting, MLP: multilayer perceptron, KNN: k-nearest neighbors, CART: classification and regression trees, CNFS: Concurrent Neuro Fuzzy System, ELM: extreme learning machine, MARS: Multivariate Adaptive Regression Splines, XGBoost: eXtreme Gradient Boosting, SGB: Stochastic Gradient Boosting, RNN: recurrent neural network, GA: genetic algorithms, BPNN: backward neural network, GRNN: generalized regression neural network, LIME: Local Interpretable Model-agnostic Explanations, FFNN: feed forward neural network, GCN: graph convolutional network.

These studies span from 1980 to 2024, covering a range of 1–40 seasons, with the majority encompassing multiple seasons. The most-analyzed durations are 1 season and 5 seasons. Notably, two studies present unique cases: one does not specify the number of seasons analyzed, while another selects 5 NBA seasons and 1 Iranian Super League season. The data utilized in these studies are exclusively numeric, mainly from regular seasons. The dataset sizes varying greatly and some not specifying data amounts. Most studies analyze 30 teams, while others focus on different numbers. Typically, 60–90% of data is used for training, with the remainder for testing. Cross-validation, especially 5-fold and 10-fold, is widely employed to ensure robust evaluation. The complete Table 1 is available in S1 Table.

### Outcome measures

Table 2 provides a comprehensive analysis of the feature selection, types of input features, and predicted outcomes utilized in the 34 included studies. The studies employ various feature selection methods to identify the most relevant features from a large dataset. Commonly used methods include statistical-based selection techniques (e.g., Chi-square test, information gain, Gini score), regularization methods (e.g., Least Absolute Shrinkage and Selection Operator (LASSO)), correlation analysis (e.g., Pearson correlation coefficient, mutual information), and more advanced algorithms (e.g., backward elimination, feature importance in RF). Some studies rely on domain knowledge or manual selection to exclude redundant or irrelevant features.

**Table 2:** Feature information, analysis and predicted outcome of included studies.

ID	First Author, Year	Feature Selection Methods	Types of Input Features	The predicted outcome	ID	First Author, Year	Feature Selection Methods	Types of Input Features	The predicted outcome	ID	First Author, Year	Feature Selection Methods	Types of Input Features	The predicted outcome
**1**	Alameda-Basora et al. 2019 [4]	Information gain ratio, Chi-square statistic; Domain knowledge for removing redundant features	***Team statistics***	Total points (Over or Under bet)	**13**	Ozkan, 2020 [5]	Correlation Analysis, Normalization	***Team statistics***	Game winner (win or loss)	**25**	Santos et al. 2022 [58]	Gini impurity for RF, Hyperparameter tuning of the model	***Team statistics*** ***Player statistics*** ***External factors***	Game winner (win or loss), championship winners
**2**	Cai et al. 2019 [3]	Bagging Strategy and Random Subspace Method	***Team statistics***	Game winner (win or loss)	**14**	Song et al. 2020 [1]	Historical Data Integration	***Team statistics***	Total points (Over or Under bet)	**26**	Wang et al. 2022 [59]	Domain knowledge for removing redundant features, attribute aggregation	***Team statistics***	Monthly win ratio (game winning percentage), Post-season playoff qualification
**3**	Horvat et al. 2019 [20]	NA	***Team statistics***	Game winner (win or loss)	**15**	Ballı et al. 2021 [53]	NA	***Team statistics***	Game winner (win or loss)	**27**	Zheng, 2022 [60]	Sequential Forward Selection, Recursive Feature Elimination	***Team statistics***	Game winner (win or loss)
**4**	Kayhan et al. 2019 [46]	Elastic Net regularization	***Team statistics***	End-of-game point spread	**16**	Chen et al. 2021 [40]	MARS, XGBoost, SGB	***Team statistics***	Final scores	**28**	Daundkar et al. 2023 [61]	Chi-square Test, Gini Scores from RF	***Team statistics***	Game winner (win or loss)
**5**	Lu et al. 2019 [47]	NA	***Team statistics***	Point differences between the H/A teams	**17**	Lu et al. 2021 [2]	NA	***Team statistics***	Final scores	**29**	Horvat et al. 2023 [6]	Information Gain	***Team statistics*** ***Player statistics***	Game winner (win or loss)
**6**	Thabtah et al. 2019 [9]	Multiple Regression, Correlation Feature Set, and RIPPER algorithm	***Team statistics*** ***Player statistics***	Game winner (win or loss)	**18**	Chen et al. 2022 [21]	Fuzzy Theory analysis	***Team statistics***	Game winner (win or loss)	**30**	Lampis et al. 2023 [62]	LR with Regularization, RF, XGBoost	***Team statistics***	Game winner (win or loss)
**7**	Yao, 2019 [48]	Backward Elimination, Correlation Matrix Analysis	***Team statistics***	Winning percentages of NBA teams	**19**	Khanmohammadi et al. 2022 [54]	Feature Imitating Networks	***Team statistics*** ***Player statistics***	Playoff game winner (win or loss)	**31**	Patrot et al. 2023 [24]	Correlation Feature Set, Multiple Regression	***Team statistics***	Game winner (win or loss)
**8**	Giasemidis, 2020 [49]	ANOVA F-test, Mutual Information, Chi-Square Test, Wrapper Methods	***Team statistics***	Game winner (win or loss)	**20**	Krishnan et al. 2022 [55]	Correlation Analysis	***Team statistics*** ***Player statistics***	Win-loss percentage	**32**	Wang, 2023 [63]	Correlation-based filtering	***Team statistics***	Game winner (win or loss)
**9**	Horvat et al. 2020 [41]	NA	***Team statistics***	Game winner (win or loss)	**21**	Ma et al. 2022 [22]	Pearson Correlation Coefficient, weighted averaging	***Player statistics*** ***External factors***	Game winner (win or loss)	**33**	Zhao et al. 2023 [7]	LASSO,RF	***Team statistics***	Game winner (home win or away win)
**10**	Huang et al. 2020 [50]	Chi-Square Test	***Player statistics***	Game scores for individual players and the total team score	**22**	Osken et al. 2022 [23]	Clustering Analysis	***Team statistics*** ***Player statistics***	Game winner (win or loss)	**34**	Kandhway, 2024 [64]	Manual selection based on domain expertise	***Team statistics***	Game winner (win or loss)
**11**	Li, 2020 [51]	LASSO, Correlation Matrix Analysis	***Team statistics***	Game winner (win or loss)	**23**	Sikka et al. 2022 [56]	Correlation Heatmap Analysis	***Team statistics***	Team’s win percentage over the course of a season					
**12**	Migliorati, 2020 [52]	Gradually eliminate irrelevant or secondary variables	***Team statistics***	Game winner (Win or loss) for Golden State Warriors	**24**	Su et al. 2022 [57]	Correlation Analysis	***Player statistics***	Player scores (Points per game)					

NA: not applicable, RIPPER: Repeated Incremental Pruning to Produce Error Reduction, ANOVA: Analysis of Varianc, LASSO: Least Absolute Shrinkage and Selection Operator, MARS: Multivariate Adaptive Regression Splines, XGBoost: eXtreme Gradient Boosting, SGB: Stochastic Gradient Boosting, RF: random forest, LR: logistic regression.

**Fig 4:**
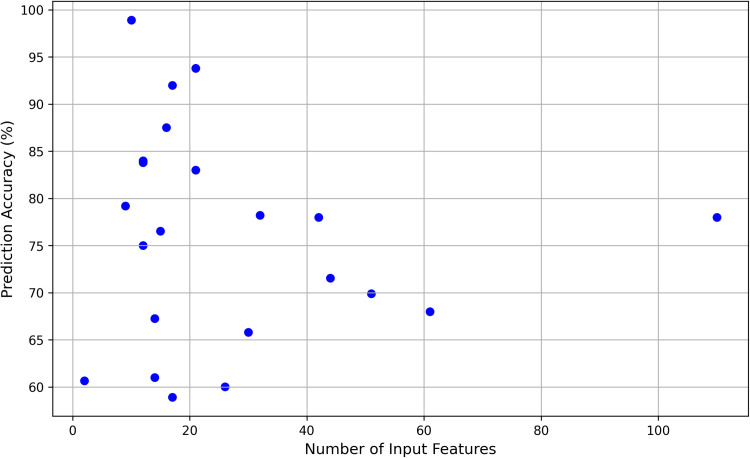
Relationship between input features and prediction accuracy.

The input features primarily fall into three categories: team statistics, player statistics, and external factors related to the game schedule. Team statistics include points scored, shooting percentages, and defensive efficiency; player statistics encompass individual performance indicators like points, assists, rebounds, and fouls; external factors involve contextual elements like home-court advantage, season stage, and rest days. Some studies integrate advanced statistical models and metrics, such as Elo ratings, PageRank, and player impact ratings. As shown in Fig 4, the scatter-plot presents the relationship between the number of input features and the prediction accuracy. It can be seen from the figure that the relationship between the two is rather complex and not a simple linear one. When the number of input features is at a low level (around 0–20), the prediction accuracy fluctuates significantly, ranging widely from 58% to 99%. As the number of input features increases to the range of 20–60, the prediction accuracy is relatively concentrated between 65% and 80%. When the number of features exceeds 60, the number of sample points decreases, and the prediction accuracy roughly fluctuates between 65% and 80%.

The primary predicted outcomes include game-winner (win or loss), winning percentages of teams, game scores, and difference in scores. Specifically, game-winner predictions were made in 22 studies, winning percentages of teams were predicted in 4 studies, game scores were predicted in 6 studies, and differences in scores were predicted in 2 studies. The complete Table 2 is available in S2 Table.

### Main findings

**Fig 5:**
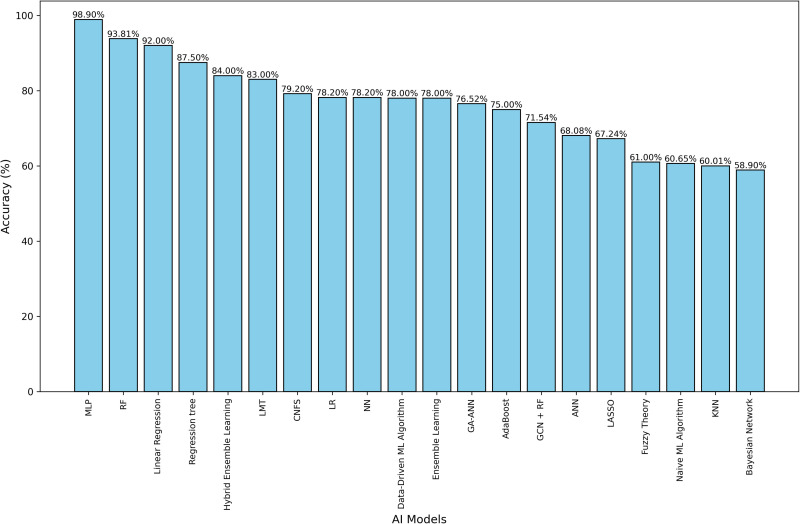
Comparison of different AI models’ accuracy.

Table 3 summarizes the performance metrics and key findings of the studies included in this review. Four primary themes emerged from the analysis: game-winner predictions, team-winning percentages, game scores, and point differences. These themes reflect the diverse applications of AI models in predicting various aspects of basketball game outcomes. The complete Table 3 is available in S3 Table, Fig 5.

**Table 3:** Performance metrics and key findings of included studies.

ID	First Author, Year	Performance Metrics	ID	First Author, Year	Performance Metrics	ID	First Author, Year	Performance Metrics
**1**	Alameda-Basora et al. 2019 [4]	**Accuracy:** • Expert Bayesian Network: Overall accuracy of 58.9% • Non-Expert Bayesian Network: Overall accuracy of 44.8%	**13**	Ozkan, 2020 [5]	**ANN Model:** • Accuracy: 70.8% • Sensitivity: 54.5% • Specificity: 84.6% **CNFS Model:** • Accuracy: 79.2% • Sensitivity: 72.7% • Specificity: 79.1%	**25**	Santos et al. 2022 [58]	**Accuracy** • LR: 68.58% • Linear Support Vector Machines: 68.18% • RF: 69.88% • MLP: 68.85%
**2**	Cai et al. 2019 [3]	**Hybrid Ensemble Learning Framework:** **Accuracy:** 84% **F1-score:** 82%	**14**	Song et al. 2020 [1]	**Accuracy:** • Gamma process model: Outperforms naive model based on empirical frequency • Adjusted gamma process model: Higher accuracy than naive model and gamma process model for regular games	**26**	Wang et al. 2022 [59]	**For Win Ratio Prediction:** • RF: R² = 0.65, MSE = 0.014 • FFNN: R² = 0.77, MSE = 0.009 **For Post-season Playoff Classification:** • RF: F1 = 0.85 • FFNN: F1 = 0.87
**3**	Horvat et al. 2019 [20]	**Accuracy:** *Using all played games during the training phase:* • 2016 training period, 2017 evaluation: 58.84% (training) and 60.23% (including evaluation phase) • 2015 training period, 2016–2017 evaluation: 59.25% (training) and 60.48% (including evaluation phase) • 2014 training period, 2015–2017 evaluation: 59.41% (training) and 60.65% (including evaluation phase) *Using only mutual games during the training phase:* 2014 training period, 2015–2017 evaluation: 58.90% (training) and 60.27% (including evaluation phase)	**15**	Ballı et al. 2021 [53]	**Accuracy:** • MLP: 98.90% (Dataset 5 and Model 6) • LR: Various accuracies, up to 98.04% depending on dataset and model	**27**	Zheng, 2022 [60]	**Accuracy:** • Best RF model: 67.98% • LR with Sequential Forward Selection on Feature Set C: 67.39% • NB with SFS on Feature Set C: 67.48% • FFNN on Feature Set B: 67.58% • NB on Feature Set B: 67.88%
**4**	Kayhan et al. 2019 [46]	**MAE:** • Data snapshot approach, LSTM network, and GLM had nearly identical MAE values • Initial MAE: ~ 11 points at the beginning of games • Half-time MAE: slightly above 8 points • Final minute MAE: within 2 points	**16**	Chen et al. 2021 [40]	**MAPE** Single models: • ELM: 0.0870 • MARS: 0.0846 • XGBoost: 0.0842 • SGB: 0.0845 • KNN: 0.0873 Two-stage models: • ELM: 0.0863 • MARS: 0.0845 • XGBoost: 0.0818 • SGB: 0.0829 • KNN: 0.0872 **RMSE:** • Best two-stage XGBoost model: 11.4753 **Sum of Squared Errors (SSE)**: • Best two-stage XGBoost model: 61,627.37	**28**	Daundkar et al. 2023 [61]	**Accuracy:** 2018 season: • RF: 60.8% to 65.5% • LR: 62.3% to 65.8% 2019 season: • RF: 54.9% to 61.3% • LR: 57.1% to 61.3%
**5**	Lu et al. 2019 [47]	**Standard deviation between the actual point difference and predicted point difference:** • One-year prediction: 15.32 for playoff games, 12.43 for regular season games • Out-of-sample prediction: 12.44 • Weighted prediction: Best result with a standard deviation of 11.97555, slope 0.91, and p-value 0.06	**17**	Lu et al. 2021 [2]	**RMSE:** • CART: Best RMSE = 11.7564 • RF: Best RMSE = 11.6303 • SGB: Best RMSE = 11.5586 • XGBoost: Best RMSE = 11.6941 • ELM: Best RMSE = 11.8020	**29**	Horvat et al. 2023 [6]	**Accuracy** • Average accuracy: 66% • Maximum accuracy: 78%
**6**	Thabtah et al. 2019 [9]	**Accuracy (Dataset D)** • NB: 80% • ANN: 80% • LMT: 83% **Precision, Recall, F1 Score from Dataset D:** All three metrics were highest for LMT at 83% each	**18**	Chen et al. 2022 [21]	**Accuracy:** 61% using fuzzy theory	**30**	Lampis et al. 2023 [62]	**Brier Score, F1-score, and accuracy** **Best performing model accuracy:** Greek Basket League: 78% Spanish Liga ACB: 72% Euroleague: ⁓ 69% Eurocup: ⁓ 69%
**7**	Yao, 2019 [48]	**RMSE:** • Linear Regression Model: 0.047 • NN Model-1 (14 parameters): 0.043 • NN Model-2 (64 parameters): 0.035 • Ensemble learning (combined model): 0.034 **R²:** • Ensemble learning (combined model): 0.954	**19**	Khanmohammadi et al. 2022 [54]	**Area Under the ROC Curve:** • MambaNet with team and player statistics: AUC ranged from 0.72 to 0.82 across different NBA seasons and datasets	**31**	Patrot et al. 2023 [24]	**Accuracy:** • Linear Regression: 92% • SVM: 85% • DT: 69%
**8**	Giasemidis, 2020 [49]	**Accuracy:** • Simple ML models: Not greater than 67% on the test set • AdaBoost model: 75% accuracy using 5-fold cross-validation, 66.8% accuracy on hold-out set	**20**	Krishnan et al. 2022 [55]	**Accuracy:** • LR: 66.24% • ANN: 68.08%	**32**	Wang, 2023 [63]	**Accuracy:** • LR: 81.64% • SVM: 74.81% • LSTM: 75.31% • RF: 83.78% **AUC (Area Under the Curve):** • LR: 0.90 • SVM: 0.82 • LSTM: 0.83 • RF: 0.92
**9**	Horvat et al. 2020 [41]	**Accuracy:** *Train&Test validation method:* • KNN: 57.90% (best) • DT: 53.49% (worst) *Cross-validation method:* • KNN: 58.95% (best) • DT: 53.37% (worst) *Up-to-date data with Train&Test validation method:* • KNN: 60.01% (best) • DT: 54.66% (worst)	**21**	Ma et al. 2022 [22]	**RMSE:** • Linear Regression: 9.2558 • XGBoost: 8.9581 • NN: 9.0387 **MAE:** • Linear Regression: 7.0478 • XGBoost: 6.8486 • NN: 6.8805	**33**	Zhao et al. 2023 [7]	**Accuracy:** GCN alone: 66.90% GCN + RF: 71.54% GCN + LASSO: 70.70% GCN + PCA: 50.73%
**10**	Huang et al. 2020 [50]	**RMSE:** • Regression Tree (M5P): 0.9645 • Linear Regression: 1.3081 • Support Vector Regression: 2.4904 **Accuracy:** Regression tree: 87.5%	**22**	Osken et al. 2022 [23]	**Accuracy:** • GA-ANN using c-means clustering with cosine distance: 76.52% • GA-ANN using k-means clustering with Euclidean distance (k = 26): 76.29% • GA-ANN using k-means clustering with Euclidean distance (k = 25): 75.36% • Naive predictor (home team wins): 58% • Human experts: 65%−68%	**34**	Kandhway, 2024 [64]	**Accuracy** Varies by quarter and classifier • RF: 61.5% (Quarter 1) to 69.8% (Quarter 4) • SVM: 54.2% (Quarter 1) to 77.3% (Quarter 4) • NN: 59.0% (Quarter 1) to 78.2% (Quarter 4) • LR: 58.5% (Quarter 1) to 78.2% (Quarter 4)
**11**	Li, 2020 [51]	**Accuracy** • Linear Regression: ✓ 2015: 66.91%, 2016: 63.80%, 2017: 64.31% • LR: ✓ 2015: 67.15%, 2016: 63.34%, 2017: 64.23% • SVM: ✓ 2015: 66.34%, 2016: 63.73%, 2017: 64.23% Accuracy after LASSO: ✓ 2015: 67.24%, 2016: 63.93%, 2017: 65.45%	**23**	Sikka et al. 2022 [56]	**R² and RMSE for various models:** • Linear Regression + RF: R² = 0.9298, RMSE = 0.0373 • RF + Gaussian Process: R² = 0.9332, RMSE = 0.0365 • Final Ensemble (all five models): R² = 0.9332, RMSE = 0.0364			
**12**	Migliorati, 2020 [52]	**Accuracy:** • CART (Box Score, without points and assists): 71.68% • CART (Four Factors, without shooting): 67.26% • RF (Box Score, without points and assists): 91.15% • RF (Four Factors): 93.81%	**24**	Su et al. 2022 [57]	**RMSE:** • XGBoost: 15.92 (initial set), 15.17 (filtered set) • RF: 23.84 (initial set), 26.12 (filtered set) • BPNN: 98.88 (initial set), 98.88 (filtered set) • GRNN: 381.28 (initial set), 54.11 (filtered set) **MAPE:** • XGBoost: 2.44 (initial set), 2.13 (filtered set) • RF: 5.75 (initial set), 5.16 (filtered set) • BPNN: 16.44 (initial set), 22.71 (filtered set) • GRNN: 215.70 (initial set), 11.66 (filtered set)			

SVM: support vector machine, RBF kernel: radial basis function kernel, ML: machine learning, LSTM: Long Short-Term Memory, GLM: general linear model, MAE: mean absolute error, NB: naive bayes, ANN: artificial neural networks, LMT: logistic model tree, RIPPER: Repeated Incremental Pruning to Produce Error Reduction, NN: neural network, RMSE: root mean square error, KNN: k-nearest neighbors, DT: decision tree, LR: logistic regression, LASSO: Least Absolute Shrinkage and Selection Operator, CART: classification and regression trees, RF: random forest, CNFS: Concurrent Neuro Fuzzy System, MLP: multilayer perceptron, MAPE: mean absolute percentage error, SGB: stochastic gradient boosting, XGBoost: eXtreme gradient boosting, MARS: Multivariate Adaptive Regression Splines, ELM: extreme learning machine, ROC: receiver operating characteristic, AUC: area under the curve, RNN: recurrent neural network, GA: genetic algorithms, BPNN: backward neural network, GRNN: generalized regression neural network, MSE: mean square error, FFNN: feed forward neural network, LIME, Local Interpretable Model-agnostic Explanations, SFS: Selective Feature Selection, GCN: graph convolutional network, PCA: Principal component analysis.

#### Game winner (win or loss).

Over the past five years, the application of AI models in predicting the outcomes of sports competitions has garnered widespread attention. The most commonly used models include RF, SVM, and ANN. The multilayer perceptron (MLP) model achieved the highest accuracy at 98.9% [53], while RF models also performed strongly, up to 93.8% [52]. Traditional models like linear regression showed lower performance, with accuracies between 63.3% and 67.2% [51].

Feature selection significantly enhances model accuracy, with methods like LASSO and multiple regression improving performance by identifying key features such as field goal percentage and defensive rebounds [9,51]. However, model adaptability across different datasets remains a challenge. For instance, the AdaBoost model’s accuracy dropped from 75% during cross-validation to 66.8% on an independent test set [49], underscoring the need for further validation and optimization across diverse scenarios. Predictive accuracy was improved to 78% by dynamically adjusting training data based on an extended team efficiency index, enhancing the model’s practical applicability [6]. However, broader application and generalization across diverse datasets still require further validation and optimization.

#### Winning percentages of teams.

Several studies have shown that AI models, such as neural networks (NN) and ensemble learning models, excel in predicting NBA winning percentages. For example, NN with 64 parameters outperformed traditional linear regression, achieving a root mean square error (RMSE) of 0.034 and an R² of 0.954 [48]. Similarly, an ANN model gained 68.08% accuracy, surpassing linear regression’s 66.24% [55]. Ensemble models further enhanced accuracy, achieving an R² of 0.9332 and an RMSE of 0.0364 [56].

Feature selection and model optimization were crucial for improving model performance. Methods like one-hot encoding and selecting key features such as point differential and offensive efficiency significantly enhanced predictions [55,56]. Additionally, the adaptability of these models was validated in dynamic environments [59], with ensemble methods proving particularly effective in practical applications [56].

#### Game scores.

XGBoost is widely recognized as one of the best-performing models for predicting game scores. Studies have consistently shown its efficiency in handling large datasets and complex features. For example, a two-stage XGBoost model achieved a mean absolute percentage error (MAPE) of 0.0818, outperforming other models [40]. Similarly, after feature selection, XGBoost’s MAPE improved from 2.44 to 2.13, further validating its accuracy [57]. Another study confirmed XGBoost’s superior performance when weighted by lagged game information [2].

Feature selection is critical for optimizing model performance. Incorporating nonlinear features and domain knowledge significantly enhanced predictive accuracy [4,50]. Optimizing feature sets is essential for improving model efficiency and effectiveness in practical applications. To enhance adaptability and practicality, studies explored integrating real-time data and adaptive features, leading to more reliable predictions in dynamic environments [1,2]. These improvements suggest that XGBoost and similar models can provide accurate predictions even in the complex and rapidly changing context of sports competitions.

#### Point difference.

AI technologies have shown significant potential in predicting point differentials in basketball games, with each model exhibiting unique strengths and weaknesses. A study compared data snapshot methods, Long Short-Term Memory (LSTM) and Generalized Linear Models (GLM) for predicting point differentials [46]. While the initial prediction error was around 11 points, it decreased to approximately 2 points by the game’s final minute [46]. Despite comparable accuracy to LSTM and GLM, the data snapshot method offered greater computational efficiency, making it ideal for real-time applications like betting and in-game coaching decisions [46]. A linear regression model incorporating team ability and home advantage was used, reducing the prediction error to 11.98 points after weighted adjustments [47]. This model effectively predicted the outcomes of 13 out of 15 games during the Golden State Warriors’ 2016−17 season [47]. However, factors such as player injuries, trades, and coaching changes can affect these models’ accuracy, highlighting the importance of integrating dynamic variables for more accurate predictions [47].

## Methodological review

### Overview of AI

In many sports, predicting the outcome of professional basketball games has become one of the highlights of AI technology’s powerful capabilities, revolutionizing a task traditionally relying on human intuition and experience. The successful application of AI technologies such as ML and DL has significantly enhanced prediction accuracy in this field with its powerful data processing and analysis capabilities.

### Traditional statistical approaches

Traditional statistical methods, including linear regression, LR, and multiple linear regression (MLR), remain crucial in basketball game outcome prediction due to their simplicity, interpretability, and effectiveness in capturing linear relationships. While these methods may struggle with complex, nonlinear data compared to ML approaches, they still offer significant predictive power in specific scenarios.

#### Linear regression.

Linear regression is commonly used to predict continuous outcomes like point differentials or team win rates. A study found that while linear regression effectively modeled linear relationships, its accuracy declined with nonlinear features [48]. Another study showed that linear regression was practical during regular season predictions but less accurate in high-pressure playoff environments [47].

#### Logistic regression.

LR is widely used for binary classification tasks, such as predicting game wins or losses. Studies demonstrated LR’s reasonable accuracy, although more complex models like RF often perform better [58,63]. LR’s strength lies in its simplicity and robustness across various datasets.

#### Multiple linear regression.

The application of MLR in NBA game outcomes prediction involves analyzing offensive and defensive metrics to model team performance. Yao (2019) demonstrated that MLR models effectively identified critical variables such as field goal percentage and defensive parameters, achieving reliable predictions after addressing multicollinearity through parameter elimination and VIF validation [48]. However, MLR’s linear assumptions limited its accuracy compared to NNs when handling nonlinear relationships in larger datasets [48]. In contrast, Sikka (2022) integrated MLR into an ensemble model with other algorithms, where it contributed interpretability by highlighting key predictors like points per game and efficiency differentials, though the ensemble’s superior accuracy stemmed from combining MLR’s transparency with non-linear methods [56].

### Machine learning

ML techniques can be categorized into unsupervised learning and supervised learning. For predicting game outcomes, supervised learning methods are primarily used to predict specific results such as win/loss and game scores. Unsupervised learning is typically utilized for data preprocessing and feature extraction to enhance the performance of prediction models. This section summarizes the use of various supervised learning algorithms in the included studies, focusing on RF, KNN, SVM, DT, and Ensemble learning.

#### Random forests.

RF, an ensemble learning method based on DT, has demonstrated strong performance in predicting basketball outcomes. Studies have shown RF’s best accuracy achieved 93.81% in predicting game results and team win rates [52]. RF is also effective in feature selection and model optimization, where combining Sequential Forward Selection and Recursive Feature Elimination (RFE) improved prediction accuracy to 67.98% [60].

RF is often used in hybrid models to enhance predictive capabilities. Studies successfully integrated RF with other algorithms, achieving high prediction accuracies across various basketball leagues [62]. Another study further demonstrated the benefits of combining RF with Graph Convolutional Networks (GCN), improving prediction accuracy at 71.54% by considering spatial structures [7]. RF’s dynamic prediction capability is another key advantage. RF’s accuracy improved from 61.5% at the start of a game to 69.8% by the fourth quarter, highlighting its potential for real-time analysis [64].

#### K-Nearest neighbors.

KNN is an instance-based, non-parametric algorithm known for its simplicity and effectiveness, particularly in predicting basketball game outcomes. KNN’s core principle involves measuring the distance to the nearest neighbors and making predictions through voting or averaging, making it well-suited for small datasets with well-defined features.

A study demonstrated that KNN’s accuracy ranged from 57.90% to 60.01%, with improvements in incorporating recent game data [41]. Despite a slightly higher MAPE than models like XGBoost, KNN still shows strong predictive capabilities [40].

However, KNN’s computational cost increases with larger datasets, highlighting the need for optimization or combining it with other algorithms in large-scale applications. KNN’s flexibility allows it to adapt to various datasets and tasks, particularly when enhanced by feature selection, cross-validation, and hybrid models [40,41,61].

#### Support vector machines.

SVMs are widely used in predicting basketball game outcomes due to their ability to handle high-dimensional and nonlinear data. SVMs achieve precise classification by maximizing the margin between classes. For instance, an SVM with a Radial Basis Function Kernel achieved 84% accuracy, outperforming traditional methods [3]. While SVM’s accuracy is slightly lower than RF [63], it excels in managing nonlinear feature relationships.

Feature selection further enhances SVM performance. For instance, in basketball outcome prediction studies, Li (2020) demonstrated that applying LASSO-based feature selection to SVM significantly improved accuracy by prioritizing high-impact metrics such as defensive rebounds, opponent free throws attempted, and home/away team assists, which effectively reduced model noise and amplified discriminative patterns [51]. Zheng (2022) further corroborated this by showing SVM models integrated with sequential forward selection on hybrid features (e.g., tiredness-adjusted Elo ratings and recent performance differentials) achieved enhanced generalization, as feature pruning mitigated overfitting while emphasizing latent interactions between team fatigue dynamics and skill metrics [60]. These studies collectively affirm that targeted feature engineering and selection optimize SVM’s capacity to delineate critical decision boundaries in complex, high-dimensional sports datasets.

#### Decision trees.

DTs are widely used in predicting basketball game outcomes due to their intuitive and interpretable nature. DTs build a tree-like model by recursively partitioning data, making them practical for classification and regression tasks. Although they are prone to overfitting and data noise, their simplicity and interpretability have made them valuable in various studies.

It was found that while DTs had slightly lower accuracy (53.37% to 54.66%) compared to more complex models like KNN, their performance improved with up-to-date game data [41]. Similarly, Patrot (2023) highlighted that despite DT achieving a relatively lower accuracy (69%) than other models such as Linear Regression (92%) and SVM (85%), they proved effective in identifying critical game-influencing features—such as defensive rebounds and three-point percentage —through feature selection, which enhanced their utility for strategic analytics despite their modest prediction rates [24]. DTs are flexible in feature selection and data processing, and they can effectively assess the importance of features, making them particularly useful for quickly assessing the importance of multiple features. Combing DT-based methods performance with feature selection enhances predictive capabilities for critical features like defensive metrics [7].

DTs are often integrated into hybrid models to boost predictive performance. It has been demonstrated that while DTs’ standalone performance might be limited, they enhance decision-making processes when combined with other models, improving overall accuracy in ensemble frameworks [56].

#### Ensemble learning.

Ensemble learning is a powerful technique for predicting basketball game outcomes, combining multiple models to enhance accuracy, robustness, and generalization. Strategies like Bagging, Boosting, and Stacking have proven effective in handling complex, multidimensional data.

For example, an 84% prediction accuracy was achieved using a hybrid ensemble framework [3]. The stability of integrating models like RF, LR, and XGBoost across various leagues was demonstrated [62]. Ensemble learning also plays a crucial role in feature selection and model optimization, as seen in a study that combined multiple algorithms to predict NBA win rates with high accuracy (R² = 0.9332) [56]. Comparative model analyses in NBA studies reveal how heterogeneous algorithms like RF (dependent on historical player metrics) and LR (focused on short-term dynamics) provide complementary strengths, offering a foundation for ensemble frameworks to address playoff complexity. Simultaneously, novel features such as fatigue quantification [60] and multi-model optimized input designs [58] further validate ensemble adaptability in capturing contextual and temporal interactions.

### Deep learning

To achieve more accurate predictions of basketball game outcomes, an increasing number of studies have employed DL techniques to process and analyze game-related data. The following DL approaches have been widely utilized: (1) LSTM: A variant of RNN, LSTM is particularly effective in handling dependencies in time series data, making it suitable for capturing dynamic game changes over time. However, its computational efficiency can be a challenge for real-time applications. Kayhan (2019) compared LSTM with General Linear Models for predicting point spreads and found that while both models performed well, LSTM was computationally less efficient, making real-time decision-making more difficult [46]. (2) GCN: Designed for processing graph-structured data, GCNs are particularly effective in capturing relationships between players and team structures. This makes them well-suited for modeling complex interaction patterns in basketball games. Zhao et al. (2023) combined GCN with RF, achieving a prediction accuracy of 71.54%, demonstrating the model’s ability to enhance game outcome predictions by leveraging relational data [7]. (3) MLP: A fully connected NN model, MLP is commonly used for game outcome prediction due to its ability to learn patterns from structured numerical data. However, its performance can vary based on the dataset and model configuration. Balli and Ozdemir (2021) reported that MLP achieved an impressive 98.90% accuracy in predicting EuroLeague outcomes [53], whereas Santos et al. (2022) found that RF slightly outperformed MLP in NBA game predictions, with respective accuracies of 69.88% and 68.85% [58]. (4) Hybrid Neural Networks: These models integrate multiple DL techniques to leverage their combined strengths, improving predictive accuracy in complex datasets. Khanmohammadi et al. (2022) introduced MambaNet, a hybrid neural network architecture that combines different DL models [54]. Their study demonstrated that MambaNet achieved superior performance in basketball game prediction, with AUC scores ranging from 0.72 to 0.82 [54].

### Study quality assessment

S4 Table reports criterion-specific and global ratings from the study quality assessment. On average, the included studies scored 10 out of 14 (range: 9–11). All studies clearly stated their research questions or objectives, specified and defined their study populations, maintained a participation rate of at least 50%, uniformly applied inclusion and exclusion criteria to participants, maintained an attrition rate of 20% or less, measured exposures prior to outcomes, ensured clear definition and consistent application of outcome measures, and implemented clearly defined, valid, and reliable exposure measures consistently across participants. Most studies had a sufficient timeframe to observe an association between exposure and outcome (n = 27). Only a few studies (n = 7) statistically adjusted for key potential confounding variables, and even fewer (n = 2) assessed exposures more than once over time. However, none of the studies examined different levels of exposure in relation to the outcomes, nor did they have outcome assessors blinded to the exposure status of participants.

## Discussion

This study systematically reviewed the application of AI techniques in predicting the outcomes of professional basketball games. The findings indicate that AI models are highly effective in predicting basketball game outcomes, often surpassing traditional statistical methods in accuracy. Studies focused on achieving higher accuracy by comparing different AI models and employing feature selection and extraction methods before algorithm application. The review of 34 articles revealed a growing trend in adopting advanced ML models to tackle these complex tasks. However, the effectiveness of AI in predicting basketball game outcomes is influenced by several factors, which are discussed in the following sections.

### Comparative analysis of AI techniques and their effectiveness

This review reveals that the effectiveness of AI techniques in predicting basketball game outcomes largely depends on the quality of the data, the relevance of the selected features, and the available computational resources. Studies have shown that, although RF models using four factors can achieve up to 93.81% accuracy [52] but much lower in others. This suggests that while the RF model is robust, it may still be prone to overfitting when the data distribution or features differ [65,66]. Moreover, the success of AI models like NN and XGBoost often depends more on the quality and relevance of input features than on the complexity of the models themselves. While advanced methods, such as combining GCNs with RF, have improved accuracy (up to 71.54%), they have also increased model complexity and computational demands [7]. This underscores the urgency of developing models that are both accurate and computationally efficient.

Simple models like KNN are attractive for small datasets, but their computational intensity makes them impractical for large-scale datasets [41]. Similarly, while SVMs are effective for handling high-dimensional data, their stringent requirements for hyperparameter tuning limit their use in real-time decision-making [51]. Ensemble and hybrid models often provide robust predictions by combining multiple AI techniques [67], though they add complexity and may be less suited for real-time applications [3,40].

Given that the trade-off between model complexity and computational efficiency is particularly critical in real-time applications requiring rapid decision-making, future research should focus on developing techniques such as model pruning, quantization, and hardware-specific optimizations (e.g., graphics processing unit acceleration) to maintain high predictive performance while reducing computational load [68]. Additionally, multi-task learning models, which can predict multiple related outcomes simultaneously, offer a way to maximize data utilization and capture the complex relationships between different aspects of a game, thereby enhancing model adaptability and improving their generalization across different leagues [69].

### Impact of data processing and feature selection on model performance

Effective data processing and feature selection are crucial for enhancing AI model performance in predicting basketball game outcomes—data processing steps like cleaning, normalization, and feature engineering influence model accuracy and robustness. Handling missing values and outliers is essential for maintaining data quality, while normalization techniques, such as standardization, improve prediction consistency and reliability [5,22]. Moreover, the ability to process and integrate unstructured data is also crucial. In addition to structured data such as player statistics and game scores, there is a vast amount of unstructured data (e.g., game videos, social media posts, and basketball news). For instance, applying convolutional neural networks to video data can identify specific players or evaluate player movements [70], while using natural language processing to analyze coach comments or player interviews can reveal psychological factors [71] that may affect game performance, thereby enhancing predictions by integrating this information with structured data.

Feature selection significantly enhances model performance by identifying the most relevant features [72]. Methods like Information Gain, Chi-Square Test, and RFE have proven effective in improving computational efficiency and reducing overfitting [6,60,61]. However, it was also shown that traditional feature selection methods, such as Information Gain and Chi-Square Test, typically focus on the statistical significance of individual features, potentially overlooking complex interactions among multiple variables [73]. In contrast, model-based feature selection methods, such as DT and RF, inherently account for interactions between features, leading to more accurate predictions of game outcomes [74,75]. ML approaches like Generalized Additive Models can also effectively model nonlinear interactions between variables [76], which is particularly important in basketball, where outcomes are often determined by the combined effects of multiple factors. Future research should focus on further developing and optimizing feature selection methods that can capture feature interactions to improve the predictive accuracy of models.

The review found that feature sets ranging from 2 to 110 features often provided the best balance between accuracy and model complexity, with accuracy rates between 53.4% and 98.9%. While larger feature sets can sometimes marginally improve accuracy, they also increase model complexity, making them less practical for real-time applications [48]. Feature selection also enhances computational efficiency and interpretability, which is critical for real-time applications where resources are limited [64].

In conclusion, data processing and feature selection are foundational for developing effective AI models in basketball game prediction. These processes improve accuracy, efficiency, and interpretability, making models more applicable in real-world scenarios. Future research should focus on balancing model complexity and performance to optimize AI models in sports analytics.

### Methods of model validation and performance metrics

Effective model validation and performance evaluation are crucial for assessing the robustness and accuracy of AI models in predicting basketball game outcomes. The reviewed studies widely used cross-validation techniques, particularly k-fold cross-validation, to ensure comprehensive model assessment. This method, which involves rotating dataset subsets as validation sets, provides a more reliable performance estimate than traditional Train-Test splits [41]. For example, Sikka et al. (2022) demonstrated that employing 5-fold and 10-fold cross-validation to evaluate their voting ensemble model, which integrated five base algorithms, achieved a high prediction accuracy, validating the robustness of cross-validation in minimizing overfitting and ensuring reliable performance estimation for basketball win percentage prediction [56].

Incorporating real-time data into model validation further enhances accuracy by accounting for the dynamic nature of basketball games. It was showed that dynamically updating models with current game data improved prediction accuracy [64], while static models relying solely on historical data often failed to adapt to ongoing game nuances [62]. Performance metrics such as accuracy, precision, recall, F1 score, Mean Absolute Error (MAE), RMSE, and Area Under the Curve of Receiver Operating Characteristic (AUC-ROC) were commonly used to evaluate AI models. The choice of metric varied depending on the prediction task. For instance, precision, recall, and the F1 score were emphasized for predicting rare events like upsets [9]. RMSE was frequently used to assess the precision of continuous predictions like total game scores or point differentials [40]. Selecting appropriate performance metrics that align with the prediction task’s goals is essential. While AUC-ROC is helpful for binary classification [7], it may be less effective for continuous outcomes or multi-class predictions.

In conclusion, robust and context-specific validation methods and performance metrics are necessary to develop accurate and applicable AI prediction models for basketball game predictions. Cross-validation combined with real-time data integration provides a reliable evaluation of model robustness. Future research should refine validation techniques and explore more sophisticated metrics to capture the complexities of basketball predictions.

### Static vs. dynamic predictions and comparative league analysis

Static prediction models rely on historical data and pre-game statistics, offering considerable accuracy in forecasts but lacking the ability to adapt to in-game changes like injuries or strategy shifts, leading to decreased accuracy as the game progresses [41,47,51].

In contrast, dynamic prediction models adjust predictions in real time, using updated data such as player fatigue and tactical changes to enhance accuracy. For example, it was showed that dynamic models could improve prediction accuracy from 62% at the start to 78% by the final quarter [64]. However, these models are less studied due to challenges in real-time data acquisition and computational efficiency [46]. Additionally, real-time predictive models must be capable of quickly integrating new information, such as sudden injuries, fouls, or changes in team strategy, which can significantly alter the course of a game. Delays in data processing and model inference can be a critical bottleneck, especially in high-stakes scenarios like live betting or in-game coaching decisions.

Another key observation in this review is the heavy concentration of existing research on the NBA, with 29 out of 34 studies focused specifically on this league. This NBA-centric focus raises concerns about the generalizability of AI models to other leagues that feature different playing styles, structures, and levels of competition, such as CBA [3] or the defense-oriented European leagues [62]. For instance, defensive metrics may play a more significant role in European leagues [77], while offensive efficiency metrics might be more critical in the NBA [78]. Additionally, the structure of the league, including season length, playoff format, and level of competition, can also impact model performance. Consequently, AI models trained on NBA data may not perform as effectively when applied to data from other leagues without significant adjustments. Future research should broaden its scope to include a wider range of leagues and game formats, focusing on developing adaptive AI models that can be fine-tuned or retrained to account for these inter-league differences. For example, incorporating transfer learning techniques, where a model trained on data from one league is adapted to another, could enhance the versatility of AI models and their applicability across global leagues [79,80].

### Identification of key features and prediction outcome types

Identifying relevant game features is crucial for improving prediction accuracy in basketball outcomes. Key metrics such as shooting efficiency (field goal percentage, three-point percentage), turnovers, and defensive rebounds significantly impact game results [9,40,52]. For instance, defensive rebounds are critical as they limit opponents’ scoring opportunities and enhance a team’s control over the game [7]. Field goal percentage is another crucial predictor, reflecting a team’s offensive efficiency. Advanced metrics like effective field goal percentage and offensive efficiency ratings provide a deeper understanding of scoring capabilities [62]. Additionally, rating systems like Elo ratings, PageRank, and player impact ratings improve predictive accuracy by dynamically capturing team and player strengths [62]. Different prediction tasks cater to various stakeholders. Binary win/loss predictions are relevant for casual viewers and bettors, while predictions involving scores and point differentials offer deeper insights valuable for strategic decisions by coaches and managers. The accuracy of these predictions depends on data quality, feature selection, and analysis techniques.

Integrating critical features into AI models is essential for improving their performance. Combining metrics like defensive rebounds, turnovers, and shooting efficiency enhances a model’s predictive power by offering a comprehensive view of team performance [7]. Ongoing feature selection and engineering refinement will continue to develop more accurate AI models for basketball predictions.

### Comparison with previous research studies

Several reviews have examined AI applications in predicting basketball outcomes. For example, a review highlighted high-performing models like LR and Hybrid Fuzzy-SVM, with 93.20% and 88.26% accuracy, respectively [42]. Our review, however, emphasizes the superior effectiveness of advanced models like MLP and RF, achieving accuracies of 98.90% and 93.81%.

Another review focused on AI in sports betting, noting the versatility of models [81]. Our findings align but extend this discussion, highlighting the superior accuracy of hybrid and ensemble techniques, particularly in complex tasks like real-time game outcome forecasting. Similarly, while the review discussed ensemble methods achieving 84% accuracy [43], our study shows that models using real-time data and dynamic updates, such as LSTM networks and GCNs, offer even higher predictive accuracy.

Building on a previous review that examined ML in various sports [82], our study focuses on basketball, underscoring the importance of feature engineering and real-time data integration. Ensemble techniques, like combining RF with GCNs, further advance AI methodologies in basketball analytics [7].

### Insight from traffic flow prediction for basketball game forecast

In the realm of basketball game outcome prediction, the dynamic and complex nature of the data poses significant challenges. Drawing on the dynamic analysis methods used in traffic flow forecasting can offer valuable insights for predicting basketball game outcomes.

Firstly, when exploring the “spatiotemporal” correlation of traffic flow scheduling, traffic flow prediction mainly focuses on the relationship of flow changes at different times and on different road sections [83,84]. In basketball games, the schedule can be analogized to “time”, and different opponents and game locations can be analogized to “space”. In the future, by analyzing the performance of teams at different stages of the season (such as the start, middle, and end of the season), against different opponents, and in home and away games, a model similar to the dynamic spatiotemporal connection in traffic flow can be constructed to capture the patterns of team form changes.

Secondly, in terms of dynamic weight allocation, in traffic flow prediction, the Spatio-Temporal Attention Unit controls the neighbor aggregation weights and integrates the spatiotemporal characteristics of different neighbors [83]. In the future, in basketball game prediction, learning can be enhanced by assigning dynamic weights to different game data. For example, at the critical moments of a game, the weights of scoring and rebounding data increase; at the beginning of a game, the weight of the historical head-to-head records of the two teams is greater. By dynamically adjusting the weights, the model can more reasonably integrate various data from different stages of the game, thus improving the prediction accuracy.

Finally, in terms of capturing the impact of unexpected events, traffic flow can be affected by unexpected incidents such as traffic accidents. Similarly, in basketball games, there are comparable situations, such as key players getting injured or sudden technical fouls. In the future, a similar mechanism can be established to promptly capture these “unexpected events”. Just as traffic flow prediction takes into account the impact of abnormal factors on traffic flow, we can assess their impact on the game trend and incorporate them into the prediction model. This will make the prediction results more consistent with the actual dynamic changes in the game.

### Limitations and directions for future research

This review highlights the use of AI techniques in predicting professional basketball game outcomes over the past five years. However, several recurring limitations should be noted across the included studies. Most research has focused primarily on the NBA, which limits the generalizability of the findings to other leagues such as the CBA, EuroLeague, and Turkish Basketball Super League. Moreover, there is a noticeable gap in research on women’s professional basketball games and 3x3 Basketball, which have unique dynamics and strategies that differ from traditional men’s 5-on-5 games. The lack of attention to these areas restricts the applicability of AI models across the broader spectrum of basketball competitions.

Methodologically, many studies relied on data from a single season [2,3,40,64], which may compromise the stability and universality of the results. Moreover, some studies did not adequately address feature engineering—failing to implement or report systematic methods for feature extraction and selection, such as correlation-based analysis, statistical selection techniques, or regularization methods [2,10,20,47,53,63]. Effective feature engineering is essential to identify the most influential variables, eliminate redundancies, and improve model interpretability and generalizability. Furthermore, while most studies focused on structured game statistics, few considered unstructured or contextual data such as player injuries, coaching decisions, or game location [9,22,47,50,51,60], which can significantly affect match outcomes.

Another significant limitation is the tendency of AI models, especially those utilizing DL techniques, to overfit the training data. While these models may perform exceptionally well on the data they were trained on, their performance often diminishes when applied to new, unseen data. This overfitting issue highlights the challenge of ensuring that AI models generalize well across different datasets and real-world scenarios. Additionally, many reviewed studies focus on pre-game predictions, with fewer efforts dedicated to real-time, in-game predictions. Real-time predictions are crucial for applications such as live betting and in-game strategy adjustments, yet they pose challenges regarding data acquisition, processing speed, and model adaptation. Developing models that can integrate and process real-time data effectively remains crucial for future research.

This review itself also has certain limitations that should be acknowledged. The included studies were limited to those published in English and retrieved from a select group of academic databases, which may have excluded relevant research published in other languages or sources. Moreover, the review emphasizes performance metrics such as accuracy, while paying less attention to the interpretability, transparency, and real-world applicability of AI systems.

Future research should expand data sources to include a broader range of leagues and international competitions, enhancing AI models’ generalizability. Incorporating data from women’s basketball leagues and 3x3 basketball competitions will provide additional insights and help create more universally applicable models. Furthermore, advanced validation techniques, such as nested cross-validation and time-series cross-validation, should be employed to improve model robustness and reduce the risk of overfitting. Exploring transfer learning approaches is another promising direction, as it allows models trained in one league to be adapted for use in another, enhancing their flexibility and applicability. Integrating AI with emerging technologies like the Internet of Things for real-time data collection could improve data quality and model performance, enabling more accurate and timely predictions. Improving feature engineering by developing techniques that capture the complexities of basketball games and combining domain knowledge with ML can lead to discovering novel and impactful features. Finally, longitudinal studies exploring the long-term impact of AI predictions over multiple seasons are needed to comprehensively understand their influence on team performance, player development, and league dynamics. Such studies could provide valuable insights into AI models’ sustainability and long-term effectiveness in professional sports.

## Conclusion

This study systematically evaluated AI technologies in predicting professional basketball outcomes over the past five years, highlighting the superiority of models like MLP, RF, and ensemble learning in enhancing predictive accuracy. The effectiveness of these AI models is largely attributed to advanced data processing, feature selection, and model optimization techniques, which outperform traditional methods. While dynamic models show promise for real-time decision-making, the current research is limited by its focus on NBA data, with insufficient exploration of other leagues and game types. Future research should broaden its scope to include diverse basketball contexts and utilize advanced validation methods and real-time data integration to improve prediction accuracy and model applicability.

## Supporting information

S1 File Search strategy. (DOCX)

S2 File Fig related data. (DOCX)

S3 File PRISMA 2020 Checklist. (DOCX)

S1 Table Basic information of included studies. (DOCX)

S2 Table Feature information, analysis and predicted outcome of included studies. (DOCX)

S3 Table Performance metrics and key findings of included studies. (DOCX)

S4 Table Study quality assessment. (DOCX)

S5 Table Studies exclude or include reasons. (DOCX)
