# Supplementary material for: The application of artificial intelligence techniques in predicting game outcomes of professional basketball league: A systematic review
Source: PLoS One. 2025 Jun 26;20(6):e0326326. doi: 10.1371/journal.pone.0326326 (PMC12200876; doi:10.1371/journal.pone.0326326)
Supplement: S1 File — (DOCX) [file pone.0326326.s001.docx]

**S1 Appendix. Search Strategy**

The following is a detailed summary of the search strategy for each database:

**PubMed:** The search string is (("artificial intelligence"[MeSH] OR "artificial intelligence"[TIAB] OR "AI"[TIAELMB] OR "machine learning"[TIAB] OR "deep learning"[TIAB] OR “neural network”[TIAB] OR "neural networks"[TIAB] OR "computational intelligence"[TIAB] OR “reinforcement learning”[TIAB] OR “computer reasoning”[TIAB] OR “machine intelligence”[TIAB] OR "intelligent systems"[TIAB]) AND ("basketball"[MeSH] OR "basketball"[All Fields]) AND English[lang])

**Web of Science:** The search string is ((“artificial intelligence” OR “computational intelligence” OR “AI” OR “machine intelligence” OR “computer reasoning” OR “machine learning” OR “deep learning” OR “neural network” OR “neural networks” OR “reinforcement learning” OR “intelligent systems”) AND ("basketball"))

**Scopus:** The search string is ((“artificial intelligence” OR “computational intelligence” OR “AI” OR “machine intelligence” OR “computer reasoning” OR “machine learning” OR “deep learning” OR “neural network” OR “neural networks” OR “reinforcement learning” OR “intelligent systems”) AND ("basketball"))

**EBSCO:** The search string is (“artificial intelligence” OR “computational intelligence” OR “AI” OR “machine intelligence” OR “computer reasoning” OR “machine learning” OR “deep learning” OR “neural network” OR “neural networks” OR “reinforcement learning” OR “intelligent systems”) AND ("basketball")
