# Supplementary material for: The application of artificial intelligence techniques in predicting game outcomes of professional basketball league: A systematic review
Source: PLoS One. 2025 Jun 26;20(6):e0326326. doi: 10.1371/journal.pone.0326326 (PMC12200876; doi:10.1371/journal.pone.0326326)
Supplement: S2 File — (DOCX) [file pone.0326326.s002.docx]

Fig4 Relationship between input Features and Prediction Accuracy

| Features | Accuracy |
| --- | --- |
| 17 | 58.9% |
| 12 | 84% |
| 2 | 60.65% |
| 21 | 83% |
| 12 | 75% |
| 26 | 60.01% |
| 16 | 87.5% |
| 14 | 67.24% |
| 21 | 93.81% |
| 9 | 79.2% |
| 10 | 98.90% |
| 14 | 61% |
| 15 | 76.52% |
| over 50 features | 69.88% |
| 61 | 67.98% |
| 30 | 65.8% |
| 42 | 78% |
| 110 | 78% |
| 17 | 92% |
| 12 | 83.78% |
| 44 | 71.54% |
| 32 | 78.2% |

Fig5 Comparison of Different Al Models' Accuracy

| Model | Accuracy |
| --- | --- |
| Bayesian Network | 58.9% |
| Hybrid Ensemble Learning | 84% |
| Naive ML Algorithm | 60.65% |
| LMT | 83% |
| AdaBoost model | 75% |
| KNN | 60.01% |
| Regression tree | 87.5% |
| LASSO | 67.24% |
| RF | 93.81% |
| CNFS | 79.2% |
| MLP | 98.90% |
| Fuzzy Theory | 61% |
| ANN | 68.08% |
| GA-ANN | 76.52% |
| LR | 78.2% |
| Data-Driven ML Algorithm | 78% |
| Ensemble Learning | 78% |
| Linear Regression | 92% |
| GCN + RF | 71.54% |
| NN | 78.2% |
