# Supplementary material for: The application of artificial intelligence techniques in predicting game outcomes of professional basketball league: A systematic review
Source: PLoS One. 2025 Jun 26;20(6):e0326326. doi: 10.1371/journal.pone.0326326 (PMC12200876; doi:10.1371/journal.pone.0326326)
Supplement: S1 Table — (DOCX) [file pone.0326326.s004.docx]

**Table 1 Basic information of included studies**

| **Study ID** | **First Author, Year** | **Country** | **AI Techniques Used** | **Datasets source** | **Number of seasons** | **Types of seasons** | **Number of teams** | **Amount of Data** | **Model Validation** |
| --- | --- | --- | --- | --- | --- | --- | --- | --- | --- |
| 1 | Alameda-Basora et al. 2019 [4] | USA | Expert  Bayesian Network | NBA 2013/2014-  2018/2019 | 6 | Regular | 30 teams | 17661 instances | ***Data segmentation:***  Training set: 17,361 instances (5,787 games per quarter) 2013/2014-2017/2018 seasons  Testing set: 300 instances (100 games per quarter) 2018-2019 seasons |
| 2 | Cai et al.  2019 [3] | China | Hybrid Ensemble Learning | CBA  2016/2017 | 1 | Regular | 20 teams | 280 games | ***Data segmentation:***  Training set: 210 games  Testing set: 70 games |
| 3 | Horvat et al. 2019 [20] | Croatia | Naive ML Algorithm | NBA 2009/2010-  2017/2018 | 9 | Regular | 30 teams | 11578 games | ***Data segmentation:***  Training set: Various combinations of training seasons (from one to multiple seasons)  Evaluation set: Various combinations of evaluation seasons (from one to multiple seasons) |
| 4 | Kayhan et al. 2019 [46] | USA | Data Snapshot Approach, LSTM, GLM | NBA  2009-2017 | 7 | Regular | 30 teams | 8329 games | ***Data segmentation:***  Training set: 7099 games 2009-2016 seasons  Validation set: 1230 games 2016-2017 season  ***Cross-validation*** |
| 5 | Lu et al.  2019 [47] | China | Ordinary Least Squares, Weighted Linear Regression | NBA 2012/2013-  2016/2017 | 5 | Regular and playoffs | 30 teams | All games from the 2012 to 2016 | Out-of-sample validation |
| 6 | Thabtah et al.  2019 [9] | New Zealand | NB, ANN, DT(J48), Logistic Model Tree | NBA  1980-2017 | 38 | Regular and finals | 11 teams (participated in the finals between 1980 and 2017) | 430 instances | ***Cross-validation:***  10-fold cross-validation |
| 7 | Yao,  2019 [48] | USA | MLR, NN | NBA  1992-2019 | 27 | Regular | 30 teams | Data from 27 seasons | ***Data segmentation:***  Training set: 1992-2018 seasons  Testing set: 2018-2019 seasons  ***Cross-validation:***  5-fold cross-validation |
| 8 | Giasemidis, 2020 [49] | UK | LR, SVM with linear and RBF Kernels, DT, RF, NB, GB, KNN, Discriminant Analysis, AdaBoost | EuroLeague  2016/2017-  2018/2019 | 3 | Regular | 16 teams per season | Each team played 30 games per season | ***Data segmentation:***  Training set: 2016/2017-2017/2018 seasons  Testing set: 2018-2019 season  ***Cross-validation:***  5-fold cross-validation |
| 9 | Horvat et al. 2020 [41] | Croatia | LR, NB, DT, MLP, RF, KNN, LogitBoost | NBA 2009/2010-  2017/2018 | 9 | Regular and playoffs | 30 teams | 11578 games | ***Data segmentation:***  Train & Test validation method  ***Cross-validation:***  5-fold cross-validation |
| 10 | Huang et al. 2020 [50] | Taiwan, China | RT(M5P),  Linear regression, Support vector regression | NBA  2017/2018 | 1 | Regular | 30 teams | 82 games (Data from Golden State Warriors and opponents) | ***Data segmentation:***  Training set: 60% of the data  Validation set: 20% of the data  Testing set: 20% of the data |
| 11 | Li,  2020 [51] | China | Linear Regression, LR, SVM | NBA 2012/2013- 2017/2018 | 6 | Regular | 30 teams | 7380 games | ***Data segmentation:***  Training set: 2012-2014 seasons  Testing set: 2015, 2016, 2017 seasons |
| 12 | Migliorati, 2020 [52] | Italy | CART, RF | NBA 2004/2005-  2017/2018 | 14 | Regular | 30 teams, with a focus on GSW | 17000 games approximately | ***Data segmentation*:**  Training set: 90% of GSW games (1,017 games)  Testing set: 10% of GSW games (113 games) |
| 13 | Ozkan,  2020 [5] | Turkey | ANN, CNFS | Turkish Basketball Super League 2015-2016 season | 1 | Regular | 16 teams | 240 games | ***Data segmentation:***  Training set: 216 games  Testing set: 24 games |
| 14 | Song et al. 2020 [1] | China | Gamma process model (Probabilistic Modeling), Hybrid Adjustment with Betting Line | NBA 2015/2016-  2017/2018 | 3 | Regular | 30 teams | NA | ***Data segmentation:***  Training set: First half of games in the regular season  Testing set: Second half of games in the regular season |
| 15 | Ballı et al. 2021 [53] | Turkey | KNN, LR, MLP, NB, DT(J48), Voting | EuroLeague  2012/2013-  2016/2017 | 5 | Regular and playoffs | All EuroLeague teams | Dataset 1: 1266 games  Dataset 2: 720 games  Dataset 3: 259 games  Dataset 4: 509 games  Dataset 5: 760 games | ***Data segmentation:***  Training set: Various, including 66%, 80%  ***Cross-validation:***  10-fold cross-validation |
| 16 | Chen et al. 2021 [40] | Taiwan, China | ELM, MARS, KNN, XGBoost, SGB | NBA  2018-2019 | 1 | Regular | 30 teams | 2460 game data points | ***Cross-validation:***  10-fold cross-validation |
| 17 | Lu et al.  2021 [2] | Taiwan, China | CART, RF, SGB, XGBoost, ELM | NBA  2018-2019 | 1 | Regular | 30 teams | 1230 games | ***Cross-validation:***  10-fold cross-validation |
| 18 | Chen et al. 2022 [21] | China | Fuzzy Theory | NBA  2018-2019 | 1 | Regular | 30 teams | 800 games | Experimental validation |
| 19 | Khanmohammadi et al. 2022 [54] | USA | Hybrid neural network:  Feature Imitating Networks, Convolutional neural networks, LSTM, Dense Layers | NBA 2017/2018-  2021/2022 Iranian Super League playoffs 2020-2021 | 5 for NBA, 1 for Iranian Super League playoffs | Regular and playoffs | 30 NBA teams, multiple teams from the Iranian Super League | Each year’s season games as training data (1,230, 1,120, 1,060, 1,086, and 1,236 games from 2017/2018-2021/2022) and playoff games as testing data (82, 82, 83, 85, and 87 games from 2017/2018-2021/2022) | ***Data segmentation:***  Training set: NBA regular season games  Testing set: NBA playoff games and Iranian Super League playoff games |
| 20 | Krishnan et al.  2022 [55] | India | LR, ANN | NBA 2010/2011-  2020/2021 | 10 | Regular and playoffs | 30 teams | Data from 82 games per season, except for the 2019-2020 season (72 games) and 2011-2012 season (66 games) | ***Data segmentation:***  Training set: 2010-2019 seasons  Testing set: 2019-2021 seasons |
| 21 | Ma et al.  2022 [22] | China | Linear Regression, XGBoost, NN | NBA  2013-2018 | 5 | Regular | 30 teams | 89406 instances | ***Cross-validation:***  5-fold cross-validation |
| 22 | Osken et al. 2022 [23] | Turkey | ANN, GA, Clustering Techniques: K-Means and C-Means clustering | NBA 2012/2013-  2017/2018 | 6 | Regular | 30 teams | 6150 games | ***Data segmentation:***  Training set (75%): 2012/2013- 2016/2017 seasons  Testing set (25%):  2017-2018 season |
| 23 | Sikka et al. 2022 [56] | India | Voting Ensembling Regression (Multiple Linear Regression, DT, RF, GB, Gaussian Process Regression) | NBA 2016/2017-  2020/2021 | 5 | Regular | 30 teams | NA | ***Cross-validation:***  5-fold and 10-fold cross-validation in various models |
| 24 | Su et al. 2022 [57] | China | XGBoost, RF, BPNN, GRNN | NBA 2011/2012-  2017/2018 | 7 | Regular | 30 teams | 2700 player-season records | ***Data segmentation:***  Training set: 90% of the data (2430 data)  Testing set: 10% of the data (270 data)  ***Cross-validation:***  10-fold cross-validation |
| 25 | Santos et al. 2022 [58] | Sweden | LR, Linear SVM, RF, MLP | NBA 2008/2009-2017/2018 | 10 | Regular and playoffs | 30 teams | 12,300 regular games | ***Data segmentation:***  Training set: 2008/2009-2016/2017 seasons  Testing set: 2017-2018 season  10-fold cross-validation |
| 26 | Wang et al. 2022 [59] | UK | LIME, RF, FNN | NBA  1980-2019 | 40 | Regular | 30 teams | Win ratio prediction: 4718 data points; post-season playoffs classification: 1055 data points | ***Data segmentation:***  Training set: 90% of the data  Testing set: 10% of the data  ***Cross-validation:***  leave 10 out cross validation, 11-Fold Cross-Validation |
| 27 | Zheng, 2022 [60] | China | RF, NB, SVM, LR, FNN | NBA 2012/2013-  2021/2022 | 10 | Regular | 30 teams | 10364 instances | ***Data segmentation:***  Training set (90%): 9161 games from 2012/2013-2020/2021 seasons  Testing set (10%): 1036 games from 2020/2021 -2021/2022 seasons  ***Cross-validation:***  10-fold cross-validation |
| 28 | Daundkar et al. 2023 [61] | India | NB, KNN, RF, DT, LR, SVM | NBA  2018-2019 | 2 | Regular | 30 teams | NBA 2018: 1230 games; NBA 2019: 1080 games | ***Cross-validation:***  5-fold cross-validation |
| 29 | Horvat et al. 2023 [6] | Croatia | LR, NB, DT, MLP, KNN, RF, LogitBoost | NBA 2013/2014-  2017/2018 | 5 | Regular | 30 teams | 6567 games | ***Data segmentation:***  Training set: up to 4 seasons  Testing set: up to 2 seasons |
| 30 | Lampis et al.  2023 [62] | Greece | Ensemble Learning, LR, RF, XGBoost | Four different European tournaments (EuroLeague, Eurocup, Greek Basket League, and Spanish Liga ACB) 2013-2018 | 5 | Regular and play-offs | All teams | 5214 games | ***Data segmentation:***  Training set: 2014/2015-2016/2017 seasons  Testing set: 2017/2018 season  ***Cross-validation:***  10-fold cross-validation |
| 31 | Patrot et al. 2023 [24] | India | Linear Regression, SVM, DT | NBA seasons | NA | Regular | All NBA teams | NA | ***Data segmentation:***  Training set: 90%  Testing set: 10%  Training set: 80%  Testing set: 20% |
| 32 | Wang,  2023 [63] | USA | LR, SVM, RF, DNN, RNN (LSTM) | NBA 2004/2005-  2020/2021 | 16 | Regular | 30 teams | 25797 games | ***Data segmentation:***  Training set: 2004/2005-2019/2020 seasons  Testing set: 2020-2021 season  ***Cross-validation:***  5-fold cross-validation |
| 33 | Zhao et al. 2023 [7] | China | GCN, RF | NBA 2012/2013-  2017/2018 | 6 | Regular | 30 teams | 7379 games | ***Data segmentation:***  Training set: 70% of the data  Validation set: 10% of the data  Testing set: 20% of the data  ***Cross-validation:***  10-fold cross-validation |
| 34 | Kandhway, 2024 [64] | India | RF, NN, Support Vector Classifier, LR | NBA 2022/2023 | 1 | Regular | 30 teams | 1236 games | ***Data segmentation:***  Training set: 605 games  Validation set: 260 games  Testing set: 371 games |

NBA: National Basketball Association, CBA: Chinese Basketball Association, ML: machine learning, LSTM: Long Short-Term Memory, GLM: general linear model, NB: naive bayes, ANN: artificial neural networks, DT: decision tree, MLR: multiple linear regression, NN: neural network, LR: logistic regression, SVM: support vector machine, RBF kernel: radial basis function kernel, RF: random forest, NB: naive bayes, GB: gradient boosting, MLP: multilayer perceptron, KNN: k-nearest neighbors, CART: classification and regression trees, CNFS: Concurrent Neuro Fuzzy System, ELM: extreme learning machine, MARS: Multivariate Adaptive Regression Splines, XGBoost: eXtreme Gradient Boosting, SGB: Stochastic Gradient Boosting, RNN: recurrent neural network, GA: genetic algorithms, BPNN: backward neural network, GRNN: generalized regression neural network, LIME: Local Interpretable Model-agnostic Explanations, FFNN: feed forward neural network, GCN: graph convolutional network.
