# Supplementary material for: The application of artificial intelligence techniques in predicting game outcomes of professional basketball league: A systematic review
Source: PLoS One. 2025 Jun 26;20(6):e0326326. doi: 10.1371/journal.pone.0326326 (PMC12200876; doi:10.1371/journal.pone.0326326)
Supplement: S2 Table — (DOCX) [file pone.0326326.s005.docx]

**Table 2 Feature information, analysis and predicted outcome of included studies**

| **Study ID** | **First Author, Year** | **Feature Selection Methods** | **Feature Extraction methods** | **Types of Input Features** | **Number of Input Features** | **The predicted outcome** |
| --- | --- | --- | --- | --- | --- | --- |
| 1 | Alameda-Basora et al. 2019 [4] | Information gain ratio, Chi-square statistic; Domain knowledge for removing redundant features | Discretization of continuous features by examining minimum and maximum values and determining bin intervals using domain knowledge | ***Team statistics:***  MP, shots made by H/A teams, 3FGM, ORtg, DRtg, shooting efficiency, total points scored by both teams at the end of each quarter, etc. | 17 features | Total points (Over or Under bet) |
| 2 | Cai et al.  2019 [3] | Bagging Strategy and Random Subspace Method | Weighted combination of features from recent games, Random Subspace Method | ***Team statistics:***  two-point shots, three-point shots, free-throws, attack, defense, fouls, fast breaks, dunks, AST, STL, TOV, BLK | 12 characteristics | Game winner (win or loss) |
| 3 | Horvat et al. 2019 [20] | NA | Win/loss ratio extraction (all games or mutual games) | ***Team statistics:***  win/loss ratios of H/A teams | 2 features | Game winner (win or loss) |
| 4 | Kayhan et al. 2019 [46] | Elastic Net regularization | Snapshot from current game, weighted average of end-of-game point spreads, cumulative in-game statistics | ***Team statistics:***  Point difference:  point spread at each minute of the game  Cumulative in-game statistics (e.g., FT%, FG%, etc.) | Data Snapshot: 2 (time, point spread);  LSTM: 48 time steps (1D sequence)  GLM: 16 | End-of-game point spread |
| 5 | Lu et al.  2019 [47] | NA | NA | ***Team statistics:***  previous game results, team ability, home advantage | 2 features | Point differences between the H/A teams |
| 6 | Thabtah et al.  2019 [9] | Multiple Regression, Correlation Feature Set, and RIPPER algorithm | NA | ***Team statistics:***  H/A status, FGA, 3FGA, MP, FTA, FTM, ORB, etc.  ***Player statistics:***  opponent statistics | 21 features | Game winner (win or loss) |
| 7 | Yao,  2019 [48] | Backward Elimination, Correlation Matrix Analysis | NA | ***Team statistics:***  offensive, defensive, and differential statistics (e.g., FG %, 3P%, FT%, number of disqualified players, three-second violations, flagrant fouls, technical fouls, double doubles, and triple doubles, etc.) | Model-1: 14 parameters  Model-2: 64 parameters | Winning percentages of NBA teams |
| 8 | Giasemidis, 2020 [49] | ANOVA F-test, Mutual Information, Chi-Square Test, Wrapper Methods | NA | ***Team statistics:***   1. Game-level results: round number, date, H/A teams, PTS, etc. 2. season standings: total points, wins and losses, total offense and defense, score difference, etc. 3. position in table, form (past five games won), final-four flag | 12 features | Game winner (win or loss) |
| 9 | Horvat et al. 2020 [41] | NA | NA | ***Team statistics:***  3FGM, 3FGA, 2FGM, 2FGA, FTA, FTM, DRB, ORB, AST, STL, TOV, BLK, fouls, etc. | 26 features | Game winner (win or loss) |
| 10 | Huang et al. 2020 [50] | Chi-Square Test | NA | ***Player statistics:***  FGA, FG%, 3FGA, 3P%, FTA, FT%, ORB, DRB, AST, STL, BLK, TOV, PF, PTS, MP, TRB | 16 features | Game scores for individual players and the total team score |
| 11 | Li,  2020 [51] | LASSO, Correlation Matrix Analysis | Sliding Window Averages, composite statistics | ***Team statistics:***  team points, FGA, ORB, TOV, FTA, ORtg, DRtg, etc. | 14 features | Game winner (win or loss) |
| 12 | Migliorati, 2020 [52] | Gradually eliminate irrelevant or secondary variables | Basic statistics based on box scores are used and more advanced statistical features are extracted by calculating four key factors | ***Team statistics:***   1. Box scores: PTS, 2FGA, 2FGM, 2FGA, 2FGM, FTA, FTM, ORB, DRB, AST, TOV, STL, BLK, etc. 2. Dean’s Four Factors: eFG% for the team and the opponent, turnover ratio for the team and the opponent, offensive and defensive rebounding percentage for the team and the opponent, free throws rate for the team and the opponent | 21 features (13 box score statistics, 8 four factors) | Game winner (Win or loss) for Golden State Warriors in NBA games |
| 13 | Ozkan,  2020 [5] | Correlation Analysis, Normalization | PCA, Fuzzy Logic Inputs | ***Team statistics:***  code of the H/A team, average points of the H/A team in the last four games, average points of the H/A team in the league, average points of the competitors of H/A team in the last four games, week of the league | 9 features | Game winner (win or loss) |
| 14 | Song et al. 2020 [1] | Historical Data Integration | Gamma Process Modeling | ***Team statistics:***  Total points scored  Bookmaker’s betting lines | 2 features | Total points (Over or Under bet) |
| 15 | Ballı et al. 2021 [53] | NA | Hybrid Models: Combined Four Factors (8 features) and DefenseOfense (16 features) into four hybrid models | ***Team statistics:***   1. Offensive Statistics: FGM, FGA, FTM, FTA, ORB, AST, TOV, etc. 2. Defensive statistics: DRB, BLK, STL, fouls, etc. 3. Hybrid features: FG%, TOV%, ORB%, FT%, etc.   Derived Features:   1. Four Factors Model: ORtg, DRtg, TOV%, ORB%, FT%, etc. 2. DefenseOfense Model: H/A team’s offensive and defensive indexes, combining multiple statistics into composite measures, etc. | 16 features (DefenseOfense Detailed Model), 8 features (Four Factors Detailed Model), 10 features (Hybrid Models, Model 6), 24 features (Hybrid Models, Model 8) | Game winner (win or loss) |
| 16 | Chen et al. 2021 [40] | MARS, XGBoost, SGB | Generating features based on game-lag information | ***Team statistics:***  2FGA, 2FG%, 3FGA, 3FG%, FTA, FT%, ORB, DRB, AST, STL, BLK, TOV, PF | 13 features | Final scores |
| 17 | Lu et al.  2021 [2] | NA | Adaptive weighted feature design | ***Team statistics:***  2P%, 2FGM, 3P%, 3FGM, FTA, FT%, ORB, DRB, AST, STL, BLK, TOV, PF, H/A game | 14 features | Final scores |
| 18 | Chen et al. 2022 [21] | Fuzzy Theory analysis | Calculating average values of game parameters over a period of time | ***Team statistics:***  average PPG, FG%, 3P %, AST, FTA, DRB, TRB, TOV, PF, STL, BLK, points in the paint, team stats, recent meetings | 14 features | Game winner (win or loss) |
| 19 | Khanmohammadi et al. 2022 [54] | Feature Imitating Networks | Feature Imitating Networks, LSTM, Convolutional neural networks | ***Team statistics:***  FG%, ORB, DRB, AST, etc.  ***Player statistics:***  PTS, MP, FGA, etc. | Team statistics: 35 features; Player statistics: 34 features | Playoff game winner (win or loss) |
| 20 | Krishnan et al.  2022 [55] | Correlation Analysis | One-Hot Encoding, Heatmap Correlation Analysis | ***Team statistics:***  the number of 3FGM by a team, the average pace of a team  ***Player statistics:***  PPG, AST, rebounds, player efficiency ratings, etc. | NA | Win-loss percentage |
| 21 | Ma et al.  2022 [22] | Pearson Correlation Coefficient, weighted averaging | Data Normalization | ***Player statistics:***  total points, three-point shots, TRB, AST, STL, BLK, TOV, double-doubles, and triple-doubles  ***External factors:***  Team and Contextual Features: home-court advantage, rest days, team sheets, player positions, salary information, player value, etc. | 29 features | Game winner (win or loss) |
| 22 | Osken et al. 2022 [23] | Clustering Analysis (k-means and c-means) | PCA | ***Team statistics:***  team win percentage, the month of the season, rest days  ***Player statistics:***  PTS, rebounds, AST, true shooting percentage, eFG%, rebound rate, AST%, spatial distribution of shots | 15 components from PCA + additional team-level attributes (e.g., rest days, month of the season, winning percentage) | Game winner (win or loss) |
| 23 | Sikka et al. 2022 [56] | Correlation Heatmap Analysis | NA | ***Team statistics:***  PPG, points allowed per game, average points differential, offensive efficiency, defensive efficiency, efficiency differential, strength of schedule | 7 features | Team’s win percentage over the course of a season |
| 24 | Su et al. 2022 [57] | Correlation Analysis | Minimal explicit extraction; relied on existing NBA metrics (e.g., field goals, free throws). | ***Player statistic******s:***  games played, games started, field goals and attempts, three-point field goals and attempts, two-point field goals and attempts, free throws and attempts, TRB, ORB, DRB, AST, STL, BLK, TOV, PTS, FG%, MP, position, age, team affiliation, player salary | 25 features | Player scores (Points per game) |
| 25 | Santos et al. 2022 [58] | Gini impurity for RF, Hyperparameter tuning of the model | NA | ***Team statistics:***  H/A team, the stage of the season, number of games played in the regular season and playoffs, wins and losses in various contexts (e.g., last game, last home game, last 3/8/15 games), whether the game is back-to-back, previous season performance  ***Player statistics:***  the number of wins contributed by a player per 48 minutes (total time played in a game without overtime)  ***External factors:***  salary data (e.g., total salary, salary cap ratio), draft pick data (e.g., draft positions over previous years) | over 50 features | Game winner (win or loss), championship winners |
| 26 | Wang et al. 2022 [59] | Domain knowledge for removing redundant features, attribute aggregation | PCA, Clustering (K-means, DBSCAN) | ***Team statistics:***  FGM, 3P%, FT%, TOV, ORB, AST, PPG, etc. | NA | Monthly win ratio (game winning percentage), Post-season playoff qualification |
| 27 | Zheng, 2022 [60] | Sequential Forward Selection,  Recursive Feature Elimination | NA | ***Team statistics:***  rebounds, PTS, AST, TOV, STL, BLK, FT, 2FGA, 2FGM, 3FGA, 3FGM, etc.  Advanced statistics:  Elo ratings, tiredness levels, and home court advantage | 38 features in Feature Set A; 40 features in Feature Set B; 61 features in Feature Set C | Game winner (win or loss) |
| 28 | Daundkar et al. 2023 [61] | Chi-square Test, Gini Scores from RF | Features computed as averages of past box scores | ***Team statistics:***  PTS, FGA, 3FGA, 3FGM, FTA, FTM, ORB, DRB, AST, STL, BLK, PF, TOV, etc. | 30 features | Game winner (win or loss) |
| 29 | Horvat et al. 2023 [6] | Information Gain | NBA Team Efficiency Index: Combines box scores (points, rebounds, assists) into a team-level metric.  Extended Team Efficiency Index (combining player/team performance and opponent comparisons) | ***Team statistics:***  PTS, AST, BLK, ORB, DRB, STL, PF, TOV, total points scored, TRB, goals missed from different fields, eFG, true shooting success ratio, assist-to-points ratio, BLK per opponent FGA, offensive rating, win-loss records over the last 10 games, H/A win-loss records, winning streaks, the number of games played in the last 10 days  ***Player statistics:***  player efficiency index | 42 features (13 basic game elements, 8 derived game elements, 13 advanced game elements, 8 league-wide statistics) | Game winner (win or loss) |
| 30 | Lampis et al.  2023 [62] | LR with Regularization, RF, XGBoost | Creating new performance indicators from historical statistics, key performance indicators, and rating systems (Elo, PageRank, pi-rating) | ***Team statistics:***  1. Historical information features: Percentage of wins, ORtg, DRtg;  2. Rating systems: Elo rating, PageRank, pi-rating;  3. Current form: statistics from the last 10 games;  4. Tournament characteristics: specific statistics and features related to each tournament. | 110 features | Game winner (win or loss) |
| 31 | Patrot et al. 2023 [24] | Correlation Feature Set, Multiple Regression | NA | ***Team statistics:***  wins, losses, PPG, offensive rating, defensive rating, pace, FG%, 3P%, FT%, ORB, DRB, AST, STL, BLK, TOV, PF | 17 features | Game winner (win or loss) |
| 32 | Wang,  2023 [63] | Correlation-based filtering | NA | ***Team statistics:***  PTS, FG%, FT%, 3P%, AST, rebounds, home team wins, etc. | 12 features | Game winner (win or loss) |
| 33 | Zhao et al. 2023 [7] | LASSO, RF | PCA | ***Team statistics:***  rebounds, FGA, STL, AST, BLK, PF, TOV, FG%, 3P%, FT%, 2P%, ORtg, DRtg, true shooting percentage, etc. | 44 features | Game winner (home win or away win) |
| 34 | Kandhway, 2024 [64] | Manual selection based on domain expertise | Time-stamped match data converted to numerical features | ***Team statistics:***  PTS, field goals, three pointers, free throws, rebounds, STL, AST, BLK, PF, TOV, quarter identifier, win ratio difference of H/A teams, etc. | 32 features | Game winner (win or loss) |

MP: minutes played, H/A: home/away, 3FGM: three-pointers made, Ortg: offensive rating, DRtg: defensive rating, AST: assists, STL: steals, TOV: turnovers, BLK: blocks, NA: not applicable, FT: free throw, FG: field goal, LSTM: Long Short-Term Memory, GLM: general linear model, FGA: field goal attempts, 3FGA: three-pointers attempted, FTA: free throws attempted, FTM: free throws made, ORB: offensive rebounds, RIPPER: Repeated Incremental Pruning to Produce Error Reduction, 3P%: three-point field goal percentage, ANOVA: Analysis of Varianc, PTS: points, DRB: defensive rebounds, PF: personal fouls, TRB: total rebounds, 2FGA: two-pointers attempted, 2FGM: two-pointers made, LASSO: Least Absolute Shrinkage and Selection Operator, eFG%: effective field goal percentage, MARS: Multivariate Adaptive Regression Splines, XGBoost: eXtreme Gradient Boosting, SGB: Stochastic Gradient Boosting, PPG: points per game, ANN: artificial neural networks, PCA: Principal Component Analysis, RF: random forest, LR: logistic regression, Pi-rating: player impact ratings.
