# Supplementary material for: The application of artificial intelligence techniques in predicting game outcomes of professional basketball league: A systematic review
Source: PLoS One. 2025 Jun 26;20(6):e0326326. doi: 10.1371/journal.pone.0326326 (PMC12200876; doi:10.1371/journal.pone.0326326)
Supplement: S3 Table — (DOCX) [file pone.0326326.s006.docx]

**Table 3 Performance metrics and key findings of included studies**

| **Study ID** | **First Author, Year** | **Performance Metrics** | **Key Findings** |
| --- | --- | --- | --- |
| 1 | Alameda-Basora et al. 2019 [4] | **Accuracy:**   - Expert Bayesian Network: Overall accuracy of 58.9% - Non-Expert Bayesian Network: Overall accuracy of 44.8% | 1. The Expert Bayesian Network outperformed all other models and amateur betting strategies, achieving a profit margin of over 10% on live-betting. 2. The inclusion of domain knowledge in the Expert Bayesian Network significantly improved prediction accuracy compared to the Non-Expert Bayesian Network. 3. The Bayesian Network provided valuable live-betting predictions by dynamically updating probabilities based on in-game statistics as the game progressed. 4. Incorporating domain knowledge into the BN structure significantly improved prediction accuracy and profitability. |
| 2 | Cai et al.  2019 [3] | **Hybrid Ensemble Learning Framework:**  **Accuracy:** 84%  **F1-score:** 82% | 1. The hybrid ensemble framework (SVM + Bagging + RSM) demonstrated superior accuracy (84%), surpassing standalone models (e.g., NN: 71%, NB: 76%). 2. The integration of bagging and the RSM effectively addresses common issues such as feature redundancy, sample noise, and imbalanced datasets. These techniques work together to increase sample diversity and reduce feature dependency, thereby improving overall model performance. |
| 3 | Horvat et al. 2019 [20] | **Accuracy:**  *Using all played games during the training phase:*   - 2016 training period, 2017 evaluation: 58.84% (training) and 60.23% (including evaluation phase) - 2015 training period, 2016 -2017 evaluation: 59.25% (training) and 60.48% (including evaluation phase) - 2014 training period, 2015 -2017 evaluation: 59.41% (training) and 60.65% (including evaluation phase)   *Using only mutual games during the training phase:*  2014 training period, 2015-2017 evaluation: 58.90% (training) and 60.27% (including evaluation phase) | 1. Shorter training periods (1 season) outperformed multi-season datasets due to team roster/strategy dynamics, achieving peak accuracy (~60%). 2. Including evaluation phase games in training improved prediction accuracy, as recent data better reflected current team states. 3. Using all historical games (Method 1) outperformed focusing on mutual games (Method 2) by ~0.4%. 4. The study highlights dataset length trade-offs in sports analytics: While longer datasets risk outdated patterns, shorter spans capture transient team performance but limit generalizability. |
| 4 | Kayhan et al. 2019 [46] | **MAE:**   - Data snapshot approach, LSTM network, and GLM had nearly identical MAE values - Initial MAE: ~11 points at the beginning of games - Half-time MAE: slightly above 8 points - Final minute MAE: within 2 points | 1. The data snapshot approach performed similarly to the LSTM network and GLM in predicting the point spread, with nearly identical MAE values throughout the game. 2. All models showed high initial prediction errors (~11 points), which decreased as the game progressed, with final predictions being within 2 points of the actual spread. 3. The data snapshot approach offers significant computational efficiency, requiring no training time, unlike the resource-intensive LSTM and GLM models. 4. The point difference at a given time is identified as the single best predictor of the game's outcome, indirectly incorporating all in-game events and team dynamics. 5. The data snapshot approach can help bettors assess risks, bookmakers adjust lines in real time, and coaching staff make decisions about player substitutions, timeouts, or fouls. |
| 5 | Lu et al.  2019 [47] | **Standard deviation between the actual point difference and predicted point difference:**   - One-year prediction: 15.32 for playoff games, 12.43 for regular season games - Out-of-sample prediction: 12.44 - Weighted prediction: Best result with a standard deviation of 11.97555, slope 0.91, and p-value 0.06 | 1. The model successfully predicts the point difference in NBA games using a linear regression approach based on team ability and home advantage. 2. The model's standard deviation for predicting playoff game outcomes is 15.32, and for regular season games, it is 12.43, slightly outperforming bookie’s predictions. 3. Using out-of-sample predictions for validation, the model achieved a standard deviation of 12.44, indicating reliable performance. 4. Applying different weighting strategies to recent games improved prediction accuracy, with the best method achieving a standard deviation of 11.97555. 5. The model effectively estimated separate home advantages and team abilities over five years, demonstrating robustness in predictions across different seasons. 6. The model identified the Golden State Warriors as the strongest team in the 2016-17 season, accurately predicting the outcomes of 13 out of 15 playoff games. 7. The model does not account for injuries, trades, and coaching changes, which could affect its accuracy. |
| 6 | Thabtah et al.  2019 [9] | **Accuracy (Dataset D)**   - NB: 80% - ANN: 80% - LMT: 83%   **Precision, Recall, F1 Score from Dataset D:** All three metrics were highest for LMT at 83% each | 1. Combining LR and DT, LMT outperformed ANN/NB, achieving 83% accuracy with selected features. 2. Feature selection methods (multiple regression, correlation feature set, RIPPER algorithm) were applied, highlighting defensive rebounds as the most significant feature. Other important features included three-point percentage, free throws made, field goal percentage, and total rebounds. 3. Feature selection is crucial for improving model performance. The use of feature selection improved prediction accuracy by 2%-4%. |
| 7 | Yao,  2019 [48] | **RMSE:**   - Linear Regression Model: 0.047 - NN Model-1 (14 parameters): 0.043 - NN Model-2 (64 parameters): 0.035 - Ensemble learning (combined model): 0.034   **R²:**   - Ensemble learning (combined model): 0.954 | 1. NN models demonstrated better prediction accuracy compared to linear regression models due to their ability to handle non-linear parameters and implement ensemble learning. 2. The linear regression model identified critical variables such as field goal percentage and field goal percentage allowed as the most significant predictors of winning percentage. 3. NN Model-1, which used the same 14 parameters as the linear regression model, showed an 8% lower RMSE, indicating better fitting. NN Model-2, which utilized 64 parameters, including unique variables like disqualifications and technical fouls, further reduced RMSE by 20%, demonstrating the advantages of using a larger set of parameters. 4. Ensemble learning, averaging the predictions from multiple models, improved prediction accuracy, achieving an R² of 0.954 and an RMSE of 0.034. 5. Validation with the 2018-2019 regular season data confirmed that NN Model-2 provided the best prediction accuracy among the three models, with all predicted team records falling within the 95% prediction interval. |
| 8 | Giasemidis, 2020 [49] | **Accuracy：**   - Simple ML models: Not greater than 67% on the test set - AdaBoost model: 75% accuracy using 5-fold cross-validation, 66.8% accuracy on hold-out set | 1. Simple ML models (LR, SVM, RF) achieve an accuracy not greater than 67%. 2. Feature selection using wrapper methods resulted in AdaBoost model achieving 75% accuracy via 5-fold cross-validation, but this dropped to 66.8% on a holdout set, which revealed the risk of overfitting. 3. The collective predictions of basketball enthusiasts (wisdom of the crowd) outperformed ML models with an accuracy of 73%. Future models should aim to exceed the 73% accuracy threshold set by human predictions. |
| 9 | Horvat et al. 2020 [41] | **Accuracy:**  *Train&Test validation method:*   - KNN: 57.90% (best) - DT: 53.49% (worst)   *Cross-validation method:*   - KNN: 58.95% (best) - DT: 53.37% (worst)   *Up-to-date data with Train&Test validation method:*   - KNN: 60.01% (best) - DT: 54.66% (worst) | 1. The KNN algorithm achieved the highest average accuracy (60.01% with up-to-date data), while DT performed worst (54.66%). 2. Basic feature averaging (no advanced engineering) provided moderate accuracy, but dynamic data updates improved models by 2.17%, suggesting real-time updates enhance representativeness. 3. Accuracy Determinants included validation method (cross-validation outperformed Train&Test but risks future data leakage), dataset recency, and season window length (smaller time windows yielded better results). |
| 10 | Huang et al. 2020 [50] | **RMSE:**   - Regression Tree (M5P): 0.9645 - Linear Regression: 1.3081 - Support Vector Regression: 2.4904   **Accuracy:**  Regression tree: 87.5% | 1. The regression tree model demonstrated the lowest RMSE among the three models tested (Regression Tree, Linear Regression, Support Vector Regression), indicating the highest prediction accuracy. 2. The regression tree model achieved an accuracy of 87.5% in predicting the outcomes of the Golden State Warriors’ games during the 2017-2018 NBA season. 3. The study highlighted the efficiency of the regression tree model in handling nonlinear data and providing interpretable results through regression equations. 4. Key factors included player shooting efficiency (FG%, 3P%) and minutes played. |
| 11 | Li,  2020 [51] | **Accuracy**   - Linear Regression: - 2015: 66.91% - 2016: 63.80% - 2017: 64.31% - LR: - 2015: 67.15% - 2016: 63.34% - 2017: 64.23% - SVM: - 2015: 66.34% - 2016: 63.73% - 2017: 64.23%   Accuracy after LASSO:   - 2015: 67.24% - 2016: 63.93% - 2017: 65.45% | 1. The accuracy of the models was measured for the 2015, 2016, and 2017 seasons, with LR showing the highest accuracy among the three models in 2015 and linear regression in 2017. 2. Feature selection methods like LASSO were employed to enhance prediction accuracy by reducing the number of features and addressing overfitting. 3. The application of LASSO improved the accuracy of linear regression models for all three years, achieving the highest improvement in the 2017 season (from 64.31% to 65.45%). 4. The study identified the top 14 features most correlated with game outcomes, including field goals made, two pointers attempted, three pointers made, free throws attempted, free throws made, defensive or offensive rebounds, and assists for both home and opposing teams. 5. The study also discussed the limitations of current models in predicting upsets and the potential improvements by including player injuries and playoff statistics. |
| 12 | Migliorat, 2020 [52] | **Accuracy:**   - CART (Box Score, without points and assists): 71.68% - CART (Four Factors, without shooting): 67.26% - RF (Box Score, without points and assists): 91.15% - RF (Four Factors): 93.81% | 1. Critical Accuracy Factors: Defensive rebounds and opponent turnover rates were dominant, alongside shooting efficiency, aligning with Dean’s Four Factors weights. 2. The RF model using four factors achieved the highest accuracy (93.81%), followed closely by the RF model using box score data (excluding points and assists) (91.15%). |
| 13 | Ozkan,  2020 [5] | **ANN Model:**   - Accuracy: 70.8% - Sensitivity: 54.5% - Specificity: 84.6%   **CNFS Model:**   - Accuracy: 79.2% - Sensitivity: 72.7% - Specificity: 79.1% | 1. The CNFS model significantly improved prediction accuracy over the standard ANN model. 2. The hybrid approach (CNFS) combining ANN and fuzzy logic leveraged the strengths of both methods, providing better decision-making capabilities. The CNFS model showed higher accuracy and sensitivity, particularly in predicting away team victories. |
| 14 | Song et al. 2020 [1] | **Accuracy:**   - Gamma process model: Outperforms naive model based on empirical frequency - Adjusted gamma process model: Higher accuracy than naive model and gamma process model for regular games | 1. The adjusted gamma process model incorporates the bookmaker’s betting line, improving prediction accuracy by accounting for differences between matches, outperforming a naive model based on empirical frequency and gamma process model. 2. The models demonstrated increasing accuracy as more in-game information became available, particularly for predicting relatively improbable events (e.g., total points exceeding 240 or being smaller than 170). 3. Key accuracy factors included real-time scoring updates (model leveraged minute-by-minute data), match-specific team ability differences (addressed via betting-line adjustments), and time remaining in the game (accuracy increased as play progressed). 4. The model provided effective dynamic in-play predictions, updating probabilities every minute and yielding positive returns in betting simulations (e.g., 10.9% average return across NBA seasons). |
| 15 | Ballı et al. 2021 [53] | **Accuracy:**   - MLP: 98.90% (Dataset 5 and Model 6) - LR: Various accuracies, up to 98.04% depending on dataset and model | 1. MLP with Model 6 achieved the highest accuracy (98.90%), outperforming LR and voting ensembles. 2. Combining the Four Factors and DefenseOfense models significantly improved prediction accuracy. |
| 16 | Chen et al. 2021 [40] | **MAPE**  Single models:   - ELM: 0.0870 - MARS: 0.0846 - XGBoost: 0.0842 - SGB: 0.0845 - KNN: 0.0873   Two-stage models:   - ELM: 0.0863 - MARS: 0.0845 - XGBoost: 0.0818 - SGB: 0.0829 - KNN: 0.0872   **RMSE:**   - Best two-stage XGBoost model: 11.4753   **Sum of Squared Errors (SSE)**:   - Best two-stage XGBoost model: 61,627.37 | 1. The two-stage XGBoost model using four pieces of game-lags information achieved the best prediction performance among all models with a MAPE value of 0.0818, outperforming single-stage models (e.g., S-XGBoost: 8.42%) and baselines. 2. The six designed features that significantly influenced the prediction included averaged defensive rebounds, two-point field goal percentage, free throw percentage, offensive rebounds, assists, and three-point field goal attempts. 3. Game-lag information of four games was identified as the most suitable for NBA game score prediction, outperforming models using other game-lag configurations. |
| 17 | Lu et al.  2021 [2] | **RMSE:**   - CART: Best RMSE = 11.7564 - RF: Best RMSE = 11.6303 - SGB: Best RMSE = 11.5586 - XGBoost: Best RMSE = 11.6941 - ELM: Best RMSE = 11.8020 | 1. The integration of adaptive weighted features and game-lag information significantly improved the prediction performance. 2. The best prediction performance was achieved using a weighting control parameter of 1 and 4 game-lags of information across the 5 ML methods (CART, RF, SGB, XGBoost, ELM), with the best performance achieved by SGB. 3. The weighting distribution with a linear decline (weighting control parameter of 1) performed better than non-linear weighting distributions, as it better captures the stable team performance over the last few games. |
| 18 | Chen et al. 2022 [21] | **Accuracy:**  61% using fuzzy theory | 1. The fuzzy logic-based model achieved 61% accuracy, surpassing statistical methods (MANOVA/CCA: 53%) and outperforming expert predictions (53%) and public fans (47%). 2. Fuzzy theory improved accuracy by ~8% by highlighting critical parameters (points per game, recent team status) over traditional averaging. |
| 19 | Khanmohammadi et al. 2022 [54] | **Area Under the ROC Curve:**   - MambaNet with team and player statistics: AUC ranged from 0.72 to 0.82 across different NBA seasons and datasets | 1. MambaNet model outperformed the baseline models, achieving an AUC score ranging from 0.72 to 0.82, which was a considerable improvement over the best-performing baseline models.  2. FINs improved AUC by 5-15% vs. traditional stat averaging, enhancing temporal and hierarchical feature representation.  3. The study demonstrated the generalizability of their MambaNet model by testing it on the Iranian Super League data, in addition to the NBA data. |
| 20 | Krishnan et al.  2022 [55] | **Accuracy:**   - LR: 66.24% - ANN: 68.08% | 1. ANN achieved higher accuracy compared to LR in predicting win-loss percentage, as its ability to capture complex nonlinear relationships between the features. 2. The study highlighted the importance of feature selection and preprocessing, using methods like one-hot encoding for categorical variables and correlation heat maps for feature selection. 3. ML models can effectively predict NBA game outcomes using a combination of traditional and advanced statistics. |
| 21 | Ma et al.  2022 [22] | **RMSE:**   - Linear Regression: 9.2558 - XGBoost: 8.9581 - NN: 9.0387   **MAE:**   - Linear Regression: 7.0478 - XGBoost: 6.8486 - NN: 6.8805 | 1. Compared with NN and Linear Regression,The XGBoost model with hyperparameter adjustment showed the best predictive performance, with the lowest RMSE and MAE values, leveraging gradient boosting for non-linear relationships. 2. Weighted averaging (quadratic weighting) reduced RMSE by ~10% compared to naive averaging; feature selection removed redundant variables (e.g., FG%). 3. Key feature variables for the model to predict a player’s ability include salary information, team, player sheet, and statistical factors such as total rebounds and individual points scored. |
| 22 | Osken et al. 2022 [23] | **Accuracy:**   - GA-ANN using c-means clustering with cosine distance: 76.52% - GA-ANN using k-means clustering with Euclidean distance (k=26): 76.29% - GA-ANN using k-means clustering with Euclidean distance (k=25): 75.36% - Naive predictor (home team wins): 58% - Human experts: 65%-68% | 1. The study used a complex systems approach to predict NBA game outcomes by identifying different “player types” through clustering analysis (k-means and c-means), rather than using traditional basketball positions, with the best prediction accuracy achieved by c-means with cosine distance and k-means with Euclidean distance. 2. The GA-ANN models significantly outperformed the naive predictor and human experts in terms of prediction accuracy. 3. The study highlighted the importance of player clustering to capture synergies and interactions among players, which traditional player positions fail to adequately represent. 4. The model’s prediction accuracy remained competitive even without including team-level “Win %” statistics, validating the approach's reliance on player types. Including some team-level variables like rest days and win percentage also improved the prediction accuracy, suggesting that both player-level and team-level factors influence game outcomes. |
| 23 | Sikka et al. 2022 [56] | **R² and RMSE for various models:**   - Linear Regression + RF: R² = 0.9298, RMSE = 0.0373 - RF + Gaussian Process: R² = 0.9332, RMSE = 0.0365 - Final Ensemble (all five models): R² = 0.9332, RMSE = 0.0364 | 1. The ensemble-based ML model (MLR, DT, RF, GB, and Gaussian Process Regression) achieved an R^2^ score of 0.9332 and an RMSE of 0.0364, indicating a high level of accuracy in predicting the win percentage of NBA teams over a season.  2. Efficiency differentials and strength of schedule boosted accuracy by capturing team dynamics, improving R² compared to models without these features. |
| 24 | Su et al. 2022 [57] | **RMSE:**   - XGBoost: 15.92 (initial set), 15.17 (filtered set) - RF: 23.84 (initial set), 26.12 (filtered set) - BPNN: 98.88 (initial set), 98.88 (filtered set) - GRNN: 381.28 (initial set), 54.11 (filtered set)   **MAPE:**   - XGBoost: 2.44 (initial set), 2.13 (filtered set) - RF: 5.75 (initial set), 5.16 (filtered set) - BPNN: 16.44 (initial set), 22.71 (filtered set) - GRNN: 215.70 (initial set), 11.66 (filtered set) | 1. The XGBoost outperformed other methods like RF, BPNN, and GRNN in predicting NBA player scores, with an average MAPE of only 2-3.  2. Feature selection by correlation analysis improved the prediction accuracy compared to using the full set of features.  3. The key factors influencing NBA player scores include number of two-pointers, three-pointers, free throws, and player salary.  4. The study propose integrating their XGBoost model with the latest NBA season data to provide analysis and prediction results for NBA teams, coaches, and management. |
| 25 | Santos et al. 2022 [58] | **Accuracy**   - LR: 68.58% - Linear Support Vector Machines: 68.18% - RF: 69.88% - MLP: 68.85% | 1. RF achieved the highest prediction accuracy (69.88%), outperforming LR, LSVM, and MLP. 2. Feature selection (e.g., using home team status, player performance metrics, and salary ratios) contributed to RF’s superior accuracy. 3. Accuracy critical factors included home team advantage, player performance from the previous season, recent performance (last 15 games), salary cap ratios, and historical Four Factors (eFG%, TOV%, rebounds, FT rate). 4. RF was equally successful in picking a champion as the odds makers and consistently outperformed the ELO approach for all playoff rounds (except one in 2014-2015). |
| 26 | Wang et al. 2022 [59] | **For Win Ratio Prediction:**   - RF: R² = 0.65, MSE = 0.014 - FFNN: R² = 0.77, MSE = 0.009   **For Post-season Playoff Classification:**   - RF: F1 = 0.85 - FFNN: F1 = 0.87 | 1. The FFNN model outperformed the RF model in both win ratio prediction and playoff classification. 2. LIME method was used to interpret the NN model’s predictions, providing insights into the key features driving the predictions, providing sensible reasoning that validated common basketball theories such as the importance of defense over offense for team success. 3. Defensive and offensive ratings, floor impact, and field goal percentages were significant factors contributing to high win ratios and playoff qualifications. 4. The clustering analysis revealed distinct gameplay styles over different NBA eras, highlighting the evolution of game strategies and player performance metrics. |
| 27 | Zheng, 2022 [60] | **Accuracy:**   - Best RF model: 67.98% - LR with Sequential Forward Selection on Feature Set C: 67.39% - NB with SFS on Feature Set C: 67.48% - FFNN on Feature Set B: 67.58% - NB on Feature Set B: 67.88% | 1. RF outperformed others (67.98% accuracy), followed by NB (67.88%) and FFNN (67.58%). Feature set C (difference features + tiredness/Elo) yielded the best results. 2. Novel features (tiredness levels, difference features) improved accuracy by 1.5-2%, validating the importance of integrating external factors. 3. Accuracy critical predictors included Elo rating difference, home-court advantage, recent performance (10-game averages), and tiredness levels. |
| 28 | Daundkar et al. 2023 [61] | **Accuracy:**  2018 season:   - RF: 60.8% to 65.5% - LR: 62.3% to 65.8%   2019 season:   - RF: 54.9% to 61.3% - LR: 57.1% to 61.3% | 1. RF and LR achieved the best accuracy (66% in 2018, 61% in 2019), outperforming SVM, KNN, and DT. 2. Feature selection (e.g., top k features via Chi-squared) did not improve accuracy, suggesting sufficient data and no overfitting. 3. Critical predictors included home team’s assists, points, defensive rebounds, and three-pointers. Accuracy was highly dependent on the number of past matches used for averaging. |
| 29 | Horvat et al. 2023 [6] | **Accuracy**   - Average accuracy: 66% - Maximum accuracy: 78% | 1. The proposed model achieved 66-78% accuracy, outperforming standard ML methods. 2. The extended team efficiency index with OTW-driven training data improved predictions, while home advantage and mutual game history were critical factors. 3. Dynamic OTW adaptation enhanced accuracy by prioritizing recent performance. |
| 30 | Lampis et al.  2023 [62] | **Brier Score, F1-score, and accuracy**  **Best performing model accuracy:**  Greek Basket League: 78%  Spanish Liga ACB: 72%  Euroleague: ～69%  Eurocup: ～69% | 1. Ensemble learning (combined LR, RF, XGBoost) showed highest accuracy, but no single algorithm dominated; differences between models were marginal (~3%). 2. Enhanced models (with Elo, PageRank, pi-rating) outperformed baseline (“vanilla” team-name model) by 3-5% accuracy, validating the importance of ratings and current form features. 3. The rating systems (player impact ratings, PageRank, Elo) and current form performance indicators are the most important predictors. 4. The prediction accuracy for different leagues varied, with the Greek league being the least balanced (78% accuracy) and the Spanish league being more predictable (72% accuracy). European tournaments had lower prediction accuracy (about 69%). 5. Current-season data sufficed for training, eliminating the need for historical data in dynamic scenarios like playoffs or mid-season predictions. |
| 31 | Patrot et al. 2023 [24] | **Accuracy:**   - Linear Regression: 92% - SVM: 85% - DT: 69% | 1. Linear regression achieved the highest accuracy, followed by SVM, and DT. 2. Feature selection methods such as correlation feature set and multiple regression were used to identify the most significant features influencing game outcomes. 3. Feature selection methods improved the prediction accuracy by 2%-4%. 4. Defensive rebounds, three-point percentage, free throws made, field goal percentage, and total rebounds were identified as significant factors affecting NBA game outcomes. |
| 32 | Wang,  2023 [63] | **Accuracy:**   - LR: 81.64% - SVM: 74.81% - LSTM: 75.31% - RF: 83.78%   **AUC (Area Under the Curve):**   - LR: 0.90 - SVM: 0.82 - LSTM: 0.83 - RF: 0.92 | 1. RF outperformed all models, surpassing both DNN and traditional methods (e.g., LR, SVM). 2. Feature engineering (e.g., removing PTS, prioritizing Field goal percentage) enhanced model performance. 3. Field goal percentage was the most influential predictor; other key features included defensive rebounds and assists. |
| 33 | Zhao et al. 2023 [7] | **Accuracy:**  GCN alone: 66.90%  GCN + RF: 71.54%  GCN + LASSO: 70.70%  GCN + PCA: 50.73% | 1. GCN + Random Forest outperformed all models (71.54%), surpassing traditional ML (SVM, LR) and baseline GCN. 2. Compared to the original GCN model, the GCN models derived from the RF and LASSO techniques demonstrated improved accuracy. However, the GCN + PCA model exhibited a decrease in accuracy. 3. The most important features identified were efficiency differential for team, defensive rating for team, and floor impact counter for team. |
| 34 | Kandhway, 2024 [64] | **Accuracy**  Varies by quarter and classifier   - RF: 61.5% (Quarter 1) to 69.8% (Quarter 4) - SVM: 54.2% (Quarter 1) to 77.3% (Quarter 4) - NN: 59.0% (Quarter 1) to 78.2% (Quarter 4) - LR: 58.5% (Quarter 1) to 78.2% (Quarter 4) | 1. NN and LR outperformed others in later match stages (78.2% accuracy by Q4), while RF performed best initially (61.5% at Q1). 2. Incorporating real-time match metrics (e.g., points, rebounds) with historical win ratios improved prediction accuracy by 15-20% as the match progressed. 3. The most important features for prediction included the strength of the home team over the away team, points scored by both teams, three-pointers made, defensive rebounds, assists, and field goals made. 4. Accuracy increased dynamically from 62% (Q1) to 78% (Q4), demonstrating the value of real-time data integration. |

SVM: support vector machine, RBF kernel: radial basis function kernel, ML: machine learning, LSTM: Long Short-Term Memory, GLM: general linear model, MAE: mean absolute error, NB: naive bayes, ANN: artificial neural networks, LMT: logistic model tree, RIPPER: Repeated Incremental Pruning to Produce Error Reduction, NN: neural network, RMSE: root mean square error, KNN: k-nearest neighbors, DT: decision tree, LR: logistic regression, LASSO: Least Absolute Shrinkage and Selection Operator, CART: classification and regression trees, RF: random forest, CNFS: Concurrent Neuro Fuzzy System, MLP: multilayer perceptron, MAPE: mean absolute percentage error, SGB: stochastic gradient boosting, XGBoost: eXtreme gradient boosting, MARS: Multivariate Adaptive Regression Splines, ELM: extreme learning machine, ROC: receiver operating characteristic, AUC: area under the curve, RNN: recurrent neural network, GA: genetic algorithms, BPNN: backward neural network, GRNN: generalized regression neural network, MSE: mean square error, FFNN: feed forward neural network, LIME, Local Interpretable Model-agnostic Explanations, SFS: Selective Feature Selection, GCN: graph convolutional network, PCA: Principal component analysis.
