# Supplementary material for: The application of artificial intelligence techniques in predicting game outcomes of professional basketball league: A systematic review
Source: PLoS One. 2025 Jun 26;20(6):e0326326. doi: 10.1371/journal.pone.0326326 (PMC12200876; doi:10.1371/journal.pone.0326326)
Supplement: S5 Table — (DOCX) [file pone.0326326.s008.docx]

| Study ID | Authors | Title | Include/ Exclude (Reason) |
| --- | --- | --- | --- |
| [1] | NA | Cristina Mayor‐Ruiz | Exclude: Irrelevant to the research topic |
| [2] | Memmert, D | Teaching Games For Understanding Conference Supplement From The German Sport University | Exclude: Irrelevant to the research topic |
| [3] | AARONS M F, YOUNG C M, BRUCE L, et al. | Real Time Prediction Of Match Outcomes In Australian Football | Exclude: Irrelevant to the research topic |
| [4] | ABREU P, MOURA J, SILVA D, et al. | Performance Analysis In Soccer: A Cartesian Coordinates Based Approach Using Robocup Data | Exclude: Irrelevant to the research topic |
| [5] | ACIKMESE Y, USTUNDAG B C, GOLUBOVIC E, et al. | Towards An Artificial Training Expert System For Basketball | Exclude: Irrelevant to the research topic |
| [6] | ADAMS R P, DAHL G E, MURRAY I. | Incorporating Side Information In Probabilistic Matrix Factorization With Gaussian Processes | Exclude: Irrelevant to the research topic |
| [7] | AFROUZEH M, KONUKMAN F, MUSA R M, et al. | Kinematic Variables Defining Performance Of Basketball Free-Throw In Novice Children: An Information Gain And Logistic Regression Analysis | Exclude: Irrelevant to the research topic |
| [8] | AGBINYA J I, REES D. | Multi-Object Tracking In Video | Exclude: Irrelevant to the research topic |
| [9] | AGBOZO E, PANDYA K, JOVANOVIC P, et al. | A Comprehensive Overview Of Artificial Intelligence Applications In Basketball | Exclude: Irrelevant to the research topic |
| [10] | AHMADALINEZHAD M, MAKREHCHI M, SEWARD N. | Basketball Lineup Performance Prediction Using Network Analysis | Exclude: Irrelevant to the research topic |
| [11] | AI S, NA J, SILVA V D, et al. | A Novel Methodology For Automating Spatio-Temporal Data Classification In Basketball Using Active Learning; | Exclude: Irrelevant to the research topic |
| [12] | AKCAY M M, SEYMEN M, ER O, et al. | Sport News Classification With Convolutional Neural Network And Long-Short Term Memory | Exclude: Irrelevant to the research topic |
| [13] | ALAMEDA-BASORA E, RYAN S. | Application Of Bayesian Network To Total Points In NBA Games | Include |
| [14] | ALDAHOUL N, KARIM H A, DATTA R, et al. | Convolutional Neural Network-Long Short Term Memory Based IOT Node For Violence Detection | Exclude: Irrelevant to the research topic |
| [15] | AL-GABALAWY M. | Path Planning Of Robotic Arm Based On Deep Reinforcement Learning Algorithm | Exclude: Irrelevant to the research topic |
| [16] | ALLEY M, BIGGS M, HARISS R, et al. | Pricing For Heterogeneous Products: Analytics For Ticket Reselling | Exclude: Irrelevant to the research topic |
| [17] | ALONSO J M. | Explainable Artificial Intelligence For Kids | Exclude: Irrelevant to the research topic |
| [18] | ANDREAS R, JACK A I. | Trust Or Interaction? Editorial Introduction | Exclude: Irrelevant to the research topic |
| [19] | ANG Z. | Application Of Iot Technology Based On Neural Networks In Basketball Training Motion Capture And Injury Prevention | Exclude: Irrelevant to the research topic |
| [20] | ANTONIONI E, SURIANI V, SOLIMANDO F, et al. | Learning From The Crowd: Improving The Decision Making Process In Robot Soccer Using The Audience Noise | Exclude: Irrelevant to the research topic |
| [21] | ANTONY J W, HARTSHORNE T H, POMEROY K, et al. | Behavioral, Physiological, And Neural Signatures Of Surprise During Naturalistic Sports Viewing | Exclude: Irrelevant to the research topic |
| [22] | ARAMPATZIS D, DOULGERAKI M, GIANNOULIS M, et al. | An Educational Platform For Logic-Based Reasoning | Exclude: Irrelevant to the research topic |
| [23] | ARMSTRONG W W, LI D W. | A New Technique For Reinforcement Learning For Control | Exclude: Irrelevant to the research topic |
| [24] | ARPITHA T C, SANJAY H S, KIRAN KUMAR H K, et al. | Machine Learning Based Prediction Of The Best Suitable Playing Positions Of The Players In The Game Of Basketball | Exclude: Irrelevant to the research topic |
| [25] | ASHOUR M A H, AL-DAHHAN I A H, AL-QABILY S M A. | Solving Game Theory Problems Using Linear Programming And Genetic Algorithms | Exclude: Irrelevant to the research topic |
| [26] | BAI Y. | Accurate Prediction Of NBA Players’ Lifespan With Big Sports Data | Exclude: Irrelevant to the research topic |
| [27] | BAI Z, BAI X. | Towards Understanding The Analysis, Models, And Future Directions Of Sports Social Networks | Exclude: Irrelevant to the research topic |
| [28] | BALLI S, KORUKOGLU S. | Development Of A Fuzzy Decision Support Framework For Complex Multi-Attribute Decision Problems: A Case Study For The Selection Of Skilful Basketball Players | Exclude: Irrelevant to the research topic |
| [29] | BALLı S, ÖZDEMIR E. | A Novel Method For Prediction Of Euroleague Game Results Using Hybrid Feature Extraction And Machine Learning Techniques | Include |
| [30] | BAO W, BAI Y. | Research On Basketball Footwork Recognition Based On A Convolutional Neural Network Algorithm | Exclude: Irrelevant to the research topic |
| [31] | BARBU M C R, POPESCU M C, BURCEA G B, et al. | Sustainability And Social Responsibility Of Romanian Sport Organizations | Exclude: Irrelevant to the research topic |
| [32] | BARTLETT R, LAMB P, ROBBINS A. | Use Of Self Organizing Maps In Technique Analysis | Exclude: Irrelevant to the research topic |
| [33] | BEDANOKOVA L S. | The Influence Of Physical Loadings On Cognitive Functions Of Students | Exclude: Irrelevant to the research topic |
| [34] | BENSON L C, STILLING C, OWOEYE O B A, et al. | Evaluating Methods For Imputing Missing Data From Longitudinal Monitoring Of Athlete Workload | Exclude: Irrelevant to the research topic |
| [35] | BERTASIUS G, PARK H S, YU S X, et al. | Am I A Baller? Basketball Performance Assessment From First-Person Videos | Exclude: Irrelevant to the research topic |
| [36] | BERTASIUS G, SHI J, IEEE. | Using Cross-Model Ego Supervision To Learn Cooperative Basketball Intention | Exclude: Irrelevant to the research topic |
| [37] | BHAT H S, HUANG L H, RODRIGUEZ S. | Learning Stochastic Models For Basketball Substitutions From Play-By-Play Data | Exclude: Irrelevant to the research topic |
| [38] | BIANCHI F, FACCHINETTI T, ZUCCOLOTTO P. | Role Revolution: Towards A New Meaning Of Positions In Basketball | Exclude: Irrelevant to the research topic |
| [39] | [Yang Bo](https://onlinelibrary.wiley.com/authored-by/Bo/Yang). | A Reinforcement Learning-Based Basketball Player Activity Recognition Method Using Multisensors | Exclude: Irrelevant to the research topic |
| [40] | BOGERS S, MEGENS C, VOS S. | Design For Balanced Engagement In Mixed Level Sports Teams | Exclude: Irrelevant to the research topic |
| [41] | BONNER J, WOODWARD C J. | On Domain-Specific Decision Support Systems For E-Sports Strategy Games | Exclude: Irrelevant to the research topic |
| [42] | BRANCACCIO M, MENNITTI C, CESARO A, et al. | Multidisciplinary In-Depth Investigation In A Young Athlete Suffering From Syncope Caused By Myocardial Bridge | Exclude: Irrelevant to the research topic |
| [43] | BROGNAUX S, FRANçOIS T, SAERENS M. | Combining Manual And Automatic Prosodic Annotation For Expressive Speech Synthesis | Exclude: Irrelevant to the research topic |
| [44] | BROMS L. | Fans, Fellows Or Followers: A Study On How Sport Federations Shape Social Media Affordances | Exclude: Irrelevant to the research topic |
| [45] | BU F, XU S, HELLER K, et al. | SMOGS: Social Network Metrics Of Game Success | Exclude: Irrelevant to the research topic |
| [46] | [R Ma](https://xueshu.baidu.com/s?wd=author:(R%20Ma)%20&tn=SE_baiduxueshu_c1gjeupa&ie=utf-8&sc_f_para=sc_hilight=person)，[Z Zhang](https://xueshu.baidu.com/s?wd=author:(Z%20Zhang)%20&tn=SE_baiduxueshu_c1gjeupa&ie=utf-8&sc_f_para=sc_hilight=person)，[E Chen](https://xueshu.baidu.com/s?wd=author:(E%20Chen)%20&tn=SE_baiduxueshu_c1gjeupa&ie=utf-8&sc_f_para=sc_hilight=person). | Human Motion Gesture Recognition Algorithm In Video Based On Convolutional Neural Features Of Training Images | Exclude: Irrelevant to the research topic |
| [47] | BUFORD MAY R A. | The Good And Bad Of It All: Professional Black Male Basketball Players As Role Models For Young Black Male Basketball Players | Exclude: Irrelevant to the research topic |
| [48] | BUSTAMANTE M A, CORSO J J. | Using Probabilistic Ontologies For Video Exploration | Exclude: Irrelevant to the research topic |
| [49] | CAI H. | The Detection, Segmentation, And Altitude Estimation Of Sports Players' Videos Under Deep Learning | Exclude: Irrelevant to the research topic |
| [50] | CAI X. | WSN-Driven Posture Recognition And Correction Towards Basketball Exercise | Exclude: Irrelevant to the research topic |
| [51] | CAIO M D, VAN ZANDYCKE G, DE VLEESCHOUWER C, et al. | Context-Aware 3D Object Localization From Single Calibrated Images: A Study Of Basketballs | Exclude: Irrelevant to the research topic |
| [52] | CALIWAG J A, ARAGON M C R, CASTILLO R E, et al. | Predicting Basketball Results Using Cascading Algorithm | Exclude: Publication outside the 2019-2024 period |
| [53] | CAMENIDIS C-M, BĂIŢEL I, OATU A, et al. | Determination Of Neuromuscular Control Of The Upper Limbs In Children - Case Study | Exclude: Irrelevant to the research topic |
| [54] | CANDIA LUJAN R, CANDIA SOSA K F, ORTIZ GOMEZ O R, et al. | ÍNdice De AsimetrÍA Bilateral MorfolÓGica De Extremidades Inferiores Y Superiores En Jugadores De Baloncesto Universitario | Exclude: Were not written in English |
| [55] | CANNAVò A, CALANDRA D, BASILICò G, et al. | Automatic Recognition Of Sport Events From Spatio-Temporal Data: An Application For Virtual Reality-Based Training In Basketball | Exclude: Irrelevant to the research topic |
| [56] | CAO Y, PENG Y, SHEN Z, et al. | Application Of Tactics In Technical And Tactical Analysis Of Table Tennis Mixed Doubles Based On Artificial Intelligence Graph Theory Model | Exclude: Irrelevant to the research topic |
| [57] | CAO Y, WANG S, LI X, et al. | Inferring Social Network User’S Interest Based On Convolutional Neural Network | Exclude: Irrelevant to the research topic |
| [58] | CARAYANNI V, BOGDANIS G C, VLACHOPAPADOPOULOU E, et al. | Predicting VO2max In Children And Adolescents Aged Between 6 And 17 Using Physiological Characteristics And Participation In Sport Activities: A Cross-Sectional Study Comparing Different Regression Models Stratified By Gender | Exclude: Irrelevant to the research topic |
| [59] | CECCHIN A. | Oliver’s Four-Factor Model: Validation Through Causality | Exclude: Irrelevant to the research topic |
| [60] | ÇENE E. | What Is The Difference Between A Winning And A Losing Team: Insights From Euroleague Basketball | Exclude: Irrelevant to the research topic |
| [61] | CERASA A, SARICA A, MARTINO I, et al. | Increased Cerebellar Gray Matter Volume In Head Chefs | Exclude: Irrelevant to the research topic |
| [62] | CHARTIER T. | Valuing Data | Exclude: Irrelevant to the research topic |
| [63] | CHATURVEDI I, ONG Y-S, ARUMUGAM R V. | Deep Transfer Learning For Classification Of Time-Delayed Gaussian Networks | Exclude: Irrelevant to the research topic |
| [64] | CHATURVEDI I, ONG Y-S, TSANG I W, et al. | Learning Word Dependencies In Text By Means Of A Deep Recurrent Belief Network | Exclude: Irrelevant to the research topic |
| [65] | CHAUHAN A, ANAND T, JAUHARI T, et al. | Identifying Race And Gender Bias In Stable Diffusion AI Image Generation | Exclude: Irrelevant to the research topic |
| [66] | CHE Y, CHE K, LI Q. | Application Of Decision Tree In PE Teaching Analysis And Management Under The Background Of Big Data | Exclude: Irrelevant to the research topic |
| [67] | CHEN C, CHEN W, ZHOU S. | Object Detection Of Basketball Robot Based On Mobilenet-SSD | Exclude: Irrelevant to the research topic |
| [68] | CHEN C-C, CHANG C, LIN C-S, et al. | Video Based Basketball Shooting Prediction And Pose Suggestion System | Exclude: Irrelevant to the research topic |
| [69] | CHEN C-H, LIU T-L, WANG Y-S, et al. | Spatio-Temporal Learning Of Basketball Offensive Strategies | Exclude: Irrelevant to the research topic |
| [70] | CHEN F, XU J. | Deep Learning Algorithm-Based Wearable Device In Basketball Motion Dynamic Analysis | Exclude: Irrelevant to the research topic |
| [71] | CHEN H. | Building A Basketball Shooting Model Based On Neural Networks And A Genetic Algorithm | Exclude: Irrelevant to the research topic |
| [72] | CHEN J, LE H M, CARR P, et al. | Learning Online Smooth Predictors For Realtime Camera Planning Using Recurrent Decision Trees | Exclude: Irrelevant to the research topic |
| [73] | CHEN K, MEMMERT D, CAPARROS M G. | The Emerging Basketball Discipline: Unpacking Game Outcomes In The 3 X 3 Basketball Professional League Based On Performance Indicators And Contextual Variables | Exclude: Irrelevant to the research topic |
| [74] | CHEN L, WANG W. | Analysis Of Technical Features In Basketball Video Based On Deep Learning Algorithm [J]. Signal Processing: Image Communication, 2020, 83. | Exclude: Irrelevant to the research topic |
| [75] | CHEN M, SU F. | A Basketball Game Prediction System Based On Artificial Intelligence | Include |
| [76] | CHEN Q. | Detection And Fine Positioning Of Basketball Technical Characteristic Target Based On Deep Learning Algorithm | Exclude: Irrelevant to the research topic |
| [77] | CHEN W. | Internet Of Things And Edge Computing Model Optimization In Physical Training Status And Countermeasures In College Sports Basketball Optional Course | Exclude: Irrelevant to the research topic |
| [78] | CHEN W-C, TSAI W-L, CHANG H-H, et al. | Instant Basketball Defensive Trajectory Generation | Exclude: Irrelevant to the research topic |
| [79] | CHEN W-J, JHOU M-J, LEE T-S, et al. | Hybrid Basketball Game Outcome Prediction Model By Integrating Data Mining Methods For The National Basketball Association | Include |
| [80] | CHEN X, JIANG J Y, JIN K, et al. | Reliable: Offline Reinforcement Learning For Tactical Strategies In Professional Basketball Games | Exclude: Irrelevant to the research topic |
| [81] | CHEN X, LIU Y. | A Classification Method For Thoracolumbar Vertebral Fractures Due To Basketball Sports Injury Based On Deep Learning | Exclude: Irrelevant to the research topic |
| [82] | CHEN Y. | Study On Neural Network Model-Based Senior High School Men's Basketball Training Strategy And Approach | Exclude: Irrelevant to the research topic |
| [83] | CHEN Y, DAI J, ZHANG C, et al. | A Neural Network Model Of The NBA Most Valued Player Selection Prediction | Exclude: Irrelevant to the research topic |
| [84] | CHEN Y, QIU Y, REN W. | A Normalized Score-Based Weighted Pagerank Algorithm On Ranking Prediction Of Basketball Games | Exclude: Irrelevant to the research topic |
| [85] | CHEN Y, YANG W, ZHANG T, et al. | Commander-Soldiers Reinforcement Learning For Cooperative Multi-Agent Systems | Exclude: Irrelevant to the research topic |
| [86] | CHEN Y, YANG W, ZHANG T, et al. | Application Of Network Information Technology In Physical Education And Training System Under The Background Of Big Data | Exclude: Irrelevant to the research topic |
| [87] | CHEN Z. | Application Of Computer Virtual Technology In Basketball Training | Exclude: Irrelevant to the research topic |
| [88] | CHEN Z, IEEE. | A Kind Of Basketball Teaching System Design Based On Network | Exclude: Irrelevant to the research topic |
| [89] | CHEN Z, ZHANG G. | CNN Sensor Based Motion Capture System Application In Basketball Training And Injury Prevention | Exclude: Irrelevant to the research topic |
| [90] | CHENG C Y, CHEN Y J, LIN S Y. | Design And Implementation Of A Vision-Based Basketball Shooting Robot | Exclude: Irrelevant to the research topic |
| [91] | CHENG Y, LIANG X, XU Y, et al. | Artificial Intelligence Technology In Basketball Training Action Recognition | Exclude: Irrelevant to the research topic |
| [92] | CHI Y, LI J. | Concrete Application Of Computer Virtual Image Technology In Modern Sports Training | Exclude: Irrelevant to the research topic |
| [93] | CHIDAMBARAM S, MAHESWARAN Y, PATEL K, et al. | Using Artificial Intelligence-Enhanced Sensing And Wearable Technology In Sports Medicine And Performance Optimisation | Exclude: Irrelevant to the research topic |
| [94] | CHIURA T B, VAN DER HAAR D. | Offensive Play Recognition Of Basketball Video Footage Using Actionformer | Exclude: Irrelevant to the research topic |
| [95] | CHOE D, JO Y, KANG S, et al. | UCSM-DNN: User And Card Style Modeling With Deep Neural Networks For Personalized Game AI | Exclude: Irrelevant to the research topic |
| [96] | CHOI H, 고병구. | Cluster Analysis Of Wrestlers Using A Self-Organizing Map As An Artificial Intelligent Technique | Exclude: Irrelevant to the research topic |
| [97] | CHOI T, CHO K, SUNG Y. | Approaches That Use Domain-Specific Expertise: Behavioral-Cloning-Based Advantage Actor-Critic In Basketball Games | Exclude: Irrelevant to the research topic |
| [98] | CHOMATEK L, SIERAKOWSKA K. | Automation Of Basketball Match Data Management | Exclude: Irrelevant to the research topic |
| [99] | CHOU P-H, CHIEN T-W, YANG T-Y, et al. | Predicting Active NBA Players Most Likely To Be Inducted Into The Basketball Hall Of Famers Using Artificial Neural Networks In Microsoft Excel: Development And Usability Study | Exclude: Irrelevant to the research topic |
| [100] | CHOU P Y, LIN C H, KAO W C, et al. | A Temporal Scores Network For Basketball Foul Classification | Exclude: Irrelevant to the research topic |
| [101] | CHOW T-H, CHEN Y-S, TSAI W-C, et al. | Plantar Pressure Profiles And Possible Foot Syndromes Of Taiwanese College Elite Basketball Players | Exclude: Irrelevant to the research topic |
| [102] | CHUNG J, WUU C-H, YANG H-R, et al. | HAA500: Human-Centric Atomic Action Dataset With Curated Videos | Exclude: Irrelevant to the research topic |
| [103] | CHUTIMON K, ANTONIO T, SALVADOR J J, et al. | Branched-Chain Amino Acids Supplementation Does Not Accelerate Recovery After A Change Of Direction Sprinting Exercise Protocol | Exclude: Irrelevant to the research topic |
| [104] | CIOPPA A, DELIEGE A, ISTASSE M, et al. | Arthus: Adaptive Real-Time Human Segmentation In Sports Through Online Distillation | Exclude: Irrelevant to the research topic |
| [105] | CLAUDINO J G, CAPANEMA D D O, DE SOUZA T V, et al. | Current Approaches To The Use Of Artificial Intelligence For Injury Risk Assessment And Performance Prediction In Team Sports: A Systematic Review | Exclude: Irrelevant to the research topic |
| [106] | COHAN A, SCHUSTER J, FERNANDEZ J. | A Deep Learning Approach To Injury Forecasting In NBA Basketball | Exclude: Irrelevant to the research topic |
| [107] | CONCI N, DE NATALE F G B, DALPONTE M, et al. | Accessible Video Analytics: The Use Case Of Basketball | Exclude: Irrelevant to the research topic |
| [108] | CONN K, LIU C, SARKAR N, et al. | Affect-Sensitive Assistive Intervention Technologies For Children With Autism: An Individual-Specific Approach | Exclude: Irrelevant to the research topic |
| [109] | CONSOLI F, ROGERS J, AL-DEEK H, et al. | Smart Event Traffic Management | Exclude: Irrelevant to the research topic |
| [110] | COVACI A, TALABA D. | Correlations In Basketball Free Throw | Exclude: Irrelevant to the research topic |
| [111] | CUI Z, LI X, GUO J, et al. | Sports Injury Early Warning Of Basketball Players Based On RBF Neural Network Algorithm | Exclude: Irrelevant to the research topic |
| [112] | CUST E E, SWEETING A J, BALL K, et al. | The Relationship Of Team And Individual Athlete Performances On Match Quarter Outcome In Elite Women's Australian Rules Football | Exclude: Irrelevant to the research topic |
| [113] | CUTLER M C, COOK M R, TRANSTRUM M K, et al. | Data-Driven Decomposition Of Crowd Noise From Indoor Sporting Events | Exclude: Irrelevant to the research topic |
| [114] | DABKE D V, CHAZELLE B. | Extracting Semantic Information From Dynamic Graphs Of Geometric Data | Exclude: Irrelevant to the research topic |
| [115] | DAKIC J G, COOK J, HAY-SMITH J, et al. | Pelvic Floor Disorders Stop Women Exercising: A Survey Of 4556 Symptomatic Women | Exclude: Irrelevant to the research topic |
| [116] | DAS M, MYRDEN S. | America’s Major League Soccer: Artificial Intelligence And The Quest To Become A World Class League | Exclude: Irrelevant to the research topic |
| [117] | DAS S, MAHMUD T, ISLAM D, et al. | Deep Transfer Learning-Based Foot No-Ball Detection In Live Cricket Match | Exclude: Irrelevant to the research topic |
| [118] | DAUNDKAR D, KANDHWAY K. | Predicting Winner Of A Professional Basketball Match | Include |
| [119] | DAVIDS K, BUTTON C, ARAúJO D, et al. | Movement Models From Sports Provide Representative Task Constraints For Studying Adaptive Behavior In Human Movement Systems | Exclude: Irrelevant to the research topic |
| [120] | DE CAMPOS SOUZA P V, LUGHOFER E. | An Explainable Evolving Fuzzy Neural Network In Position Identification Of Basketball Players | Exclude: Irrelevant to the research topic |
| [121] | DE LA TORRE R, CALVET L O, LOPEZ-LOPEZ D, et al. | Business Analytics In Sport Talent Acquisition: Methods, Experiences, And Open Research Opportunities | Exclude: Irrelevant to the research topic |
| [122] | DECROOS T, VAN HAAREN J, DZYUBA V, et al. | STARSS: A Spatio-Temporal Action Rating System For Soccer | Exclude: Irrelevant to the research topic |
| [123] | DEHESA R, VAQUERA A, GONçALVES B, et al. | Key Game Indicators In Nba Players’ Performance Profiles | Exclude: Irrelevant to the research topic |
| [124] | DELEN D, COGDELL D, KASAP N. | A Comparative Analysis Of Data Mining Methods In Predicting NCAA Bowl Outcomes | Exclude: Irrelevant to the research topic |
| [125] | DENG H, ZHAO Z, XIONG Z, et al. | Clinical Characteristics Of 1124 Children With Epiphyseal Fractures | Exclude: Irrelevant to the research topic |
| [126] | DENG L Y, LIU Y-J, IEEE. | Semantic Analysis And Video Event Mining In Sports Video | Exclude: Irrelevant to the research topic |
| [127] | DENG Q, ZHOU W, REYNOSO L C. | Decision Support Model For Student Physical Exercise Health Promotion Based On Artificial Neural Network | Exclude: Irrelevant to the research topic |
| [128] | DEWAR S K S, HIREMATH A, PATIL S, et al. | Human Activity Recognition Using Lrcn | Exclude: Irrelevant to the research topic |
| [129] | DHINGRA B, COLE J R R, EISENSCHLOS J M, et al. | Time-Aware Language Models As Temporal Knowledge Bases | Exclude: Irrelevant to the research topic |
| [130] | DING W, YU X, YE N. | Goal Detection For Broadcast Basketball Video Using Superimposed Texts: A Transition Pattern Approach | Exclude: Irrelevant to the research topic |
| [131] | DION E. | A Monte Carlo Assessment Of Suspense In Sports And Games | Exclude: Irrelevant to the research topic |
| [132] | DIOP C A, PELLOUX B, YU X, et al. | Soccer Player Recognition Using Artificial Intelligence And Computer Vision | Exclude: Irrelevant to the research topic |
| [133] | DONG C, SHEN Y, LIN S, et al. | A Unified Framework For Contextual And Factoid Question Generation | Exclude: Irrelevant to the research topic |
| [134] | DREYER F, GREIF J, GUENTHER K, et al. | Data-Driven Prediction Of Athletes' Performance Based On Their Social Media Presence | Exclude: Irrelevant to the research topic |
| [135] | DU W. | The Computer Vision Simulation Of Athlete's Wrong Actions Recognition Model Based On Artificial Intelligence | Exclude: Irrelevant to the research topic |
| [136] | DU X, CAI W. | Simulating A Basketball Game With Hdp-Based Models And Forecasting The Outcome | Exclude: Publication outside the 2019-2024 period |
| [137] | DUAN L Y, JIN J S, TIAN Q, et al. | Nonparametric Motion Characterization For Robust Classification Of Camera Motion Patterns | Exclude: Irrelevant to the research topic |
| [138] | DUAN L Y, XU M, TIAN Q. | Semantic Shot Classification In Sports Video | Exclude: Irrelevant to the research topic |
| [139] | DUAN L Y, XU M, TIAN Q, et al. | A Unified Framework For Semantic Shot Classification In Sports Video | Exclude: Irrelevant to the research topic |
| [140] | DUAN R, SUN P, SATAPATHY S C, et al. | Basketball Sports Neural Network Model Based On Nonlinear Classification | Exclude: Irrelevant to the research topic |
| [141] | DUBBS A. | Statistics-Free Sports Prediction | Exclude: Focus on non-professional basketball leagues |
| [142] | DURAND S, ROHAN C P, HAMILTON T, et al. | Passive Wrist Stiffness: The Influence Of Handedness | Exclude: Irrelevant to the research topic |
| [143] | EBERLE G, BOURLOT J, MARTINEZ C, et al. | Automatic Extraction Of Heat Maps And Goal Instances Of A Basketball Game Using Video Processing | Exclude: Irrelevant to the research topic |
| [144] | ELDER H, CANFIELD C, SHANK D B, et al. | Knowing When To Pass: The Effect Of AI Reliability In Risky Decision Contexts | Exclude: Irrelevant to the research topic |
| [145] | EUN-HYUNG C, SEOK C J. | Comparison Of The Outcomes Of Statistical Models Applied To The Prediction Of Play-Off Entry In Korean Professional Basketball | Exclude: Publication outside the 2019-2024 period |
| [146] | EVANS E J, JONES R, LEUNG J, et al. | Using Social Networks To Improve Team Transition Prediction In Professional Sports | Exclude: Irrelevant to the research topic |
| [147] | FAN J, BI S, WANG G, et al. | Sensor Fusion Basketball Shooting Posture Recognition System Based On CNN | Exclude: Irrelevant to the research topic |
| [148] | FAN J, BI S, XU R, et al. | Hybrid Lightweight Deep-Learning Model For Sensor-Fusion Basketball Shooting-Posture Recognition | Exclude: Irrelevant to the research topic |
| [149] | FAN Z, XIE J K, WANG Z Y, et al. | Image Classification Method Based On Improved KNN Algorithm | Exclude: Irrelevant to the research topic |
| [150] | FANG H. | Design Of Basketball Player Training Action Error Correction System Based On Convolutional Neural Network Algorithm | Exclude: Irrelevant to the research topic |
| [151] | FANG Y, YANG X. | Application Research Of The Virtual Visualization Technique In Basketball Teaching | Exclude: Irrelevant to the research topic |
| [152] | FANG Z, YU T, WANG Q, et al. | Use Of Ant Colony Optimization And The Kalman Filter To Deduce Thigh Dip Angle Via Acceleration And Angular Velocity Sensing | Exclude: Irrelevant to the research topic |
| [153] | FARHADIAN M, TORKAMAN S, MOJARAD F. | Random Forest Algorithm To Identify Factors Associated With Sports-Related Dental Injuries In 6 To 13-Year-Old Athlete Children In Hamadan, Iran-2018 -A Cross-Sectional Study | Exclude: Irrelevant to the research topic |
| [154] | FELSEN P, AGRAWAL P, MALIK J, et al. | What Will Happen Next? Forecasting Player Moves In Sports Videos | Exclude: Irrelevant to the research topic |
| [155] | FELSEN P, LUCEY P, GANGULY S. | Where Will They Go? Predicting Fine-Grained Adversarial Multi-Agent Motion Using Conditional Variational Autoencoders | Exclude: Irrelevant to the research topic |
| [156] | FENG X, SUN Y, QIN B, et al. | Learning To Select Bi-Aspect Information For Document-Scale Text Content Manipulation | Exclude: Irrelevant to the research topic |
| [157] | FENG Y, SUN H. | Basketball Footwork And Application Supported By Deep Learning Unsupervised Transfer Method | Exclude: Irrelevant to the research topic |
| [158] | FIALHO G, MANHãES A, TEIXEIRA J P. | Predicting Sports Results With Artificial Intelligence - A Proposal Framework For Soccer Games | Exclude: Irrelevant to the research topic |
| [159] | FOGARTY J, TAN D, KAPOOR A, et al. | Cueflik: Interactive Concept Learning In Image Search | Exclude: Irrelevant to the research topic |
| [160] | FU Q, ZHANG X, LI H. | Convolutional Neural Network-Based Restoration Method Of Basketball Contour Image | Exclude: Irrelevant to the research topic |
| [161] | FU X-B, YUE S-L, PAN D-Y. | Camera-Based Basketball Scoring Detection Using Convolutional Neural Network | Exclude: Irrelevant to the research topic |
| [162] | FU Y. | Evaluation Of College Basketball Teaching Based On Deep Learning | Exclude: Irrelevant to the research topic |
| [163] | FUJII K. | Data-Driven Analysis For Understanding Team Sports Behaviors | Exclude: Irrelevant to the research topic |
| [164] | FUJII K, TAKEISHI N, KAWAHARA Y, et al. | Decentralized Policy Learning With Partial Observation And Mechanical Constraints For Multiperson Modeling | Exclude: Irrelevant to the research topic |
| [165] | FUJII K, TAKEUCHI K, KURIBAYASHI A, et al. | Estimating Counterfactual Treatment Outcomes Over Time In Complex Multiagent Scenarios | Exclude: Irrelevant to the research topic |
| [166] | GANESH Y, TEJA A S, MUNNANGI S K, et al. | A Novel Framework For Fine Grained Action Recognition In Soccer | Exclude: Irrelevant to the research topic |
| [167] | GAO F, WU W, JIN Y, et al. | A Sparse Attention Pipeline For Deepsportradar Basketball Player Instance Segmentation Challenge | Exclude: Irrelevant to the research topic |
| [168] | GAO J. | Basketball Posture Recognition Based On HOG Feature Extraction And Convolutional Neural Network | Exclude: Irrelevant to the research topic |
| [169] | GAO Y. | PGD: A Large-Scale Professional Go Dataset For Data-Driven Analytics | Exclude: Irrelevant to the research topic |
| [170] | GE C, ZHENYU Z, KYEBAMBE M N, et al. | Predicting The Outcome Of NBA Playoffs Based On The Maximum Entropy Principle | Exclude: Publication outside the 2019-2024 period |
| [171] | GEORGIEVSKI B, VRTAGIC S. | Machine Learning And The NBA Game | Exclude: Absence of specific empirical data |
| [172] | GHADIRZADEH A, POKLUKAR P, ARNDT K, et al. | Training And Evaluation Of Deep Policies Using Reinforcement Learning And Generative Models | Exclude: Irrelevant to the research topic |
| [173] | GILLETT J, DE WITT J, STAHL C A, et al. | Descriptive And Kinetic Analysis Of Two Different Vertical Jump Tests Among Youth And Adolescent Male Basketball Athletes Using A Supervised Machine Learning Approach | Exclude: Irrelevant to the research topic |
| [174] | GIULIODORI P. | An Artificial Neural Network-Based Prediction Model For Underdog Teams In NBA Matches | Exclude: Irrelevant to the research topic |
| [175] | GOLDMAN M, RAO J M. | Optimal Stopping In The NBA: Sequential Search And The Shot Clock | Exclude: Irrelevant to the research topic |
| [176] | GONG H, WATANABE N M, SOEBBING B P, et al. | Do Consumer Perceptions Of Tanking Impact Attendance At National Basketball Association Games? A Sentiment Analysis Approach | Exclude: Irrelevant to the research topic |
| [177] | GONG L, FENG X, YE D, et al. | Optmatch: Optimized Matchmaking Via Modeling The High-Order Interactions On The Arena | Exclude: Irrelevant to the research topic |
| [178] | GONG Y, FAN Z. | A Non-Battery Pressure Detection And Communication System For Basketball Game Referee Based On Piezoelectric Devices | Exclude: Irrelevant to the research topic |
| [179] | GONG Y, SRIVASTAVA G. | Multi-Target Trajectory Tracking In Multi-Frame Video Images Of Basketball Games Based On Deep Learning | Exclude: Irrelevant to the research topic |
| [180] | GRAßL I, GELDREICH K, FRASER G. | Data-Driven Analysis Of Gender Differences And Similarities In Scratch Programs | Exclude: Irrelevant to the research topic |
| [181] | GREGORY S. | Digital Dunks | Exclude: Irrelevant to the research topic |
| [182] | GRUDA D, OJO A. | All I Do Is Win, Win, Win No Matter What? Pre-Game Anxiety And Experience Predict Athletic Performance In The NBA | Exclude: Irrelevant to the research topic |
| [183] | GRUSHKO A, MOROZOVA O, OSTAPCHUK M, et al. | Perceptual-Cognitive Demands Of Esports And Team Sports: A Comparative Study | Exclude: Irrelevant to the research topic |
| [184] | GU X, XUE X, WANG F. | Fine-Grained Action Recognition On A Novel Basketball Dataset | Exclude: Irrelevant to the research topic |
| [185] | GUDMUNDSSON J, HORTON M. | Spatio-Temporal Analysis Of Team Sports | Exclude: Irrelevant to the research topic |
| [186] | GUMM J, BARRETT A, HU G. | A Machine Learning Strategy For Predicting March Madness Winners | Exclude: Focus on non-professional basketball leagues |
| [187] | GUN J. | Basketball Action Recognition Based On FPGA And Particle Image | Exclude: Irrelevant to the research topic |
| [188] | GUO D, DONG S, WANG Q, et al. | Enhanced Sensitivity And Detection Range Of A Flexible Pressure Sensor Utilizing A Nano-Cracked PVP Hierarchical Nanofiber Membrane Formed By Bii3 Sublimation | Exclude: Irrelevant to the research topic |
| [189] | GUO H, HAO Q. | Research On Aided Judgment Of Rural Sports Posture Based On Deep Learning | Exclude: Irrelevant to the research topic |
| [190] | GUO J. | Basketball Field - Goal Percentage Prediction Model Research And Application Based On BP Neural Network | Exclude: Irrelevant to the research topic |
| [191] | GUO T, TAO K, HU Q, et al. | Detection Of Ice Hockey Players And Teams Via A Two-Phase Cascaded CNN Model | Exclude: Irrelevant to the research topic |
| [192] | GUO X, BROWN E, CHAN P P K, et al. | Skill Level Classification In Basketball Free-Throws Using A Single Inertial Sensor | Exclude: Irrelevant to the research topic |
| [193] | GUO Z, ZHU K. | The Application Of Successful Physical Education Teaching Mode Integrating Deep Learning In Basketball Teaching | Exclude: Irrelevant to the research topic |
| [194] | GUOCHEN W, FEIHONG S, LI X. | Video Analysis Method Of Basketball Training Assistant Based On Deep Learning Theory During COVID-19 Spread | Exclude: Irrelevant to the research topic |
| [195] | GUPTA P, KINGSTON K A, O'MALLEY M, et al. | Advancements In Artificial Intelligence For Foot And Ankle Surgery: A Systematic Review | Exclude: Irrelevant to the research topic |
| [196] | HABEL K, DEUSER F, OSWALD N, et al. | CLIP-Reident: Contrastive Training For Player Re-Identification | Exclude: Irrelevant to the research topic |
| [197] | HADDAD M, ABBES Z, IBRAHIM R, et al. | Relationship Between Asymmetry Indices, Anthropometric Parameters, And Physical Fitness In Obese And Non-Obese High School Students | Exclude: Irrelevant to the research topic |
| [198] | HADDAD M, ABBES Z, ZARROUK N, et al. | Difference Asymmetry Between Preferred Dominant And Non-Dominant Legs In Muscular Power And Balance Among Sub-Elite Soccer Players In Qatar | Exclude: Irrelevant to the research topic |
| [199] | HAHM G-J, CHO K, IEEE. | An Approach Of Webcast Event Clustering For Sport Video Event Annotation | Exclude: Irrelevant to the research topic |
| [200] | HAMDAD L, BENATCHBA K, BELKHAM F, et al. | Basketball Analytics. Data Mining For Acquiring Performances | Exclude: Irrelevant to the research topic |
| [201] | HAMLAOUI M E, LAHSAINI M A, MOSELHY S H, et al. | Basketball Free Throw Analysis Using Object Detection Techniques | Exclude: Irrelevant to the research topic |
| [202] | HAO H. | Survey On Satisfaction Extent Of CUBA Men Basketball Players On The Administration Behaviors Of Their Coaches | Exclude: Irrelevant to the research topic |
| [203] | HAO Y, KRISTAL B S, HSU F, et al. | Predication Of NCAA Bracket Using Recurrent Neural Network And Combinatorial Fusion | Exclude: Focus on non-professional basketball leagues |
| [204] | HAO Y, ZHANG H, NGO C-W, et al. | Compact Bilinear Augmented Query Structured Attention For Sport Highlights Classification | Exclude: Irrelevant to the research topic |
| [205] | HARRIS A R, ROEBBER P J. | NBA Team Home Advantage: Identifying Key Factors Using An Artificial Neural Network | Exclude: Irrelevant to the research topic |
| [206] | HASSAN A, SCHRAPF N, TILP M. | The Prediction Of Action Positions In Team Handball By Non-Linear Hybrid Neural Networks | Exclude: Irrelevant to the research topic |
| [207] | HAURI S, DJURIC N, RADOSAVLJEVIC V, et al. | Multi-Modal Trajectory Prediction Of NBA Players | Exclude: Irrelevant to the research topic |
| [208] | HAURI S, VUCETIC S. | Group Activity Recognition In Basketball Tracking Data - Neural Embeddings In Team Sports (NETS) | Exclude: Irrelevant to the research topic |
| [209] | HAZARA M, KYRKI V. | Transferring Generalizable Motor Primitives From Simulation To Real World | Exclude: Irrelevant to the research topic |
| [210] | HAZARA M, LI X, KYRKI V, et al. | Active Incremental Learning Of A Contextual Skill Model | Exclude: Irrelevant to the research topic |
| [211] | HE J, LEI J, LI G. | Temporal Action Detection Based On Feature Pyramid Hierarchies | Exclude: Irrelevant to the research topic |
| [212] | HE L, LUO J, XU G. | Basketball Action Recognition Algorithm Based On Global Context Awareness | Exclude: Irrelevant to the research topic |
| [213] | HE R, FU Z, LIU Q, et al. | D3: Duplicate Detection Decontaminator For Multi-Athlete Tracking In Sports Videos | Exclude: Irrelevant to the research topic |
| [214] | HE R, FU Z, LIU Q, et al. | Learning Group Interaction For Sports Video Understanding From A Perspective Of Athlete | Exclude: Irrelevant to the research topic |
| [215] | HEDBERG S R. | AI & Taxes | Exclude: Irrelevant to the research topic |
| [216] | HEDBERG S R. | Searching For The Mother Lode: Tales Of The First Data Miners | Exclude: Irrelevant to the research topic |
| [217] | HEE H N, EOB Y D, CHOI S. | An Analysis On The Application Of The Fourth Industrial Revolution And The Sports Convergence Industry | Exclude: Irrelevant to the research topic |
| [218] | HOELZEMANN A, ROMERO J L, BOCK M, et al. | Hang-Time HAR: A Benchmark Dataset For Basketball Activity Recognition Using Wrist-Worn Inertial Sensors | Exclude: Irrelevant to the research topic |
| [219] | HOJO M, FUJII K, INABA Y, et al. | Automatically Recognizing Strategic Cooperative Behaviors In Various Situations Of A Team Sport | Exclude: Irrelevant to the research topic |
| [220] | HONG C. | Group Behavior Recognition Algorithm For Basketball Video Based On Hidden Tag Mining Strategy Under Gamification Teaching And Learning | Exclude: Irrelevant to the research topic |
| [221] | HONG X. | Kinect And Few-Shot Technology-Based Simulation Of Physical Fitness And Health Training Model For Basketball Players In Plateau Area | Exclude: Irrelevant to the research topic |
| [222] | HORE S, BHATTACHARYA T. | A Machine Learning Based Approach Towards Building A Sustainabilitymodel For NBA Players | Exclude: Irrelevant to the research topic |
| [223] | HORRACE W C, JUNG H, SANDERS S. | Network Competition And Team Chemistry In The NBA | Exclude: Irrelevant to the research topic |
| [224] | HORVAT T, HAVAŠ L, SRPAK D. | The Impact Of Selecting A Validation Method In Machine Learning On Predicting Basketball Game Outcomes | Include |
| [225] | HORVAT T, HAVAŠ L, SRPAK D, et al. | Data-Driven Basketball Web Application For Support In Making Decisions | Exclude: Irrelevant to the research topic |
| [226] | HORVAT T, JOB J. | Importance Of The Training Dataset Length In Basketball Game Outcome Prediction By Using Naive Classification Machine Learning Methods | Include |
| [227] | HORVAT T, JOB J, LOGOZAR R, et al. | A Data-Driven Machine Learning Algorithm For Predicting The Outcomes Of NBA Games | Include |
| [228] | HORVAT T, JOB J, MEDVED V. | Prediction Of Euroleague Games Based On Supervised Classification Algorithm K-Nearest Neighbours | Exclude: Publication outside the 2019-2024 period |
| [229] | HOSHINO R, KAWARABAYASHI K-I. | Scheduling Bipartite Tournaments To Minimize Total Travel Distance | Exclude: Irrelevant to the research topic |
| [230] | HOSHINO R, KAWARABAYASHI K I. | The Inter-League Extension Of The Traveling Tournament Problem And Its Application To Sports Scheduling | Exclude: Irrelevant to the research topic |
| [231] | HOST K, IVAŠIĆ-KOS M. | An Overview Of Human Action Recognition In Sports Based On Computer Vision | Exclude: Irrelevant to the research topic |
| [232] | HOU L. | Virtual Simulation Technology Of Wireless Sensor Neural Network In Basketball Movement Distribution | Exclude: Irrelevant to the research topic |
| [233] | HOU S, LIAN B, LI W, et al. | A Basketball Training Posture Monitoring Algorithm Based On Machine Learning And Artificial Intelligence | Exclude: Irrelevant to the research topic |
| [234] | HOU X, JI Q. | Research On The Recognition Algorithm Of Basketball Technical Action Based On BP Neural System | Exclude: Irrelevant to the research topic |
| [235] | HSIA C-H, CHIEN C-H, HSU H-W, et al. | Sports Science: The Correction Of A Sportsperson's Pose Using A Knowledge-Based Method . | Exclude: Irrelevant to the research topic |
| [236] | HSU C, LOECHER N, PARK A L, et al. | Chronic Pain In Young Athletes: The Impact Of Athletic Identity On Pain-Related Distress And Functioning | Exclude: Irrelevant to the research topic |
| [237] | HSU Y-C. | Using Machine Learning And Candlestick Patterns To Predict The Outcomes Of American Football Games | Exclude: Irrelevant to the research topic |
| [238] | HSU Y-L, CHANG H-C, CHIU Y-J. | Wearable Sport Activity Classification Based On Deep Convolutional Neural Network | Exclude: Irrelevant to the research topic |
| [239] | HU P, WEN J. | Research On 3D Animation Character Design Based On Multimedia Interaction | Exclude: Irrelevant to the research topic |
| [240] | HU S, FU M. | Football Match Results Predicting By Machine Learning Techniques | Exclude: Irrelevant to the research topic |
| [241] | HU W. | The Application Of Artificial Intelligence And Big Data Technology In Basketball Sports Training | Exclude: Irrelevant to the research topic |
| [242] | HU X, MO S, PENG D, et al. | Automatic Activity Classification Based On Human Body Kinematics And Dynamic Time Wrapping | Exclude: Irrelevant to the research topic |
| [243] | HU X, MO S, QU X. | Basketball Activity Classification Based On Upper Body Kinematics And Dynamic Time Warping | Exclude: Irrelevant to the research topic |
| [244] | HU Y, LIU J. | Research On Attack Simulation Training System Of Basketball Players Under Computer Artificial Intelligence Assisted Technology | Exclude: Irrelevant to the research topic |
| [245] | HUA L, LIU G. | Development Of Basketball Tactics Basic Cooperation Teaching System Based On CNN And BP Neural Network | Exclude: Irrelevant to the research topic |
| [246] | HUANG H-H, STUBBS B, CHEN L-J, et al. | The Effect Of Physical Activity On Sleep Disturbance In Various Populations: A Scoping Review Of Randomized Clinical Trials | Exclude: Irrelevant to the research topic |
| [247] | HUANG P Y, CHOU P Y, LIN C H. | A Transformer-Based Object Relationship Finder For Object Status Analysis | Exclude: Irrelevant to the research topic |
| [248] | HUANG Q, LIAO Y D. | High Precision Recognition Method Of Basketball Dribbling Posture Based On Lightweight RFID Mobile Authentication Protocol | Exclude: Irrelevant to the research topic |
| [249] | HUANG Y, CHEN H. | A Basketball Player Technical Analysis Framework Based On Decision Tree Optimized Recursive Neural Network | Exclude: Irrelevant to the research topic |
| [250] | HUANG Y, HUANG S, WANG Y, et al. | A Novel Lower Extremity Non-Contact Injury Risk Prediction Model Based On Multimodal Fusion And Interpretable Machine Learning | Exclude: Irrelevant to the research topic |
| [251] | HUANG Y, LI C, BAI Z, et al. | The Impact Of Sport-Specific Physical Fitness Change Patterns On Lower Limb Non-Contact Injury Risk In Youth Female Basketball Players: A Pilot Study Based On Field Testing And Machine Learning | Exclude: Irrelevant to the research topic |
| [252] | HUANG Y, ZHANG J, AN L, et al. | Synergetic Effect Of Mxene/Mos2 Heterostructure And Gradient Multilayer For Highly Sensitive Flexible Piezoelectric Sensor | Exclude: Irrelevant to the research topic |
| [253] | HUBáČEK O, ŠOUREK G, ŽELEZNý F. | Exploiting Sports-Betting Market Using Machine Learning | Exclude: Irrelevant to the research topic |
| [254] | HUO D. | Evaluation Of The Value Of Basketball Players Based On Wireless Network And Improved Bayesian Algorithm | Exclude: Irrelevant to the research topic |
| [255] | HUO X, IEEE. | The Method Of Predicting Basketball Risks From A Personalized Perspective | Exclude: Irrelevant to the research topic |
| [256] | ISTASSE M, MOREAU J, DE VLEESCHOUWER C. | Associative Embedding For Team Discrimination | Exclude: Irrelevant to the research topic |
| [257] | ISTASSE M, SOMERS V, ELANCHELIYAN P, et al. | Deepsportradar-V2: A Multi-Sport Computer Vision Dataset For Sport Understandings | Exclude: Irrelevant to the research topic |
| [258] | IVANKOVIC Z, MARKOSKI B, IVKOVIC M, et al. | Adaboost In Basketball Player Identification | Exclude: Irrelevant to the research topic |
| [259] | IVANKOVIC Z, RACKOVIC M, MARKOSKI B, et al. | Appliance Of Neural Networks In Basketball Scouting | Exclude: Irrelevant to the research topic |
| [260] | IVANKOVIĆ Z, RACKOVIĆ M, MARKOSKI B, et al. | Analysis Of Basketball Games Using Neural Networks | Exclude: Irrelevant to the research topic |
| [261] | IVANOVIC B, SCHMERLING E, LEUNG K, et al. | Generative Modeling Of Multimodal Multi-Human Behavior | Exclude: Irrelevant to the research topic |
| [262] | IVANOVSKY L, MATVEEV D, KHRYASHCHEV V, et al. | Detection And Tracking Of Sport Players On Videodata Using Deep Learning Methods | Exclude: Irrelevant to the research topic |
| [263] | JAIN A, JAIN S, PANCINOVIA N M, et al. | A Non-Linear Approach To Predict The Salary Of NBA Athletes Using Machine Learning Technique | Exclude: Irrelevant to the research topic |
| [264] | JANG S-Y, 구승환, 김현수. | A Comparison Study On The Prediction Models For The Professional Basketball Games | Exclude: Publication outside the 2019-2024 period |
| [265] | JAUHIAINEN S, KAUPPI J-P, LEPPANEN M, et al. | New Machine Learning Approach For Detection Of Injury Risk Factors In Young Team Sport Athletes | Exclude: Irrelevant to the research topic |
| [266] | JAVADPOUR L, BLAKESLEE J, KHAZAELI M, et al. | Optimizing The Best Play In Basketball Using Deep Learning | Exclude: Irrelevant to the research topic |
| [267] | JAVED AWAN M, MOHD RAHIM M S, SALIM N, et al. | Efficient Detection Of Knee Anterior Cruciate Ligament From Magnetic Resonance Imaging Using Deep Learning Approach | Exclude: Irrelevant to the research topic |
| [268] | JI B, LI J, IEEE. | NBA All-Star Lineup Prediction Based On Neural Networks | Exclude: Irrelevant to the research topic |
| [269] | JI N, ZHAO S, LIN Q, et al. | NBA Basketball Video Summarization For News Report Via Hierarchical-Grained Deep Reinforcement Learning | Exclude: Irrelevant to the research topic |
| [270] | JI R. | Research On Basketball Shooting Action Based On Image Feature Extraction And Machine Learning | Exclude: Irrelevant to the research topic |
| [271] | JIA H, REN C, HU Y, et al. | Mastering Basketball With Deep Reinforcement Learning: An Integrated Curriculum Training Approach | Exclude: Irrelevant to the research topic |
| [272] | JIA J, CHEN H. | The Rating Of Basketball Players’ Competitive Performance Based On RBF-EVA Method | Exclude: Irrelevant to the research topic |
| [273] | JIA X. | Research On College Sports Training Based On Computer Virtual Reality Technology | Exclude: Irrelevant to the research topic |
| [274] | JIANG H. | Secondary Data Analysis System Development And Design For Basketball Coaches | Exclude: Irrelevant to the research topic |
| [275] | JIANG H. | Research On Basketball Goal Recognition Based On Image Processing And Improved Algorithm | Exclude: Irrelevant to the research topic |
| [276] | JIANG H, QIU T, DEEPA THILAK K. | Application Of Deep Learning Method In Automatic Collection And Processing Of Video Surveillance Data For Basketball Sports Prediction | Exclude: Irrelevant to the research topic |
| [277] | JIANG L, LU W. | Sports Competition Tactical Analysis Model Of Cross-Modal Transfer Learning Intelligent Robot Based On Swin Transformer And CLIP | Exclude: Irrelevant to the research topic |
| [278] | JIANG L, ZHANG D. | Deep Learning Algorithm Based Wearable Device For Basketball Stance Recognition In Basketball | Exclude: Irrelevant to the research topic |
| [279] | JIANG R. | Research On Visualization System Of Competition Information Under Computer Artificial Intelligence Technology | Exclude: Irrelevant to the research topic |
| [280] | JING L, KITAHAMA K, YAMATA M, et al. | Basketball Foul Model And Judgment System Proposal | Exclude: Irrelevant to the research topic |
| [281] | JOKL E, MCCLELLAN J T, WILLIAMS W C, et al. | Congenital Anomaly Of Left Coronary Artery In Young Athletes | Exclude: Irrelevant to the research topic |
| [282] | JOSHI K, SUGANTHI K. | Anterior Cruciate Ligament Tear Detection Based On Convolutional Neural Network And Generative Adversarial Neural Network | Exclude: Irrelevant to the research topic |
| [283] | JOSHI K, TRIPATHI V, BOSE C, et al. | Robust Sports Image Classification Using Inceptionv3 And Neural Networks | Exclude: Irrelevant to the research topic |
| [284] | JUG M, PERŠ J, DEŽMAN B, et al. | Trajectory Based Assessment Of Coordinated Human Activity | Exclude: Irrelevant to the research topic |
| [285] | KANDHWAY K. | Dynamic Outcome Prediction Of An NBA Match | Include |
| [286] | KANG D. | Optimization Method Of Real-Time Basketball Defensive Strategy Based On Motion Tracking Technology And Deep Learning | Exclude: Irrelevant to the research topic |
| [287] | KATRIS C. | Investigation Of FIBA World Cup 2019: Evidence Using Advanced Statistical Analysis And Quantitative Tools | Exclude: Irrelevant to the research topic |
| [288] | KAUR H, JAIN S. | Machine Learning Approaches To Predict Basketball Game Outcome | Exclude: Publication outside the 2019-2024 period |
| [289] | KAYHAN V O, WATKINS A. | Predicting The Point Spread In Professional Basketball In Real Time: A Data Snapshot Approach | Include |
| [290] | KE Y, BIAN R, CHANDRA R. | A Unified Machine Learning Framework For Basketball Team Roster Construction: NBA And WNBA | Exclude: Irrelevant to the research topic |
| [291] | KEKEZ I, CUKUSIC M, JADRIC M. | Data Mining Approach For Business Value Analysis In Basketball | Exclude: Irrelevant to the research topic |
| [292] | KEMPE M, GRUNZ A, MEMMERT D. | Detecting Tactical Patterns In Basketball: Comparison Of Merge Self-Organising Maps And Dynamic Controlled Neural Networks | Exclude: Irrelevant to the research topic |
| [293] | KHOBDEH S B, YAMAGHANI M R, SARESHKEH S K. | Basketball Action Recognition Based On The Combination Of YOLO And A Deep Fuzzy LSTM Network | Exclude: Irrelevant to the research topic |
| [294] | KIM H. | Study On The Prediction Of The Number Of Spectators And It’s Factors In Pro Sports By Machine Learning Method | Exclude: Irrelevant to the research topic |
| [295] | KIM J, LEE H. | Adaptive Human-Machine Evaluation Framework Using Stochastic Gradient Descent-Based Reinforcement Learning For Dynamic Competing Network | Exclude: Irrelevant to the research topic |
| [296] | KIM J W, MAGNUSEN M, JEONG S. | March Madness Prediction: Different Machine Learning Approaches With Non-Box Score Statistics | Exclude: Focus on non-professional basketball leagues |
| [297] | KIPP K, KIELY M T, GIORDANELLI M D, et al. | Biomechanical Determinants Of The Reactive Strength Index During Drop Jumps | Exclude: Irrelevant to the research topic |
| [298] | KLUTSE E K, NUAMAH-AMOABENG S, LYU H, et al. | Dismantling Hate: Understanding Hate Speech Trends Against Nba Athletes | Exclude: Irrelevant to the research topic |
| [299] | KOJIMA K. | Development Of Wheelchair Basketball Player Tracker Using LED And Omni-Camera | Exclude: Irrelevant to the research topic |
| [300] | KOJIMA K, IEEE. | CNN-Based Marker Extraction For Wheelchair Basketball Player Tracking System Using LED And Omnidirectional Camera | Exclude: Irrelevant to the research topic |
| [301] | KOSHIDA S, DEGUCHI T, MIYASHITA K, et al. | The Common Mechanisms Of Anterior Cruciate Ligament Injuries In Judo: A Retrospective Analysis | Exclude: Irrelevant to the research topic |
| [302] | KREITZER R, DENNIS R, WASSERMAN S, et al. | Golf And Gameforge: Innovative Analytics For Recommender Systems | Exclude: Irrelevant to the research topic |
| [303] | KRISHNA MOHAN C, YEGNANARAYANA B. | Edge-Based Sports Video Classification Using HMM | Exclude: Irrelevant to the research topic |
| [304] | KRISHNAN N J, SURESH R, VASHISHT S, et al. | Prediction Of National Basketball Association Games Using Machine Learning With Integrating Advanced Statistics | Include |
| [305] | KUHLMAN N, MIN C H. | Analysis And Classification Of Basketball Shooting Form Using Wearable Sensor Systems | Exclude: Irrelevant to the research topic |
| [306] | KUMAR V, WORSLEY M. | Scratch For Sports: Athletic Drills As A Platform For Experiencing, Understanding, And Developing AI-Driven Apps | Exclude: Irrelevant to the research topic |
| [307] | KUO W Y, KUO C H, SUN S W, et al. | Machine Learning-Based Behavior Recognition System For A Basketball Player Using Multiple Kinect Cameras | Exclude: Irrelevant to the research topic |
| [308] | KUO Y-K, SHEN W-T, SHIH T-S, et al. | How Organizational Behavior, Artificial Intelligence, And Marketing Techniques In Sports Clubs Influence New Product Development | Exclude: Irrelevant to the research topic |
| [309] | KUZNETSOV A, SAVCHENKO A V. | A New Sport Teams Logo Dataset For Detection Tasks | Exclude: Irrelevant to the research topic |
| [310] | KYOUNG D, SUNG Y. | Transformer Decoder-Based Enhanced Exploration Method To Alleviate Initial Exploration Problems In Reinforcement Learning | Exclude: Irrelevant to the research topic |
| [311] | LAI X, SHI W, LIU L, et al. | A Study On The Motion Recognition Of Basketball Players Based On Unit Gesture Decomposition | Exclude: Irrelevant to the research topic |
| [312] | LALA D, KAWAHARA T. | Managing Dialog And Joint Actions For Virtual Basketball Teammates | Exclude: Irrelevant to the research topic |
| [313] | LALA D, LI Y, KAWAHARA T. | Utterance Behavior Of Users While Playing Basketball With A Virtual Teammate | Exclude: Irrelevant to the research topic |
| [314] | LALA D, NISHIDA T. | Modeling Communicative Virtual Agents Based On Joint Activity Theory | Exclude: Irrelevant to the research topic |
| [315] | LALA D, NISHIDA T. | A Data-Driven Passing Interaction Model For Embodied Basketball Agents | Exclude: Irrelevant to the research topic |
| [316] | LALA D, NITSCHKE C, NISHIDA T. | User Perceptions Of Communicative And Task-Competent Agents In A Virtual Basketball Game | Exclude: Irrelevant to the research topic |
| [317] | LAM M W Y. | Tlgprob: Two-Layer Gaussian Process Regression Model For Winning Probability Calculation In Two-Team Sports | Exclude: Publication outside the 2019-2024 period |
| [318] | LAM M W Y. | One-Match-Ahead Forecasting In Two-Team Sports With Stacked Bayesian Regressions | Exclude: Publication outside the 2019-2024 period |
| [319] | LAMPIS T, IOANNIS N, VASILIOS V, et al. | Predictions Of European Basketball Match Results With Machine Learning Algorithms | Include |
| [320] | LARA J A, LIZCANO D, DE LA PEñA D, et al. | Data Mining In Stabilometry: Application To Patient Balance Study For Sports Talent Mapping | Exclude: Irrelevant to the research topic |
| [321] | LEICHSENRING Y E, PARPINELLI R S, BALDO F. | A Method To Identify Defensive Assignments In Team-Based Invasion Sports Using Spatiotemporal Trajectories | Exclude: Irrelevant to the research topic |
| [322] | LEICHT A S, GOMEZ M A, WOODS C T. | Team Performance Indicators Explain Outcome During Women's Basketball Matches At The Olympic Games | Exclude: Irrelevant to the research topic |
| [323] | LEICHT A S, GóMEZ M A, WOODS C T. | Explaining Match Outcome During The Men's Basketball Tournament At The Olympic Games | Exclude: Irrelevant to the research topic |
| [324] | LI B, WANG L, FENG H. | Intelligent Correction Method Of Shooting Action Based On Computer Vision | Exclude: Irrelevant to the research topic |
| [325] | LI C. | Predict The Neural Network Mathematical Model Of Basketball Team Scores Based On Improved BP Algorithm | Exclude: Publication outside the 2019-2024 period |
| [326] | LI C. | Design Of Basketball Network Teaching Information System Based On Web | Exclude: Irrelevant to the research topic |
| [327] | LI C, SU Y, QI J, et al. | Using GAN To Generate Sport News From Live Game Stats | Exclude: Irrelevant to the research topic |
| [328] | LI D. | Cultivation Of College Basketball Referee Ability Using Infrared Thermal Imaging Target Recognition And Tracking System | Exclude: Irrelevant to the research topic |
| [329] | LI H. | Basketball Action Behavior Recognition Algorithm Based On Dynamic Recognition Technology | Exclude: Irrelevant to the research topic |
| [330] | LI H. | Automatic AI Detection Technology Of Foul Shot Scanning In Simulated Basketball Match Under The Background Of AI | Exclude: Irrelevant to the research topic |
| [331] | LI H, ZHANG M. | Artificial Intelligence And Neural Network-Based Shooting Accuracy Prediction Analysis In Basketball | Exclude: Irrelevant to the research topic |
| [332] | LI J, GU D, WAGNER N, et al. | Research On Basketball Players' Action Recognition Based On Interactive System And Machine Learning | Exclude: Irrelevant to the research topic |
| [333] | LI K. | Study On The Segmentation Method Of The Improved Deeplabv3+Algorithm In The Basketball Scene | Exclude: Irrelevant to the research topic |
| [334] | LI N. | Basketball Culture Event Detection Based On Comprehensive Integrated Method | Exclude: Irrelevant to the research topic |
| [335] | LI Q. | Video Analysis Method Of Basketball Training Based On Deep Learning In The Context Of Online Gaming | Exclude: Irrelevant to the research topic |
| [336] | LI Q, CHEN L, WANG H, et al. | Wonderful Clips Of Playing Basketball: A Database For Localizing Wonderful Actions | Exclude: Irrelevant to the research topic |
| [337] | LI S, ASSOC COMP M. | Revisiting The Correlation Of Basketball Stats And Match Outcome Prediction | Include |
| [338] | LI S, MA X. | An Analysis Method Of Exercise Load In Physical Training Based On Radial Basis Neural Network Model | Exclude: Irrelevant to the research topic |
| [339] | LI S, ZHANG W. | Evaluation Method Of Basketball Teaching And Training Effect Based On Wearable Device | Exclude: Irrelevant to the research topic |
| [340] | LI W. | Deep Learning Based Sports Video Classification Research | Exclude: Irrelevant to the research topic |
| [341] | LI W, WU Y, LIAN B, et al. | Deep Learning Algorithm-Based Target Detection And Fine Localization Of Technical Features In Basketball | Exclude: Irrelevant to the research topic |
| [342] | LI W-K. | Research On Application Of Artificial Intelligence In Basketball | Exclude: Irrelevant to the research topic |
| [343] | LI X. | Shoot Rate In Basketball Game Based On Metal Sensor | Exclude: Irrelevant to the research topic |
| [344] | LI X. | National Basketball Association Most Valuable Player Prediction Based On Machine Learning Methods | Exclude: Irrelevant to the research topic |
| [345] | LI X, LUO R, UL ISLAM F. | Tracking And Detection Of Basketball Movements Using Multi-Feature Data Fusion And Hybrid YOLO-T2LSTM Network | Exclude: Irrelevant to the research topic |
| [346] | LI Y. | The Application Of CAI Based On Expert Decision Support System(EDSS) In Youth Basketball Skill Training | Exclude: Irrelevant to the research topic |
| [347] | LI Y, CAO J, WANG Y. | Implementation Of Intelligent Question Answering System Based On Basketball Knowledge Graph | Exclude: Irrelevant to the research topic |
| [348] | LI Z, SUN H, LI J. | Lightweight Gesture Recognition Algorithm For Basketball Referee | Exclude: Irrelevant to the research topic |
| [349] | LI Z, SUN H, LI J. | Design Of Real-Time Basketball Referee Gesture Recognition System Based On Loss Weighting | Exclude: Irrelevant to the research topic |
| [350] | LIAN C, MA R, WANG X, et al. | ANN-Enhanced Iot Wristband For Recognition Of Player Identity And Shot Types Based On Basketball Shooting Motion Analysis | Exclude: Irrelevant to the research topic |
| [351] | LIANG Q, MEI L, WU W, et al. | Automatic Basketball Detection In Sport Video Based On R-FCN And Soft-NMS | Exclude: Irrelevant to the research topic |
| [352] | LIANG X. | A Video Images-Aware Knowledge Extraction Method For Intelligent Healthcare Management Of Basketball Players | Exclude: Irrelevant to the research topic |
| [353] | LIN C, CHEN W. | Research On Basketball Robot Recognition And Localization Based On Mobilenet-SSD And Multi-Sensor | Exclude: Irrelevant to the research topic |
| [354] | LIN C C, CHEN V, YU C C, et al. | A Schema Of Decision Support System To Determine Basketball Zone Defense Patterns Using A Fuzzy Expert System | Exclude: Irrelevant to the research topic |
| [355] | LIN C C, LIN Y C, LIN Y J, et al. | Determining Basketball Defense Types By Grey Correlation Analysis And Fuzzy Expert Systemlin | Exclude: Irrelevant to the research topic |
| [356] | LIN C H, TSAI M Y, CHOU P Y. | A Lightweight Fine-Grained Action Recognition Network For Basketball Foul Detection | Exclude: Irrelevant to the research topic |
| [357] | LIN G. | Application Of Fast Behavior Detection Based On Image Optical Processing In Basketball Training Image Recognition | Exclude: Irrelevant to the research topic |
| [358] | LIN J, SUN L, SONG J. | Research On The Application Of Artificial Intelligence Video Feedback System In College Basketball Shooting Teaching | Exclude: Irrelevant to the research topic |
| [359] | LIN S H, CHEN M Y, CHIANG H S. | Forecasting Results Of Sport Events Through Deep Learning | Exclude: Publication outside the 2019-2024 period |
| [360] | LINDERMAN S W, JOHNSON M J, MILLER A C, et al. | Bayesian Learning And Inference In Recurrent Switching Linear Dynamical Systems | Exclude: Irrelevant to the research topic |
| [361] | LINGYUN G, KAIWANG L, QINGYUN F, et al. | Optimizing Object Detectors With Knowledge Distillation For On-Board Earth Observation | Exclude: Irrelevant to the research topic |
| [362] | LIO J, OKADA T. | An Experimental Trial Of A Novel Ticketing System Using Biometrics | Exclude: Irrelevant to the research topic |
| [363] | LITOIU A, SCASSELLATI B. | Personalized Instruction Of Physical Skills With A Social Robot | Exclude: Irrelevant to the research topic |
| [364] | LIU F, ZHANG W. | Basketball Motion Posture Recognition Based On Recurrent Deep Learning Model | Exclude: Irrelevant to the research topic |
| [365] | LIU G, LIU Y. | Application Of Wearable Devices Based On Deep Learning Algorithm In Basketball Posture Recognition | Exclude: Irrelevant to the research topic |
| [366] | LIU H, BHANU B. | JEDE: Universal Jersey Number Detector For Sports | Exclude: Irrelevant to the research topic |
| [367] | LIU J. | Convolutional Neural Network-Based Human Movement Recognition Algorithm In Sports Analysis | Exclude: Irrelevant to the research topic |
| [368] | LIU J. | Motion Action Analysis At Basketball Sports Scene Based On Image Processing | Exclude: Irrelevant to the research topic |
| [369] | LIU L. | Objects Detection Toward Complicated High Remote Basketball Sports By Leveraging Deep CNN Architecture | Exclude: Irrelevant to the research topic |
| [370] | LIU L, HODGINS J. | Learning Basketball Dribbling Skills Using Trajectory Optimization And Deep Reinforcement Learning | Exclude: Irrelevant to the research topic |
| [371] | LIU M J, IEEE I. | A CAT Software Development In Physical Culture Field | Exclude: Irrelevant to the research topic |
| [372] | LIU Q. | Investigation And Analysis Of Current Situation Of High School Campus Basketball In Huining City Based On Big Data Analysis | Exclude: Irrelevant to the research topic |
| [373] | LIU R, LIU Z, LIU S. | Recognition Of Basketball Player's Shooting Action Based On The Convolutional Neural Network | Exclude: Irrelevant to the research topic |
| [374] | LIU T, ZHENG Q, TIAN L. | The Influence Of Computer Network Technology Using Digital Technology On The Quality Of Physical Education In Colleges Under Complex Scenes | Exclude: Irrelevant to the research topic |
| [375] | LIU W, LIU J, GU X, et al. | Deep Learning Based Intelligent Basketball Arena With Energy Image | Exclude: Irrelevant to the research topic |
| [376] | LIU W, YAN C C, LIU J, et al. | Deep Learning Based Basketball Video Analysis For Intelligent Arena Application | Exclude: Irrelevant to the research topic |
| [377] | LIU Y. | Teaching Effect And Improvement Model Of College Basketball Sports Based On Big Data Analysis | Exclude: Irrelevant to the research topic |
| [378] | LIU Y. | Prediction For NCAA Championship | Exclude: Focus on non-professional basketball leagues |
| [379] | LIU Y, DONG A, YU J, et al. | A Wi-Fi Sensing Method For Complex Continuous Human Activities Based On CNN-Bigru | Exclude: Irrelevant to the research topic |
| [380] | LIU Y, LI S, YU J, et al. | Wifi Sensing For Drastic Activity Recognition With CNN-Bilstm Architecture | Exclude: Irrelevant to the research topic |
| [381] | LIU Y, SCHULTE O, LI C. | Model Trees For Identifying Exceptional Players In The NHL And NBA Drafts | Exclude: Irrelevant to the research topic |
| [382] | LIU Y, WANG L, TANG Y, et al. | Judgment Of Athlete Action Safety In Sports Competition Based On LSTM Recurrent Neural Network Algorithm | Exclude: Irrelevant to the research topic |
| [383] | LIU Y, WANG S. | Application Research Of Basketball Physical Training Based On Artificial Intelligence | Exclude: Irrelevant to the research topic |
| [384] | LIU Y, WU H. | A People Counting Method Based On Universities’ Surveillance Videos And Its Application On Classroom Query(Including Subseries Lecture Notes In Artificial Intelligence And Lecture Notes In Bioinformatics) | Exclude: Irrelevant to the research topic |
| [385] | LIU Y, ZHAO Y. | Application Of Near-Infrared Spectral Imaging And Artificial Intelligence Classification In Basketball Motion Image Recognition | Exclude: Irrelevant to the research topic |
| [386] | LIU Z. | Application Of Artificial Intelligence Technology In Basketball Games | Exclude: Irrelevant to the research topic |
| [387] | LIU Z. | Sustainable Strategy For Online Physical Education Teaching Using Resnet34 And Big Data | Exclude: Irrelevant to the research topic |
| [388] | LIU Z, CAO J, YUAN Z. | Maneuvering Target Tracking Using Adaptive Models In A Particle Filter | Exclude: Irrelevant to the research topic |
| [389] | LIU Z, WANG X. | Action Recognition For Sports Combined Training Based On Wearable Sensor Technology And SVM Prediction | Exclude: Irrelevant to the research topic |
| [390] | LIU Z, WANG Y, CHEN T. | Audio Feature Extraction And Analysis For Scene Segmentation And Classification | Exclude: Irrelevant to the research topic |
| [391] | LIU Z, ZHAO J. | Data Analysis Of The Development Status Of Basketball National Fitness Based On Fog Computing | Exclude: Irrelevant to the research topic |
| [392] | LONG T, OUTERLEYS J, YEUNG T, et al. | Predicting Ankle And Knee Sagittal Kinematics And Kinetics Using An Ankle-Mounted Inertial Sensor | Exclude: Irrelevant to the research topic |
| [393] | LOPES T J A, SIMIC M, ALVES D D S, et al. | Physical Performance Measures Of Flexibility, Hip Strength, Lower Limb Power, And Trunk Endurance In Healthy Navy Cadets: Normative Data And Differences Between Sex And Limb Dominance | Exclude: Irrelevant to the research topic |
| [394] | LOPEZ M J, MATTHEWS G J. | Building An NCAA Men's Basketball Predictive Model And Quantifying Its Success | Exclude: Irrelevant to the research topic |
| [395] | LOU H, WANG G. | Study Of Athletic Trauma In College Physical Education | Exclude: Irrelevant to the research topic |
| [396] | LU C-J, LEE T-S, WANG C-C, et al. | Improving Sports Outcome Prediction Process Using Integrating Adaptive Weighted Features And Machine Learning Techniques | Include |
| [397] | LU G. | Evaluation Model Of Young Basketball Players' Physical Quality And Basic Technique Based On RBF Neural Network | Exclude: Irrelevant to the research topic |
| [398] | LU J. | Basketball Motion Recognition Model Analysis Based On Perspective Invariant Geometric Features In Skeleton Data Extraction | Exclude: Irrelevant to the research topic |
| [399] | LU J, CHEN Y, ZHU Y. | Prediction Of Future NBA Games' Point Difference: A Statistical Modeling Approach | Include |
| [400] | LU J, WU X, CAO S, et al. | An Implementation Of Actor-Critic Algorithm On Spiking Neural Network Using Temporal Coding Method | Exclude: Irrelevant to the research topic |
| [401] | LU K, CHEN J, LITTLE J J, et al. | Light Cascaded Convolutional Neural Networks For Accurate Player Detection | Exclude: Irrelevant to the research topic |
| [402] | LU K, CHEN J, LITTLE J J, et al. | Lightweight Convolutional Neural Networks For Player Detection And Classification | Exclude: Irrelevant to the research topic |
| [403] | LU Q, LI J, LIN Z, et al. | Decoding The Fashion Trend Of Sports Shoes With Empowered Computer Vision | Exclude: Irrelevant to the research topic |
| [404] | LU Y, PAREEK A, LAVOIE-GAGNE O Z, et al. | Machine Learning For Predicting Lower Extremity Muscle Strain In National Basketball Association Athletes | Exclude: Irrelevant to the research topic |
| [405] | LU Y, WANG Z, TANG Z, et al. | Target Localization With Drones Using Mobile Cnns | Exclude: Irrelevant to the research topic |
| [406] | LU Z. | Algorithm Of Basketball Posture Motion Feature Extraction Based On Image Processing Technology | Exclude: Irrelevant to the research topic |
| [407] | LUJAN R C, SOSA K F C, GOMEZ O R O, et al. | The Upper And Lower Limb Bilateral Morphological Asymmetry Index In University Basketball Players | Exclude: Irrelevant to the research topic |
| [408] | MA B, WANG Y, LI Z. | Application Of Data Mining In Basketball Statistics | Include |
| [409] | MA C, FAN J, YAO J, et al. | NPU RGB Plus D Dataset And A Feature-Enhanced LSTM-DGCN Method For Action Recognition Of Basketball Players | Exclude: Irrelevant to the research topic |
| [410] | MA D. | On-The-Spot Decision-Making System Of Basketball Game Based On Data Mining Algorithm | Exclude: Irrelevant to the research topic |
| [411] | MA J, LI W. | Efficient Image Segmentation Of Cardiac Conditions After Basketball Using A Deep Neural Network | Exclude: Irrelevant to the research topic |
| [412] | MA K. | Research On Basketball Teaching Network Course Resource Recommendation Method Based On Deep Learning Algorithm | Exclude: Irrelevant to the research topic |
| [413] | MA L. | Research On Key Technologies Of Basketball Object Segmentation And Tracking Based On Image Analysis | Exclude: Irrelevant to the research topic |
| [414] | MA R, YAN D, PENG H, et al. | Basketball Movements Recognition Using A Wrist Wearable Inertial Measurement Unit | Exclude: Irrelevant to the research topic |
| [415] | MA Y, LU Z. | An Adaptive Algorithm For Motion Recognition Of Throwing Arm On Account Of Neural Network Technology | Exclude: Irrelevant to the research topic |
| [416] | MACALPINE P, STONE P. | Evaluating Ad Hoc Teamwork Performance In Drop-In Player Challenges | Exclude: Irrelevant to the research topic |
| [417] | MAEDA G, KOç O, MORIMOTO J. | Phase Portraits As Movement Primitives For Fast Humanoid Robot Control | Exclude: Irrelevant to the research topic |
| [418] | MAGLO A, ORCESI A, QUOC-CUONG P, et al. | Kalicalib: A Framework For Basketball Court Registration | Exclude: Irrelevant to the research topic |
| [419] | MAHAJAN Y, PINNAMRAJU J, BURNS J L, et al. | Using Machine Learning Approaches To Identify Exercise Activities From A Triple-Synchronous Biomedical Sensor | Exclude: Irrelevant to the research topic |
| [420] | MAHMOOD Z, DAUD A, ABBASI R A. | Using Machine Learning Techniques For Rising Star Prediction In Basketball | Exclude: Irrelevant to the research topic |
| [421] | MAKAROV I, PETROV S. | On The Impact Of Computer Vision Algorithms On Sport Training Automation | Exclude: Irrelevant to the research topic |
| [422] | MANASSES I. | A Study Of The Behavior Of Romanian And Foreign Basketball Players In The U-Mobitelco Team In FIBA Eurochallenge Cup Games | Exclude: Irrelevant to the research topic |
| [423] | MANGIAROTTI M, FERRISE F, GRAZIOSI S, et al. | A Wearable Device To Detect In Real-Time Bimanual Gestures Of Basketball Players During Training Sessions | Exclude: Irrelevant to the research topic |
| [424] | MANN R, JEPSON A D. | Detection And Classification Of Motion Boundaries | Exclude: Irrelevant to the research topic |
| [425] | MANN R, JEPSON A D, EL-MARAGHI T. | Trajectory Segmentation Using Dynamic Programming | Exclude: Irrelevant to the research topic |
| [426] | MARKOSKI B, PECEV P, RATGEBER L, et al. | A New Approach To Decision Making In Basketball - BBFBR Program | Exclude: Irrelevant to the research topic |
| [427] | MARKOSKI B, PECEV P, RATGEBER L, et al. | Appliance Of Neural Networks In Basketball - Basketball Board For Basketball Referees | Exclude: Irrelevant to the research topic |
| [428] | MARQUéS-JIMéNEZ D, RAYA-GONZáLEZ J, SáNCHEZ-DíAZ S, et al. | A Random Forest Clustering To Explore The Influence Of Physical Fitness Level Of Youth Basketball Players On Match-Related Physical Performance | Exclude: Irrelevant to the research topic |
| [429] | MATEOS L A. | SPXS Sports Picks Expert System | Exclude: Irrelevant to the research topic |
| [430] | MATOS FLORES S E. | Semi-Automatic Basketball Jump Shot Annotation Using Multi-View Activity Recognition And Deep Learning | Exclude: Irrelevant to the research topic |
| [431] | MATSUURA K, TANIOKA H, GOTODA N, et al. | Self-Studying Environment With Imagery Rehearsal For A Ball-Carrier In Basketball(IMCOM) | Exclude: Irrelevant to the research topic |
| [432] | MAYMIN P. | Using Scouting Reports Text To Predict Ncaa → Nba Performance | Exclude: Irrelevant to the research topic |
| [433] | MAYMIN P Z. | The Automated General Manager: Can An Algorithmic System For Drafts, Trades, And Free Agency Outperform Human Front Offices? | Exclude: Irrelevant to the research topic |
| [434] | MAYMIN P Z. | Wage Against The Machine: A Generalized Deep-Learning Market Test Of Dataset Value | Exclude: Irrelevant to the research topic |
| [435] | MENG F-L, YANG T. | A Recognition Method Of Basketball's Shooting Trajectory Based On Transfer Learning | Exclude: Irrelevant to the research topic |
| [436] | MENG H. | Deep Learning Algorithm And Video Image Processing-Based Basketball Training System | Exclude: Irrelevant to the research topic |
| [437] | MENG H. | Basketball Training System Based On 3D Motion Intelligent Recognition Technology | Exclude: Irrelevant to the research topic |
| [438] | MENG Q. | Psychological Analysis Of Athletes During Basketball Games From The Perspective Of Deep Learning | Exclude: Irrelevant to the research topic |
| [439] | MENG X H, SHI H Y, SHANG W H. | Analysis Of Basketball Technical Movements Based On Human-Computer Interaction With Deep Learning | Exclude: Irrelevant to the research topic |
| [440] | METULINI R. | Spatio-Temporal Movements In Team Sports: A Visualization Approach Using Motion Charts | Exclude: Irrelevant to the research topic |
| [441] | METULINI R, GNECCO G. | Measuring Players' Importance In Basketball Using The Generalized Shapley Value | Exclude: Irrelevant to the research topic |
| [442] | MGAYA G B, LIU H, ZHANG B. | A Survey On Applications Of Modern Deep Learning Techniques In Team Sports Analytics | Exclude: Irrelevant to the research topic |
| [443] | MI Q, XUE D. | A Sound-Based Video Clipping Framework Toward Sports Scenes | Exclude: Irrelevant to the research topic |
| [444] | MIGLIORATI M. | Detecting Drivers Of Basketball Successful Games: An Exploratory Study With Machine Learning Algorithms | Include |
| [445] | MIHATA L C, BEUTLER A I, BODEN B P. | Comparing The Incidence Of Anterior Cruciate Ligament Injury In Collegiate Lacrosse, Soccer, And Basketball Players: Implications For Anterior Cruciate Ligament Mechanism And Prevention | Exclude: Irrelevant to the research topic |
| [446] | MILDEBRATH D, KNIGHT W, SCHAEFER A. | Optimal Jersey Retirement In The National Basketball Association | Exclude: Irrelevant to the research topic |
| [447] | MILI R, DAS N R, TANDON A, et al. | Pose Recognition In Cricket Using Keypoints | Exclude: Irrelevant to the research topic |
| [448] | MILJKOVIĆ D, GAJIĆ L, KOVAČEVIĆ A, et al. | The Use Of Data Mining For Basketball Matches Outcomes Prediction | Exclude: Publication outside the 2019-2024 period |
| [449] | MILLER A, BOMN L, ADAMS R, et al. | Factorized Point Process Intensities: A Spatial Analysis Of Professional Basketball | Exclude: Irrelevant to the research topic |
| [450] | MIMURA T, NAKADA Y. | Enhancements Of Pass Play Quantification Method With Geometric Features Of Formations | Exclude: Irrelevant to the research topic |
| [451] | MITCHELL M. | Assessment Of Patellar Tendons Over The Course Of A Collegiate Men's And Women's Basketball Season Using Gray-Scale Ultrasound And Shear-Wave Elastography | Exclude: Irrelevant to the research topic |
| [452] | MOHAN C K, YEGNANARAYANA B. | Classification Of Sport Videos Using Edge-Based Features And Autoassociative Neural Network Models | Exclude: Irrelevant to the research topic |
| [453] | MONEZI L A, CALDERANI JUNIOR A, MERCADANTE L A, et al. | A Video-Based Framework For Automatic 3D Localization Of Multiple Basketball Players: A Combinatorial Optimization Approach | Exclude: Irrelevant to the research topic |
| [454] | MORARIU V I, HARWOOD D, DAVIS L S. | Tracking People's Hands And Feet Using Mixed Network AND/OR Search | Exclude: Irrelevant to the research topic |
| [455] | MORICE A H P, HADATINE I, MAROT J, et al. | A Framework For Optimizing AI-Based Virtual Reality: A Use Case In Sport Sciences | Exclude: Irrelevant to the research topic |
| [456] | MU G, LI T. | Video-Based Metric Learning Framework For Basketball Skill Assessment | Exclude: Irrelevant to the research topic |
| [457] | MüLLER O, CARON M, DöRING M, et al. | PIVOT: A Parsimonious End-To-End Learning Framework For Valuing Player Actions In Handball Using Tracking Data | Exclude: Irrelevant to the research topic |
| [458] | MURATA A. | Cognitive Biases In Game Momentum, Winning Strategy, And Jinx In Baseball | Exclude: Irrelevant to the research topic |
| [459] | MUSHHOOD AFSAR M, SAQIB S. | Virtual Reality Game Based Interaction With Exergaming Using Wearable Sensors | Exclude: Irrelevant to the research topic |
| [460] | NA N A. | Deep Similarity Learning For Sports Team Ranking | Exclude: Absence of specific empirical data |
| [461] | NADY A, HEMAYED E. | Player Identification In Different Sports | Exclude: Irrelevant to the research topic |
| [462] | NAGARAJAN G, THYAGHARAJAN K K. | Rule-Based Semantic Content Extraction In Image Using Fuzzy Ontology | Exclude: Irrelevant to the research topic |
| [463] | NAIK B T, HASHMI M F, BOKDE N D. | A Comprehensive Review Of Computer Vision In Sports: Open Issues, Future Trends And Research Directions | Exclude: Irrelevant to the research topic |
| [464] | NAKAI M, TSUNODA Y, HAYASHI H, et al. | Prediction Of Basketball Free Throw Shooting By Openpose | Exclude: Irrelevant to the research topic |
| [465] | NAN Z. | Impact On The Pace Of The Game Of Basketball Analysis Of The Factors | Exclude: Irrelevant to the research topic |
| [466] | NEIMAN T, LOEWENSTEIN Y. | Reinforcement Learning In Professional Basketball Players | Exclude: Irrelevant to the research topic |
| [467] | NEPPALLI S, JONES B, MCMAHAN W, et al. | Octarm - A Soft Robotic Manipulator | Exclude: Irrelevant to the research topic |
| [468] | NGUYEN N, MA B, HU J. | Predicting National Basketball Association Players Performance And Popularity: A Data Mining Approach | Exclude: Irrelevant to the research topic |
| [469] | NGUYEN N H, NGUYEN D T A, MA B, et al. | The Application Of Machine Learning And Deep Learning In Sport: Predicting NBA Players’ Performance And Popularity | Exclude: Irrelevant to the research topic |
| [470] | NI Y-Z, NO L S. | Comparison Of Prediction Performance Of Machine Learning Classification Model Using 2022 FIBA Men's Basketball Asian Cup Match Results | Exclude: Were not written in English |
| [471] | NICO S, PAN T Y, PRAWIRO H, et al. | Offensive Tactics Recognition In Broadcast Basketball Videos Based On 2D Camera View Player Heatmaps | Exclude: Irrelevant to the research topic |
| [472] | NOEL J T P, PRADO DA FONSECA V, SOARES A. | A Comprehensive Data Pipeline For Comparing The Effects Of Momentum On Sports Leagues | Exclude: Lack of predicting game outcomes |
| [473] | OKIMOTO T, HIRAYAMA K. | U12 Basketball League Scheduling Problem Based On Minimizing The Total Traveling Distance And The Number Of Breaks | Exclude: Irrelevant to the research topic |
| [474] | OLEA C, OMER G, CARTER J, et al. | Analysis Of Deep Learning Action Recognition For Basketball Shot Type Identification | Exclude: Irrelevant to the research topic |
| [475] | OLUWASEUN O T, SCHERRER D. | Space Science Education And Outreach In Developing Countries: Challenges, Solutions, Innovation, And Creativity | Exclude: Irrelevant to the research topic |
| [476] | OPROESCU M, MIHAILA I, BIZON N, et al. | Detection And Analysis Of Motricity And Somato-Functional Indices Of 3X3 Basketball Players To Customize Sports Training | Exclude: Irrelevant to the research topic |
| [477] | ORTIZ-PADILLA V E, RAMIREZ-MORENO M A, PRESBITERO-ESPINOSA G, et al. | Survey On Video-Based Biomechanics And Biometry Tools For Fracture And Injury Assessment In Sports | Exclude: Irrelevant to the research topic |
| [478] | OSKEN C, ONAY C. | Predicting The Winning Team In Basketball: A Novel Approach | Include |
| [479] | ÖTTING M. | Predicting Play Calls In The National Football League Using Hidden Markov Models | Exclude: Irrelevant to the research topic |
| [480] | OUGHALI M S, BAHLOUL M, EL RAHMAN S A, et al. | Analysis Of NBA Players And Shot Prediction Using Random Forest And Xgboost Models | Exclude: Irrelevant to the research topic |
| [481] | OVED N, FEDER A, REICHART R. | Predicting In-Game Actions From Interviews Of NBA Players | Exclude: Irrelevant to the research topic |
| [482] | ÖZBALTA E, YAVUZ M, KAYA T. | National Basketball Association Player Salary Prediction Using Supervised Machine Learning Methods | Exclude: Irrelevant to the research topic |
| [483] | OZKAN I A. | A Novel Basketball Result Prediction Model Using A Concurrent Neuro-Fuzzy System | Include |
| [484] | PACHOLSKI L. | The Intelligent Support Programming Of The Fans' Emergency Evacuation Of The Sports Stadium Event | Exclude: Irrelevant to the research topic |
| [485] | PAI P-F, CHANGLIAO L-H, LIN K-P. | Analyzing Basketball Games By A Support Vector Machines With Decision Tree Model | Exclude: Publication outside the 2019-2024 period |
| [486] | PAN T-Y, CHANG C-Y, TSAI W-L, et al. | Multisensor-Based 3D Gesture Recognition For A Decision-Making Training System | Exclude: Irrelevant to the research topic |
| [487] | PAN T-Y, CHANG C-Y, TSAI W-L, et al. | Orsnet: A Hybrid Neural Network For Official Sports Referee Signal Recognition | Exclude: Irrelevant to the research topic |
| [488] | PAN T-Y, TSAI W-L, CHANG C-Y, et al. | A Hierarchical Hand Gesture Recognition Framework For Sports Referee Training-Based EMG And Accelerometer Sensors | Exclude: Irrelevant to the research topic |
| [489] | PAN Z, LIU L, LI X, et al. | The Influence Of Experience On Neuromuscular Control Of The Body When Cutting At Different Angles | Exclude: Irrelevant to the research topic |
| [490] | PANDYA M, PILLAI A, RUPANI H. | Segregating And Recognizing Human Actions From Video Footages Using Lrcn Technique | Exclude: Irrelevant to the research topic |
| [491] | PANG W, FAN X, GU Y, et al. | Chinese Unknown Words Extraction Based On Word-Level Characteristics | Exclude: Irrelevant to the research topic |
| [492] | PAPAGEORGIOU G, SARLIS V, TJORTJIS C. | Unsupervised Learning In NBA Injury Recovery: Advanced Data Mining To Decode Recovery Durations And Economic Impacts | Exclude: Irrelevant to the research topic |
| [493] | PAPAGEORGIOU G, SARLIS V, TJORTJIS C. | An Innovative Method For Accurate NBA Player Performance Forecasting And Line-Up Optimization In Daily Fantasy Sports | Exclude: Irrelevant to the research topic |
| [494] | PARK J-H, CHO K, IEEE. | Extraction Of Visual Information In Basketball Broadcasting Video For Event Segmentation System | Exclude: Irrelevant to the research topic |
| [495] | PATROT A, HARISH H, SHAMBBAVI B, et al. | Nba Game Prediction Using Machine Learning Algorithm | Include |
| [496] | PECEV P, RACKOVIĆ M. | LTR - MDTS Structure - A Structure For Multiple Dependent Time Series Prediction | Exclude: Irrelevant to the research topic |
| [497] | PECEV P, RACKOVIĆ M, IVKOVIĆ M. | A System For Deductive Prediction And Analysis Of Movement Of Basketball Referees | Exclude: Irrelevant to the research topic |
| [498] | PELECHRINIS K, PAPALEXAKIS E, ACM. | Athlytics: Winning In Sports With Data | Exclude: Irrelevant to the research topic |
| [499] | PELECHRINIS K, WINSTON W. | The Hot Hand In The Wild | Exclude: Irrelevant to the research topic |
| [500] | PENA J M, MENASALVAS E, MUELAS S, et al. | Soft Computing For Content Generation: Trading Market In A Basketball Management Video Game | Exclude: Irrelevant to the research topic |
| [501] | PENG K, ZHAO Y, SHA X, et al. | Accurate Recognition Of Volleyball Motion Based On Fusion Of MEMS Inertial Measurement Unit And Video Analytic | Exclude: Irrelevant to the research topic |
| [502] | PENG M, ZHANG Z, ZHOU Q, et al. | Basketball Footwork Recognition Using Smart Insoles Integrated With Multiple Sensors | Exclude: Irrelevant to the research topic |
| [503] | PENG Y, ZHAO Z. | Lighting Design Of Basketball Hall Based On Neural Network | Exclude: Irrelevant to the research topic |
| [504] | PERSE M, KRISTAN M, PERS J, et al. | Analysis Of Multi-Agent Activity Using Petri Nets | Exclude: Irrelevant to the research topic |
| [505] | PFEIFFER K A, LISEE C, WESTGATE B S, et al. | Using Accelerometers To Detect Activity Type In A Sport Setting: Challenges With Using Multiple Types Of Conventional Machine Learning Approaches | Exclude: Irrelevant to the research topic |
| [506] | PI Y-L, WU X-H, WANG F-J, et al. | Motor Skill Learning Induces Brain Network Plasticity: A Diffusion-Tensor Imaging Study | Exclude: Irrelevant to the research topic |
| [507] | PION J, SEGERS V, STAUTEMAS J, et al. | Position-Specific Performance Profiles, Using Predictive Classification Models In Senior Basketball | Exclude: Irrelevant to the research topic |
| [508] | PLOCH C, THOMAS D M, ADAMS B, et al. | Prediction Of Cadet Basic Training Physical Performance Outcomes | Exclude: Irrelevant to the research topic |
| [509] | POBAR M, IVASIC-KOS M. | Active Player Detection In Handball Scenes Based On Activity Measures | Exclude: Irrelevant to the research topic |
| [510] | POPP N, DU J, SHAPIRO S L, et al. | Using Artificial Intelligence To Detect The Relationship Between Social Media Sentiment And Season Ticket Purchases | Exclude: Irrelevant to the research topic |
| [511] | PORTAZ M, MANJARRES A, SANTOS O C, et al. | Towards Human-Centric Psychomotor Recommender Systems | Exclude: Irrelevant to the research topic |
| [512] | PROKSCH S, REEVES M, GEE K, et al. | Recurrence Quantification Analysis Of Crowd Sound Dynamics | Exclude: Irrelevant to the research topic |
| [513] | QI M, WANG Y, LI A, et al. | Sports Video Captioning Via Attentive Motion Representation And Group Relationship Modeling | Exclude: Irrelevant to the research topic |
| [514] | QI M, WANG Y, LI A, et al. | Sports Video Captioning By Attentive Motion Representation Based Hierarchical Recurrent Neural Networks | Exclude: Irrelevant to the research topic |
| [515] | QIAN T, YAO P, GUO M, et al. | Using Adaptive Object Model To Basketball Tracking Algorithm And Simulation | Exclude: Irrelevant to the research topic |
| [516] | QIAO R, REN J, JI Y. | Research On The Optimization Of Basketball Teaching Content Of Physical Education Major In Artificial Intelligence Colleges And Universities | Exclude: Irrelevant to the research topic |
| [517] | QIN W. | Application Analysis Of Basketball Training System Based On Personalized Recommendation Systems | Exclude: Irrelevant to the research topic |
| [518] | QIU C, SU C, LIU X, et al. | A Study Of Feature Construction Based On Least Squares And RBF Neural Networks In Sports Training Behaviour Prediction | Exclude: Irrelevant to the research topic |
| [519] | QIU Y-K, HONG F-T, LI W-H, et al. | Learning Relation Models To Detect Important People In Still Images | Exclude: Irrelevant to the research topic |
| [520] | RAAB M. | T-ECHO: Model Of Decision Making To Explain Behaviour In Experiments And Simulations Under Time Pressure | Exclude: Irrelevant to the research topic |
| [521] | RADLO S J. | Effects Of Biofeedback And Imagery On Learning In A Competitive Environment | Exclude: Irrelevant to the research topic |
| [522] | RAHATABAD F N, MAGHOOLI K, BALAN T R. | Relationship Between Muscle Synergies And Skills Of Basketball Players | Exclude: Irrelevant to the research topic |
| [523] | RAMANATHAN V, HUANG J, ABU-EL-HAIJA S, et al. | Detecting Events And Key Actors In Multi-Person Videos | Exclude: Irrelevant to the research topic |
| [524] | RANGEL W, UGRINOWITSCH C, LAMAS L. | Basketball Players' Versatility: Assessing The Diversity Of Tactical Roles | Exclude: Irrelevant to the research topic |
| [525] | RATGEBER L, MARKOSKI B, PECEV P, et al. | Comparative Review Of Statistical Parameters For Men's And Women's Basketball Leagues In Serbia | Exclude: Irrelevant to the research topic |
| [526] | RAZMAH M, SENTHIL G A, PRABHA R, et al. | LSTM Method For Human Activity Recognition Of Video Using PSO Algorithm | Exclude: Irrelevant to the research topic |
| [527] | REBUFFEL C, SCOUTHEETEN G, SOULIER L, et al. | Capturing Entity Hierarchy In Data-To-Text Generative Models | Exclude: Irrelevant to the research topic |
| [528] | ROCHA DA SILVA J V, RODRIGUES P C. | The Three Eras Of The NBA Regular Seasons: Historical Trend And Success Factors | Exclude: Irrelevant to the research topic |
| [529] | RODAS G, OSABA L, ARTETA D, et al. | Genomic Prediction Of Tendinopathy Risk In Elite Team Sports | Exclude: Irrelevant to the research topic |
| [530] | ROMAN-RIVERA L R, PEDRAZA-ORTEGA J C, ACEVES-FERNANDEZ M A, et al. | A Robust Sphere Detection In A Realsense Point Cloud By USING Z-Score And RANSAC | Exclude: Irrelevant to the research topic |
| [531] | RONG J, CHEN Y, YANG J. | CNN-LSTM Hybrid Model For Kinematic Feature Analysis And Parabolic Radian Prediction In Basketball Videos | Exclude: Irrelevant to the research topic |
| [532] | RONG J, CUI L. | Research On The Decision-Making System Of Basketball Game Of Virtual Human And Application Prospect | Exclude: Irrelevant to the research topic |
| [533] | ROSENSTOCK S, CHAMBERS R, LEE A, et al. | Self-Efficacy And Response-Efficacy: Critical Components Of Sexual And Reproductive Health Interventions Targeting Condom Use Intention Among American Indian Adolescents | Exclude: Irrelevant to the research topic |
| [534] | RUSSO G, MANZARI A, CUOZZO B, et al. | Learning And Knowledge Transfer By Humans And Digital Platforms: Which Tools Best Support The Decision-Making Process? | Exclude: Irrelevant to the research topic |
| [535] | SABAREESWARI T C, HANUSHA A, VAMSI P, et al. | Estimating Of Noise Patterns By Satellite Image Using ELBP And SVM | Exclude: Irrelevant to the research topic |
| [536] | SAE-HYUNG K. | Comparison Of Estimating Method Of Victory And Defeat Through Analyzing Records Of Korean Pro-Basketball | Exclude: Irrelevant to the research topic |
| [537] | SAHA U, MAHMUD M S, SHIMU S, et al. | The Corporeality Of Infotainment On Fans Feedback Towards Sports Comment Employing Convolutional Long-Short Term Neural Network | Exclude: Irrelevant to the research topic |
| [538] | ŞAHIN M, UçAR M. | Prediction Of Sports Attendance: A Comparative Analysis | Exclude: Irrelevant to the research topic |
| [539] | SANGUINO BAUTISTE F J, BRUNNER D, KOCH J, et al. | The Big Three: A Practical Framework For Designing Decision Support Systems In Sports And An Application For Basketbal | Exclude: Focus on non-professional basketball leagues |
| [540] | SANJAY H S, BHARGAVI S, DINESH P A. | Auditory Temporal Resolution Based Psychophysical Evaluation Of Healthy Individuals Exposed To Desired And Undesired Conditioning | Exclude: Irrelevant to the research topic |
| [541] | SANJAY H S, HIREMATH B V, PRITHVI B S, et al. | Machine Learning Based Assessment Of Auditory Threshold Perception In Human Beings | Exclude: Irrelevant to the research topic |
| [542] | SARLIS V, CHATZIILIAS V, TJORTJIS C, et al. | A Data Science Approach Analysing The Impact Of Injuries On Basketball Player And Team Performance | Exclude: Irrelevant to the research topic |
| [543] | SARLIS V, PAPAGEORGIOU G, TJORTJIS C. | Sports Analytics And Text Mining NBA Data To Assess Recovery From Injuries And Their Economic Impact | Exclude: Irrelevant to the research topic |
| [544] | SARLIS V, TJORTJIS C. | Sports Analytics - Evaluation Of Basketball Players And Team Performance | Exclude: Irrelevant to the research topic |
| [545] | SATHYAN A, HARRISON H S, KIEFER A W, et al. | Genetic Fuzzy System For Anticipating Athlete Decision Making In Virtual Reality | Exclude: Irrelevant to the research topic |
| [546] | SAWCZUK T, JONES B, WELCH M, et al. | Prediction Of Upper Respiratory Illness Using Salivary Immunoglobulin A In Youth Athletes | Exclude: Irrelevant to the research topic |
| [547] | SCHMIDT A. | Movement Pattern Recognition In Basketball Free-Throw Shooting | Exclude: Irrelevant to the research topic |
| [548] | SCHRODT F, LAYHER G, NEUMANN H, et al. | Embodied Learning Of A Generative Neural Model For Biological Motion Perception And Inference | Exclude: Irrelevant to the research topic |
| [549] | SCHULC A, LEITE C B G, CSáKVáRI M, et al. | Identifying Anterior Cruciate Ligament Injuries Through Automated Video Analysis Of In-Game Motion Patterns | Exclude: Irrelevant to the research topic |
| [550] | SCOFANO L, SAMPIERI A, RE G, et al. | About Latent Roles In Forecasting Players In Team Sports | Exclude: Irrelevant to the research topic |
| [551] | SENBEL S, SHARMA S, RAVAL M S, et al. | Impact Of Sleep And Training On Game Performance And Injury In Division-1 Women's Basketball Amidst The Pandemic | Exclude: Irrelevant to the research topic |
| [552] | SENOCAK A, OH T-H, KIM J, et al. | Part-Based Player Identification Using Deep Convolutional Representation And Multi-Scale Pooling | Exclude: Irrelevant to the research topic |
| [553] | SHAN H, WANG S, TONG J, et al. | Multi-Scale Optical Remote Sensing Image Target Detection Based On Enhanced Small Target Features | Exclude: Irrelevant to the research topic |
| [554] | SHANKAR A, RAJASEKARAN G V, HENDRICKS J, et al. | Are Sports Awards About Sports? Using AI To Find The Answer | Exclude: Irrelevant to the research topic |
| [555] | SHAO Q. | Virtual Reality And ANN-Based Three-Dimensional Tactical Training Model For Football Players | Exclude: Irrelevant to the research topic |
| [556] | SHEN J, ZHAO Y, LIU J K, et al. | Recognizing Scoring In Basketball Game From AER Sequence By Spiking Neural Networks | Exclude: Irrelevant to the research topic |
| [557] | SHEN Z, YANG Y. | Real-Time Regulation Model Of Physical Fitness Training Intensity Based On Wavelet Recursive Fuzzy Neural Network | Exclude: Irrelevant to the research topic |
| [558] | SHENG C, LIAN C, PANG H. | A Practical Study Of Basketball Teaching Reform In Colleges And Universities Based On Big Data | Exclude: Irrelevant to the research topic |
| [559] | SHI H, XING X, LI D. | An Extended Semantic Framework For Theme-Interview | Exclude: Irrelevant to the research topic |
| [560] | SHI J, CHAN A, BERTASIUS G. | Egocentric Basketball Motion Planning From A Single First-Person Image | Exclude: Irrelevant to the research topic |
| [561] | SHI J, WANG X, CHENG F, et al. | Recreation Behavior Of Village Residents In Zhejiang Province | Exclude: Irrelevant to the research topic |
| [562] | SHI S, ZHOU Q F, PENG M, et al. | Utilize Smart Insole To Recognize Basketball Motions | Exclude: Irrelevant to the research topic |
| [563] | SHI W, WANG H, LOU X. | Multi-Modal Graph Reasoning For Structured Video Text Extraction | Exclude: Irrelevant to the research topic |
| [564] | SHI X, WANG Q, WANG C, et al. | An AI-Based Curling Game System For Winter Olympics | Exclude: Irrelevant to the research topic |
| [565] | SHI Y, HU Z. | Recognizing The Take-Off Action Pattern Of Basketball Players Based On Fuzzy Neural Network System | Exclude: Irrelevant to the research topic |
| [566] | SHI Z, LI M, WANG M, et al. | Npipvis: A Visualization System Involving NBA Visual Analysis And Integrated Learning Model Prediction | Exclude: Irrelevant to the research topic |
| [567] | SHIH A, SAWHNEY A, KONDIC J, et al. | On The Critical Role Of Conventions In Adaptive Human-AI Collaboration | Exclude: Irrelevant to the research topic |
| [568] | SHITRIT H B, BERCLAZ J, FLEURET F, et al. | Multi-Commodity Network Flow For Tracking Multiple People | Exclude: Irrelevant to the research topic |
| [569] | SHUM H P H, HO E S L, JIANG Y, et al. | Real-Time Posture Reconstruction For Microsoft Kinect | Exclude: Irrelevant to the research topic |
| [570] | SICILIA A, PELECHRINIS K, GOLDSBERRY K, et al. | Deephoops: Evaluating Micro-Actions In Basketball Using Deep Feature Representations Of Spatio-Temporal Data | Exclude: Irrelevant to the research topic |
| [571] | SIGARI M H, SURESHJANI S A, SOLTANIAN-ZADEH H. | Sport Video Classification Using An Ensemble Classifier | Exclude: Irrelevant to the research topic |
| [572] | SIKKA D, RAJESWARI D. | Basketball Win Percentage Prediction Using Ensemble-Based Machine Learning | Include |
| [573] | SILVA A, FERRAZ R, BRANQUINHO L, et al. | Effects Of Applying A Multivariate Training Program On Physical Fitness And Tactical Performance In A Team Sport Taught During Physical Education Classes | Exclude: Irrelevant to the research topic |
| [574] | ŠIRMENIS J, LUKOŠEVIČIUS M. | Tracking Basketball Shots - Preliminary Results | Exclude: Irrelevant to the research topic |
| [575] | SKINNER B, GUY S J. | A Method For Using Player Tracking Data In Basketball To Learn Player Skills And Predict Team Performance | Exclude: Irrelevant to the research topic |
| [576] | SMIRNOV E F, IVANOV N S, ZAVGORODNII M, et al. | Recognition Of The Basketball Players Position Using Live Cameras | Exclude: Irrelevant to the research topic |
| [577] | SOLIMAN G, EL-NABAWY A A, MISBAH A, et al. | Predicting All Star Player In The National Basketball Association Using Random Forest | Exclude: Irrelevant to the research topic |
| [578] | SONG X, FAN L. | Human Posture Recognition And Estimation Method Based On 3D Multiview Basketball Sports Dataset | Exclude: Irrelevant to the research topic |
| [579] | SONG X, FAN L. | Pattern Recognition Characteristics And Neural Mechanism Of Basketball Players' Dribbling Tactics Based On Artificial Intelligence And Deep Learning | Exclude: Irrelevant to the research topic |
| [580] | SOUTH C, EGROS E. | Forecasting College Football Game Outcomes Using Modern Modeling Techniques | Exclude: Irrelevant to the research topic |
| [581] | SPURLOCK S, SOUVENIR R. | Dynamic Subset Selection For Multi-Camera Tracking | Exclude: Irrelevant to the research topic |
| [582] | SRINIVASAN R, BALASUBRAMANIAN V, VIDYASAGAR A. | A Supervised Learning Model To Identify The Star Potential Of A Basketball Player | Exclude: Irrelevant to the research topic |
| [583] | STANKEVICH L, TROTSKY D. | On-Line Agent Teamwork Training Using Immunological Network Model | Exclude: Irrelevant to the research topic |
| [584] | STARKE S, ZHAO Y, KOMURA T, et al. | Local Motion Phases For Learning Multi-Contact Character Movements | Exclude: Irrelevant to the research topic |
| [585] | STEPHANOS D K, HUSARI G, BENNETT B T, et al. | Machine Learning Predictive Analytics For Player Movement Prediction In NBA: Applications, Opportunities, And Challenges | Exclude: Irrelevant to the research topic |
| [586] | STOICOVICIU A, PREDESCU T. | Program Vizând Îmbunătăţirea Forţei La Studenţii Din Învăţământul Superior Prin Mijloacele Specifice Jocului De Baschet | Exclude: Irrelevant to the research topic |
| [587] | STOJANOVIĆ E, JAKOVLJEVIĆ V, ZBOŘILOVá V, et al. | A Comparison Of Bone Mass And Body Composition According To Playing Positions And Playing Roles In Male Adolescent Basketball Players | Exclude: Irrelevant to the research topic |
| [588] | SU F, CHEN M. | Basketball Players' Score Prediction Using Artificial Intelligence Technology Via The Internet Of Things | Include |
| [589] | SU K. | Machine Learning-Based Study Of The Influence Factors On The Wins Of NBA Teams | Exclude: Irrelevant to the research topic |
| [590] | SU S, HONG J P, SHI J, et al. | Predicting Behaviors Of Basketball Players From First Person Videos | Exclude: Irrelevant to the research topic |
| [591] | SUBASHKA RAMESH S S, HASSAN N, KHANDELWAL A, et al. | Analytics And Machine Learning Approaches To Generate Insights For Different Sports | Exclude: Irrelevant to the research topic |
| [592] | SUDAKOV V A, BELOZEROV I A, PRUDKOVA E S. | Reinforcement Machine Learning Model For Sports Infrastructure Development Planning | Exclude: Irrelevant to the research topic |
| [593] | SUN C, MURPHY K, KARLSSON P, et al. | Stochastic Prediction Of Multi-Agent Interactions From Partial Observations | Exclude: Irrelevant to the research topic |
| [594] | SUN H, WANG Y, WANG Y. | Application Of Unsupervised Migration Method Based On Deep Learning Model In Basketball Training | Exclude: Irrelevant to the research topic |
| [595] | SUN S, SUN Y, GOTO M, et al. | Motor Learning Based On Presentation Of A Tentative Goal | Exclude: Irrelevant to the research topic |
| [596] | SUN Y. | A Numerical Computer Simulation System For The Influence Of Physical Fitness Training Loads On Basketball Players | Exclude: Irrelevant to the research topic |
| [597] | SUN Y. | Research On Computer Artificial Intelligence Simulating Basketball Player Tactics System | Exclude: Irrelevant to the research topic |
| [598] | SUN Z, HEROLD F, CAI K, et al. | Prediction Of Outcomes In Mini-Basketball Training Program For Preschool Children With Autism Using Machine Learning Models | Exclude: Irrelevant to the research topic |
| [599] | SUN Z, YUAN Y, DONG X, et al. | Supervised Machine Learning: A New Method To Predict The Outcomes Following Exercise Intervention In Children With Autism Spectrum Disorder | Exclude: Irrelevant to the research topic |
| [600] | TABER C B, SHARMA S, RAVAL M S, et al. | A Holistic Approach To Performance Prediction In Collegiate Athletics: Player, Team, And Conference Perspectives | Exclude: Irrelevant to the research topic |
| [601] | TABORRI J, MOLINARO L, SANTOSPAGNUOLO A, et al. | A Machine-Learning Approach To Measure The Anterior Cruciate Ligament Injury Risk In Female Basketball Players | Exclude: Irrelevant to the research topic |
| [602] | TAKAGI S, NAKADA Y. | Formation Analysis Method For Team Sports Using Deep Graph Convolutional Neural Network With Geometric Formation Features As Input | Exclude: Irrelevant to the research topic |
| [603] | TAKEUCHI R, TAMEI T, IEEE. | Automatic Generation And Inferring Semantic Structure Of Verbal Instructions For A Motor Task | Exclude: Irrelevant to the research topic |
| [604] | TAN G, LIU D, WANG M, et al. | Learning To Discretely Compose Reasoning Module Networks For Video Captioning | Exclude: Irrelevant to the research topic |
| [605] | TAN X-Y, PI Y-L, WANG J, et al. | Morphological And Functional Differences Between Athletes And Novices In Cortical Neuronal Networks | Exclude: Irrelevant to the research topic |
| [606] | TANAKA T, UCHIYAMA A, YAMAGUCHI H. | ML-Based Individual Contribution Assessment Of Basketball Players From Their Trajectories | Exclude: Irrelevant to the research topic |
| [607] | TANG B, GUAN W. | CNN Multi-Position Wearable Sensor Human Activity Recognition Used In Basketball Training | Exclude: Irrelevant to the research topic |
| [608] | TANG H. | Honey On Basketball Players' Physical Recovery And Nutritional Supplement | Exclude: Irrelevant to the research topic |
| [609] | TANG S. | The Application Of Computer-Assisted Instruction To Basketball Technique And Tactics Teaching | Exclude: Irrelevant to the research topic |
| [610] | TARASHIMA S. | Sflnet: Direct Sports Field Localization Via CNN-Based Regression | Exclude: Irrelevant to the research topic |
| [611] | TARASHIMA S. | Sports Field Recognition Using Deep Multi-Task Learning | Exclude: Irrelevant to the research topic |
| [612] | TAYAL M A, DESHMUKH M, PANGAVE V, et al. | VMLHST: Development Of An Efficient Novel Virtual Reality ML Framework With Haptic Feedbacks For Improving Sports Training Scenarios | Exclude: Irrelevant to the research topic |
| [613] | TEGICHO B E, CHESTNUT M D, WEBB D, et al. | Basketball Shot Analysis Based On Goal Assembly Disturbance | Exclude: Irrelevant to the research topic |
| [614] | TEKET O M, YETIK I S. | A Fast Deep Learning Based Approach For Basketball Video Analysis | Exclude: Irrelevant to the research topic |
| [615] | THABTAH F, ZHANG L, ABDELHAMID N. | NBA Game Result Prediction Using Feature Analysis And Machine Learning | Include |
| [616] | THAKUR S, KARTHIK R. | End Of Game Shot Selection For Individual Players In The NBA | Exclude: Irrelevant to the research topic |
| [617] | THOMSON C, REITER E, SUNDARARAJAN B. | Evaluating Factual Accuracy In Complex Data-To-Text | Exclude: Irrelevant to the research topic |
| [618] | TIAN C, DE SILVA V, CAINE M, et al. | Use Of Machine Learning To Automate The Identification Of Basketball Strategies Using Whole Team Player Tracking Data | Exclude: Irrelevant to the research topic |
| [619] | TIAN T, GELLER J, CHUN S A. | Improving Web Search Results For Homonyms By Suggesting Completions From An Ontology | Exclude: Irrelevant to the research topic |
| [620] | TIAN Y, RUAN Q, AN G, et al. | Context And Locality Constrained Linear Coding For Human Action Recognition | Exclude: Irrelevant to the research topic |
| [621] | TINGEY L, MULLANY B, CHAMBERS R, et al. | The Respecting The Circle Of Life Trial For American Indian Adolescents: Rationale, Design, Methods, And Baseline Characteristics | Exclude: Irrelevant to the research topic |
| [622] | TINGEY L, MULLANY B, CHAMBERS R, et al. | Respecting The Circle Of Life: One Year Outcomes From A Randomized Controlled Comparison Of An HIV Risk Reduction Intervention For American Indian Adolescents | Exclude: Irrelevant to the research topic |
| [623] | TJONDRONEGORO D, CHEN Y P P. | Towards Universal And Statistical-Driven Heuristics For Automatic Classification Of Sports Video Events | Exclude: Irrelevant to the research topic |
| [624] | TJONDRONEGORO D, CHEN Y P P. | Using Decision-Tree To Automatically Construct Learned-Heuristics For Events Classification In Sports Video | Exclude: Irrelevant to the research topic |
| [625] | TJONDRONEGORO D W, CHEN Y-P P. | Knowledge-Discounted Event Detection In Sports Video | Exclude: Irrelevant to the research topic |
| [626] | TOMAS J P Q, LUCERO K I, AJERO C J P, et al. | Comparative Study On Model Skill Of ERT And LSTM In Classifying Proper Or Improper Execution Of Free Throw, Jump Shot, And Layup Basketball Maneuvers | Exclude: Irrelevant to the research topic |
| [627] | TORRALBA E M. | Sports Ed 3.5: Establishing The Value Of Data-Driven Sports Development Programs For Universities Through Machine Learning Models | Exclude: Irrelevant to the research topic |
| [628] | TRAWIŃSKI K. | A Fuzzy Classification System For Prediction Of The Results Of The Basketball Games | Exclude: Publication outside the 2019-2024 period |
| [629] | TRNINIĆ S, DIZDAR D, DEZMAN B. | Pragmatic Validity Of The Combined Model Of Expert System For Assessment And Analysis Of The Actual Quality Overall Structure Of Basketball Players | Exclude: Irrelevant to the research topic |
| [630] | TROST S G, ZHENG Y, WONG W K. | Machine Learning For Activity Recognition: Hip Versus Wrist Data | Exclude: Irrelevant to the research topic |
| [631] | TRUONG C, RUFFINO C, CROGNIER A, et al. | Error-Based And Reinforcement Learning In Basketball Free Throw Shooting | Exclude: Irrelevant to the research topic |
| [632] | TSAI T-Y, LIN Y-Y, JENG S-K, et al. | End-To-End Key-Player-Based Group Activity Recognition Network Applied To Basketball Offensive Tactic Identification In Limited Data Scenarios | Exclude: Irrelevant to the research topic |
| [633] | TSAI T-Y, LIN Y-Y, LIAO H-Y M, et al. | Recognizing Offensive Tactics In Broadcast Basketball Videos Via Key Player Detection | Exclude: Irrelevant to the research topic |
| [634] | TSAI W L, SU L W, KO T Y, et al. | Feasibility Study On Using AI And VR For Decision-Making Training Of Basketball Players | Exclude: Irrelevant to the research topic |
| [635] | TSOLAKIS N, VRYZAS N, DIMOULAS C, et al. | An Ontology-Based Framework For Sports Media Data Interpretation | Exclude: Irrelevant to the research topic |
| [636] | TUDOSOIU D, OPROESCU M, MIHAILA I. | Hardware And Software Solution For Measure The Vertical Jump In The Basketball Game | Exclude: Irrelevant to the research topic |
| [637] | TUEMER A E, KOCER S. | Prediction Of Team League's Rankings In Volleyball By Artificial Neural Network Method | Exclude: Irrelevant to the research topic |
| [638] | TUYLS K, OMIDSHAFIEI S, MULLER P, et al. | Game Plan: What AI Can Do For Football, And What Football Can Do For AI | Exclude: Irrelevant to the research topic |
| [639] | ULAS E. | Examination Of National Basketball Association (NBA) Team Values Based On Dynamic Linear Mixed Models | Exclude: Irrelevant to the research topic |
| [640] | VALLANCE E, SUTTON-CHARANI N, GUYOT P, et al. | Predictive Modeling Of The Ratings Of Perceived Exertion During Training And Competition In Professional Soccer Players | Exclude: Irrelevant to the research topic |
| [641] | VAN ROY M, ROBBERECHTS P, YANG W-C, et al. | A Markov Framework For Learning And Reasoning About Strategies In Professional Soccer | Exclude: Irrelevant to the research topic |
| [642] | VAN ZANDYCKE G, DE VLEESCHOUWER C, ACM. | Real-Time CNN-Based Segmentation Architecture For Ball Detection In A Single View Setup | Exclude: Irrelevant to the research topic |
| [643] | VAN ZANDYCKE G, DE VLEESCHOUWER C, IEEE. | 3d Ball Localization From A Single Calibrated Image | Exclude: Irrelevant to the research topic |
| [644] | VAN ZANDYCKE G, SOMERS V, ISTASSE M, et al. | Deepsportradar-V1: Computer Vision Dataset For Sports Understanding With High Quality Annotations | Exclude: Irrelevant to the research topic |
| [645] | VASUDEVAN V, SELLAPPA GOUNDER M. | Advances In Sports Video Summarization – A Review Based On Cricket Videos | Exclude: Irrelevant to the research topic |
| [646] | VEALE J P, PEARCE A J, CARLSON J S. | The Yo-Yo Intermittent Recovery Test (Level 1) To Discriminate Elite Junior Australian Football Players | Exclude: Irrelevant to the research topic |
| [647] | VICKERS J N, VANDERVIES B, KOHUT C, et al. | Quiet Eye Training Improves Accuracy In Basketball Field Goal Shooting | Exclude: Irrelevant to the research topic |
| [648] | VRACAR P. | Applying Vector Embeddings For Basketball Modeling | Exclude: Irrelevant to the research topic |
| [649] | VRAČAR P. | Uporaba Vektorskih Vložitev Pri Modeliranju Poteka Košarkarske Tekme | Exclude: Irrelevant to the research topic |
| [650] | VRACAR P, STRUMBELJ E, KONONENKO I. | Modeling Basketball Play-By-Play Data | Exclude: Irrelevant to the research topic |
| [651] | VROONEN R, DECROOS T, VAN HAAREN J, et al. | Predicting The Potential Of Professional Soccer Players | Exclude: Irrelevant to the research topic |
| [652] | WANG C S, WANG C C, CHANG T R, et al. | Feature Recognition And Shape Design In Sneakers | Exclude: Irrelevant to the research topic |
| [653] | WANG G, LIU J, LO W, et al. | Learning Multiple Instance Deep Quality Representation For Robust Object Tracking | Exclude: Irrelevant to the research topic |
| [654] | WANG H. | Basketball Sports Posture Recognition Based On Neural Computing And Visual Sensor | Exclude: Irrelevant to the research topic |
| [655] | WANG H. | Multimodal Audio-Visual Robot Fusing 3D CNN And CRNN For Player Behavior Recognition And Prediction In Basketball Matches | Exclude: Irrelevant to the research topic |
| [656] | WANG J, SONG X. | Development Status And Influencing Factors Of Competitive Basketball Management System Under The Background Of Deep Learning | Exclude: Irrelevant to the research topic |
| [657] | WANG J-G, CAI K-L, LIU Z-M, et al. | Effects Of Mini-Basketball Training Program On Executive Functions And Core Symptoms Among Preschool Children With Autism Spectrum Disorders | Exclude: Irrelevant to the research topic |
| [658] | WANG K, WEI Z. | YOLO V4 With Hybrid Dilated Convolution Attention Module For Object Detection In The Aerial Dataset | Exclude: Irrelevant to the research topic |
| [659] | WANG L, WEI X, YUAN G. | Research On The Design Of Assistant Basketball Teaching System Based On Big Data | Exclude: Irrelevant to the research topic |
| [660] | WANG M, ZHOU L, ZHANG C. | Upper Limb Movement Trajectory Recognition Of Basketball Players Based On Machine Learning | Exclude: Irrelevant to the research topic |
| [661] | WANG P, BAEK S. | Early Warning Of Basketball Injury Risk Based On Attribute Reduction Algorithm | Exclude: Irrelevant to the research topic |
| [662] | WANG P, GAO W. | Image Detection And Basketball Training Performance Simulation Based On Improved Machine Learning | Exclude: Irrelevant to the research topic |
| [663] | WANG Q, LIU B, LIN J. | Crowd Understanding And Analysis | Exclude: Irrelevant to the research topic |
| [664] | WANG Q, REN J, YANG H, et al. | Intelligent System For Training And Assessment Of Basketball Referee In Sports Event Using Intelligent Sensor | Exclude: Irrelevant to the research topic |
| [665] | WANG Q, TAO B, HAN F, et al. | Extraction And Recognition Method Of Basketball Players' Dynamic Human Actions Based On Deep Learning | Exclude: Irrelevant to the research topic |
| [666] | WANG Q, WEI X W. | Risk Assessment For Basketball Match Based On Fuzzy Analytic Hierarchy Process | Exclude: Irrelevant to the research topic |
| [667] | WANG S-W, HSIEH W-W. | Performance Analysis Of Basketball Referees By Machine Learning Techniques | Exclude: Irrelevant to the research topic |
| [668] | WANG T, SHI C. | Basketball Motion Video Target Tracking Algorithm Based On Improved Gray Neural Network | Exclude: Irrelevant to the research topic |
| [669] | WANG X. | Cultural Perspectives On Basketball Artificial Intelligence Assistant Referee Mode: A Research Approach With Associative Memory Neural Network | Exclude: Irrelevant to the research topic |
| [670] | WANG X, TüRETKEN E, FLEURET F, et al. | Tracking Interacting Objects Optimally Using Integer Programming | Exclude: Irrelevant to the research topic |
| [671] | WANG Y. | Kinect Body Sensor Technology-Based Quantitative Assessment Method For Basketball Teaching | Exclude: Irrelevant to the research topic |
| [672] | WANG Y, LIU W, LIU X. | Explainable AI Techniques With Application To NBA Gameplay Prediction | Include |
| [673] | WANG Y, SUN M, LIU L. | Basketball Shooting Angle Calculation And Analysis By Deeply-Learned Vision Model | Exclude: Irrelevant to the research topic |
| [674] | WANG Y, YAMASHITA H. | Line-Up Optimization Model Of Basketball Players And The Simulation Evaluation | Exclude: Irrelevant to the research topic |
| [675] | WANG Y J, HUANG G. | Target Tracking Algorithm Of Basketball Video Based On Improved Grey Neural Network | Exclude: Irrelevant to the research topic |
| [676] | WANG Y-S, LI Q-Y. | Prediction Of Basketball Competition Scores Based On BP Neural Network Algorithm | Exclude: Publication outside the 2019-2024 period |
| [677] | WANG Z. | Parameter Optimization And State Evaluation Of Basketball Teaching Based On BPNN | Exclude: Irrelevant to the research topic |
| [678] | WANG Z, LIU Y. | A Method Of Using Data Mining And Edge Computing To Calculate The Standing Efficiency Of Basketball Games | Exclude: Irrelevant to the research topic |
| [679] | WANG Z, LIU Y, ZHANG S. | Preparation Method Of High Resilience Nonslip Basketball Sole Composite Material | Exclude: Irrelevant to the research topic |
| [680] | WANLESS L, NARAINE M L. | Analogous Forecasting For Predicting Sport Innovation Diffusion: From Business Analytics To Natural Language Processing | Exclude: Irrelevant to the research topic |
| [681] | WANLESS L, SEIFRIED C, BOUCHET A, et al. | The Diffusion Of Natural Language Processing In Professional Sport | Exclude: Irrelevant to the research topic |
| [682] | WATCHARAPINCHAI N, ARAMVITH S, SIDDHICHAI S, et al. | A Discriminant Approach To Sports Video Classification | Exclude: Irrelevant to the research topic |
| [683] | WEI J. | Study And Application Of Computer Information Big Data In Basketball Vision System Using High-Definition Camera Motion Data Capture | Exclude: Irrelevant to the research topic |
| [684] | WEI W, QIN Z, YAN B, et al. | Application Effect Of Motion Capture Technology In Basketball Resistance Training And Shooting Hit Rate In Immersive Virtual Reality Environment | Exclude: Irrelevant to the research topic |
| [685] | WEI X, SHA L, LUCEY P, et al. | Predicting Ball Ownership In Basketball From A Monocular View Using Only Player Trajectories | Exclude: Irrelevant to the research topic |
| [686] | WENLI L. | Prospects Of Applying Artificial Intelligence To Determine Students' Mental Health Status In School Education | Exclude: Irrelevant to the research topic |
| [687] | WU F, WANG Q, BIAN J, et al. | A Survey On Video Action Recognition In Sports: Datasets, Methods And Applications | Exclude: Irrelevant to the research topic |
| [688] | WU H, WANG L. | Analysis Of Lower Limb High-Risk Injury Factors Of Patellar Tendon Enthesis Of Basketball Players Based On Deep Learning And Big Data | Exclude: Irrelevant to the research topic |
| [689] | WU L. | The Participating Team's Technical Analysis Of Women's Basketball In The 30th Olympic Games Based On Neural Network | Exclude: Irrelevant to the research topic |
| [690] | WU L, LI Z, XIANG Y, et al. | Latent Label Mining For Group Activity Recognition In Basketball Videos | Exclude: Irrelevant to the research topic |
| [691] | WU L, YANG Z, HE J, et al. | Ontology-Based Global And Collective Motion Patterns For Event Classification In Basketball Videos | Exclude: Irrelevant to the research topic |
| [692] | WU L, YANG Z, WANG Q, et al. | Fusing Motion Patterns And Key Visual Information For Semantic Event Recognition In Basketball Videos | Exclude: Irrelevant to the research topic |
| [693] | WU L, ZHAO K, JIAN M, et al. | Video Key Frame Detection Method By Cascaded Manual Feature And Depth Feature | Exclude: Irrelevant to the research topic |
| [694] | WU Q. | Combination Of Adaptive Object Model For Basketball Tracking | Exclude: Irrelevant to the research topic |
| [695] | XI S, SONG Z, ZHU X, et al. | Design Of Gesture Recognition Mobile Car Based On Yolov5 | Exclude: Irrelevant to the research topic |
| [696] | XIA L, ZHOU H, CHEN J, et al. | Human Body Electrode Enabled Direct Current Triboelectric Nanogenerator For Self-Powered Wireless Human Motion And Environment Monitoring | Exclude: Irrelevant to the research topic |
| [697] | XIA L-M, WANG Q, WU L-S. | Vision-Based Behavior Prediction Of Ball Carrier In Basketball Matches | Exclude: Irrelevant to the research topic |
| [698] | XIA T. | Embedded Basketball Motion Detection Video Target Tracking Algorithm Based On Deep Learning | Exclude: Irrelevant to the research topic |
| [699] | XIAO J, TIAN W, DING L. | Basketball Action Recognition Method Of Deep Neural Network Based On Dynamic Residual Attention Mechanism | Exclude: Irrelevant to the research topic |
| [700] | XIAOHONG G, YU W. | Analysis Of Basketball Training Model Optimization Based On Artificial Intelligence And Computer Aided Model | Exclude: Irrelevant to the research topic |
| [701] | XIN W. | Application Of Intelligent Trajectory Analysis Based On New Spectral Imaging Technology In Basketball Match Motion Recognition | Exclude: Irrelevant to the research topic |
| [702] | XING D, WANG Y, SUN P, et al. | A CNN-LSTM-Att Hybrid Model For Classification And Evaluation Of Growth Status Under Drought And Heat Stress In Chinese Fir (Cunninghamia Lanceolata) | Exclude: Irrelevant to the research topic |
| [703] | XING J, AI H, LIU L, et al. | Multiple Player Tracking In Sports Video: A Dual-Mode Two-Way Bayesian Inference Approach With Progressive Observation Modeling | Exclude: Irrelevant to the research topic |
| [704] | XING J, ZHENG X, ZHANG S, et al. | Computer Simulation Evaluation Model Of Basketball Match Based On Fuzzy Neural Network | Exclude: Irrelevant to the research topic |
| [705] | XU C, ZHANG Y-F, ZHU G, et al. | Using Webcast Text For Semantic Event Detection In Broadcast Sports Video | Exclude: Irrelevant to the research topic |
| [706] | XU G, SHEN G, LIANG X, et al. | Recognition Of Basketball Tactics Based On Vision Transformer And Track Filter | Exclude: Irrelevant to the research topic |
| [707] | XU H, YAN R, KOLIVAND H, et al. | Research On Sports Action Recognition System Based On Cluster Regression And Improved ISA Deep Network | Exclude: Irrelevant to the research topic |
| [708] | XU J. | Recognition Method Of Basketball Players’ Shooting Action Based On Graph Convolution Neural Network | Exclude: Irrelevant to the research topic |
| [709] | XU J, ZHOU Y, ZHANG S. | Team Performance Indicators Explain Outcome Of Women’s 3x3 Basketball At Tokyo 2020 Olympics | Exclude: Irrelevant to the research topic |
| [710] | XU Q, JIA Q. | The Cultivation And Training Effect Of The Subconscious Mind In Physical Education And Training By Intelligent Internet Of Things Network Computing | Exclude: Irrelevant to the research topic |
| [711] | XU T, TANG L. | Adoption Of Machine Learning Algorithm-Based Intelligent Basketball Training Robot In Athlete Injury Prevention | Exclude: Irrelevant to the research topic |
| [712] | XUAN X, XU H. | Complex Sports Target Tracking With Machine Learning: Take Basketball As An Example | Exclude: Irrelevant to the research topic |
| [713] | XUE L. | Application Of Artificial Intelligence In Digital Games Based On Mathematical Statistics | Exclude: Irrelevant to the research topic |
| [714] | YAHYASOLTANI N, ANNAPUREDDY P, FARAZI M. | Learning Performance Efficiency Of College Basketball Players Using TVAE | Exclude: Irrelevant to the research topic |
| [715] | YAN D. | Design And Application Of SPOC Hybrid Teaching For College Basketball Teaching Based On Artificial Intelligence Technology | Exclude: Irrelevant to the research topic |
| [716] | YAN W, JIANG X, LIU P. | A Review Of Basketball Shooting Analysis Based On Artificial Intelligence | Exclude: Irrelevant to the research topic |
| [717] | YAN Y, ZHUANG N, NI B, et al. | Fine-Grained Video Captioning Via Graph-Based Multi-Granularity Interaction Learning | Exclude: Irrelevant to the research topic |
| [718] | YAN Z, YU Y, SHABAZ M. | Optimization Research On Deep Learning And Temporal Segmentation Algorithm Of Video Shot In Basketball Games | Exclude: Irrelevant to the research topic |
| [719] | YANAI C, SOLOMON A, KATZ G, et al. | Q-Ball: Modeling Basketball Games Using Deep Reinforcement Learning | Exclude: Irrelevant to the research topic |
| [720] | YANG G, XU X. | Sequence Video And Artificial Intelligence Assisted Basketball Injury Risk Early Warning Method | Exclude: Irrelevant to the research topic |
| [721] | YANG K. | Research On The Basketball Goal Recognition Method Based On Improved Mobilenet | Exclude: Irrelevant to the research topic |
| [722] | YANG Q, SHAO J, ZUO H. | Automatic Analysis Of Basketball Shooting Based On Machine Learning | Exclude: Irrelevant to the research topic |
| [723] | YANG T, JIANG C, LI P. | Video Analysis And System Construction Of Basketball Game By Lightweight Deep Learning Under The Internet Of Things | Exclude: Irrelevant to the research topic |
| [724] | YANG X, SUMETTIKOON P. | An Advanced Mixed Methodology Model For Teaching Of Physical Education In The Post Covid-19 Era: A Case Study On Junior Middle School Basketball Class | Exclude: Irrelevant to the research topic |
| [725] | YANG Y, PAUL A, CHEUNG S K S, et al. | Research On Basketball Sports Neural Network Model Based On Nonlinear Classification | Exclude: Irrelevant to the research topic |
| [726] | YANG Y, XU M, WU W, et al. | 3D Multiview Basketball Players Detection And Localization Based On Probabilistic Occupancy | Exclude: Irrelevant to the research topic |
| [727] | YANG Z. | Research On Basketball Players' Training Strategy Based On Artificial Intelligence Technology | Exclude: Irrelevant to the research topic |
| [728] | YANG Z. | Prediction Method Of Basketball Players’ Shooting Rate Based On Quantum Search Algorithm | Exclude: Irrelevant to the research topic |
| [729] | YAO A. | Comparing Neural And Regression Models To Predict NBA Team Records | Include |
| [730] | YAO P. | Real-Time Analysis Of Basketball Sports Data Based On Deep Learning | Exclude: Irrelevant to the research topic |
| [731] | YAO W, LUO C, AI F, et al. | Risk Factors For Nonspecific Low-Back Pain In Chinese Adolescents: A Case-Control Study | Exclude: Irrelevant to the research topic |
| [732] | YEH R A, SCHWING A G, HUANG J, et al. | Diverse Generation For Multi-Agent Sports Games | Exclude: Irrelevant to the research topic |
| [733] | YIBING M, HONGYU G, YUQI S, et al. | Real-Time Prediction Algorithm And Simulation Of Sports Results Based On Internet Of Things And Machine Learning | Exclude: Focus on non-professional basketball leagues |
| [734] | YIN L, HE R. | Target State Recognition Of Basketball Players Based On Video Image Detection And FPGA | Exclude: Irrelevant to the research topic |
| [735] | YING X, ZHA H. | Camera Calibration From A Circle And A Coplanar Point At Infinity With Applications To Sports Scenes Analyses | Exclude: Irrelevant to the research topic |
| [736] | YOON J H, PARK J S, SUNG M Y. | Vision-Based Bare-Hand Gesture Interface For Interactive Augmented Reality Applications | Exclude: Irrelevant to the research topic |
| [737] | YOON Y, HWANG H, CHOI Y, et al. | Analyzing Basketball Movements And Pass Relationship Using Realtime Object Tracking Techniques Based On Deep Learning | Exclude: Irrelevant to the research topic |
| [738] | YOUNG M E. | Nonlinear Judgment Analysis: Comparing Policy Use By Those Who Draft And Those Who Coach | Exclude: Irrelevant to the research topic |
| [739] | YU A, CHUNG S S, SOC I C. | Framework For Analysis And Prediction Of NBA Basketball Plays: On-Ball Screens | Exclude: Irrelevant to the research topic |
| [740] | YU S, LIU J. | Automatic Detection Of Image Features In Basketball Shooting Teaching Based On Artificial Intelligence | Exclude: Irrelevant to the research topic |
| [741] | YU X, LYU X, XIANG L, et al. | Reading Two Digital Video Clocks For Broadcast Basketball Videos | Exclude: Irrelevant to the research topic |
| [742] | YU Y Y, WU P P, MENGERSEN K, et al. | Classifying Ball Trajectories In Invasion Sports Using Dynamic Time Warping: A Basketball Case Study | Exclude: Irrelevant to the research topic |
| [743] | YU Y Y, WU P P-Y, MENGERSEN K, et al. | Classifying Ball Trajectories In Invasion Sports Using Dynamic Time Warping: A Basketball Case Study | Exclude: Irrelevant to the research topic |
| [744] | YUAN B, KAMRUZZAMAN M M, SHAN S. | Application Of Motion Sensor Based On Neural Network In Basketball Technology And Physical Fitness Evaluation System | Exclude: Irrelevant to the research topic |
| [745] | YUAN Y, LU Z, YANG Z, et al. | Key Frame Extraction Based On Global Motion Statistics For Team-Sport Videos | Exclude: Irrelevant to the research topic |
| [746] | YUANZHEN N, SEONGNO L. | A Comparative Study Of Machine Learning Models For NCAA Men's Basketball Tournament Games Outcome Prediction | Exclude: Focus on non-professional basketball leagues |
| [747] | YUE Q. | Dynamic Database Design Of Sports Quality Based On Genetic Data Algorithm And Artificial Intelligence | Exclude: Irrelevant to the research topic |
| [748] | YUE Q, WEI C. | Innovation Of Human Body Positioning System And Basketball Training System | Exclude: Irrelevant to the research topic |
| [749] | YUE T, ZOU Y. | Online Teaching System Of Sports Training Based On Mobile Multimedia Communication Platform | Exclude: Irrelevant to the research topic |
| [750] | YUE Y, LUCEY P, CARR P, et al. | Learning Fine-Grained Spatial Models For Dynamic Sports Play Prediction | Exclude: Irrelevant to the research topic |
| [751] | ZEMKOVA E. | Agility Index As A Measurement Tool Based On Stimuli Number And Traveling Distances | Exclude: Irrelevant to the research topic |
| [752] | ZEYU L. | Application Of Optical Imaging Equipment Based On Deep Neural Network In Basketball Training Game Simulation | Exclude: Irrelevant to the research topic |
| [753] | ZHAN E, TSENG A, YUE Y, et al. | Learning Calibratable Policies Using Programmatic Style-Consistency | Exclude: Irrelevant to the research topic |
| [754] | ZHANG A, CHEN K. | Simulation Of Image Feature Extraction Based On Optical Sensors In Basketball Target Recognition System | Exclude: Irrelevant to the research topic |
| [755] | ZHANG B, HUANG H, PAN X, et al. | Context-Aware Entity Morph Decoding | Exclude: Irrelevant to the research topic |
| [756] | ZHANG B, WANG T. | Visual Image Recognition Of Basketball Turning And Dribbling Based On Feature Extraction | Exclude: Irrelevant to the research topic |
| [757] | ZHANG D C, YING R, LAUW H W, et al. | Hyperbolic Graph Topic Modeling Network With Continuously Updated Topic Tree | Exclude: Irrelevant to the research topic |
| [758] | ZHANG F, HUANG Y, REN W. | Basketball Sports Injury Prediction Model Based On The Grey Theory Neural Network | Exclude: Irrelevant to the research topic |
| [759] | ZHANG F, JIANG Y. | Basketball Action Data Processing Method Based On Mode Symmetric Algorithm | Exclude: Irrelevant to the research topic |
| [760] | ZHANG J, CAO Y, QIAO M, et al. | Human Motion Monitoring In Sports Using Wearable Graphene-Coated Fiber Sensors | Exclude: Irrelevant to the research topic |
| [761] | ZHANG J, MAO H. | WKNN Indoor Positioning Method Based On Spatial Feature Partition And Basketball Motion Capture | Exclude: Irrelevant to the research topic |
| [762] | ZHANG J, SHI X. | Design Of Execution System Based On Artificial Intelligence Technology | Exclude: Irrelevant to the research topic |
| [763] | ZHANG J, TAO D. | Research On Deep Reinforcement Learning Basketball Robot Shooting Skills Improvement Based On End To End Architecture And Multi-Modal Perception | Exclude: Irrelevant to the research topic |
| [764] | ZHANG K, LIU T, LIU Z, et al. | Multimodal Human-Computer Interactive Technology For Emotion Regulation | Exclude: Irrelevant to the research topic |
| [765] | ZHANG L. | Behaviour Detection And Recognition Of College Basketball Players Based On Multimodal Sequence Matching And Deep Neural Networks | Exclude: Irrelevant to the research topic |
| [766] | ZHANG N, HAN Y, CRESPO R G, et al. | Physical Education Teaching For Saving Energy In Basketball Sports Athletics Using Hidden Markov And Motion Model | Exclude: Irrelevant to the research topic |
| [767] | ZHANG N, IZQUIERDO E. | A Four-Point Camera Calibration Method For Sport Videos | Exclude: Irrelevant to the research topic |
| [768] | ZHANG P, LUO J. | Player Detection Method Based On Scale Attention And Scale Equalization Algorithm | Exclude: Irrelevant to the research topic |
| [769] | ZHANG Q. | Prediction Based On Basketball Competition Video Athlete Behaviors And On-Line RBF Neural Network Application | Exclude: Irrelevant to the research topic |
| [770] | ZHANG Q. | Evaluation And Prediction Of Sports Health Literacy Of College Students Based On Artificial Neural Network | Exclude: Irrelevant to the research topic |
| [771] | ZHANG Q. | Lightweight Classification Method Of Human Action In Long Video Of Basketball Competition | Exclude: Irrelevant to the research topic |
| [772] | ZHANG Q, HUANG Y. | Analysis Of Tianjin Basketball Comprehensive Based On Grey Incidence Analysis From 2012-2013 CBA | Exclude: Irrelevant to the research topic |
| [773] | ZHANG Q, ZHAO Y, QIAN Z, et al. | A Wearable Three-Axis Force Sensor Based On Deep Learning Technology For Plantar Measurement | Exclude: Irrelevant to the research topic |
| [774] | ZHANG R. | Impact Analysis Of Basketball Exercise Strength Based On Machine Learning In The Mental Health Of College Students | Exclude: Irrelevant to the research topic |
| [775] | ZHANG R, WU L, YANG Y, et al. | Multi-Camera Multi-Player Tracking With Deep Player Identification In Sports Video | Exclude: Irrelevant to the research topic |
| [776] | ZHANG S, ZHAO G, LIN P, et al. | Deep Reinforcement Learning For A Humanoid Robot Basketball Player | Exclude: Irrelevant to the research topic |
| [777] | ZHANG W, JIAO L, LI Y, et al. | Laplacian Feature Pyramid Network For Object Detection In VHR Optical Remote Sensing Images | Exclude: Irrelevant to the research topic |
| [778] | ZHANG W, JIAO L, LIU F, et al. | Lhnet: Laplacian Convolutional Block For Remote Sensing Image Scene Classification | Exclude: Irrelevant to the research topic |
| [779] | ZHANG W, JIAO L, LIU X, et al. | Multi-Scale Feature Fusion Network For Object Detection In VHR Optical Remote Sensing Images | Exclude: Irrelevant to the research topic |
| [780] | ZHANG W, ZHANG X. | An Improved Integer Local Search For Complex Scheduling Problems | Exclude: Irrelevant to the research topic |
| [781] | ZHANG X, DUAN H, ZHANG M, et al. | Wrist MEMS Sensor For Movements Recognition In Ball Games | Exclude: Irrelevant to the research topic |
| [782] | ZHANG X, OGASAWARA I, KONDA S, et al. | Absorption Function Loss Due To The History Of Previous Ankle Sprain Explored By Unsupervised Machine Learning | Exclude: Irrelevant to the research topic |
| [783] | ZHANG Y, WEI W. | Sports Training Correction Based On 3D Virtual Image Model | Exclude: Irrelevant to the research topic |
| [784] | ZHANG Y, WEI W. | Research On The Application Of Behavioral Image Feature Capture In Basketball Game Video | Exclude: Irrelevant to the research topic |
| [785] | ZHANG Y, ZHAO G. | Conservative Treatment And Rehabilitation Training For Rectus Femoris Tear In Basketball Training Based On Computer Vision | Exclude: Irrelevant to the research topic |
| [786] | ZHANG Z, ZHANG L, WANG C, et al. | Bayesian Network Learning For Winning Structure And Losing Structure In Basketball Games | Exclude: Irrelevant to the research topic |
| [787] | ZHAO B, LIU J. | Research On The Application Of Light Detection Sensors Based On Deep Learning In Basketball Training Monitoring | Exclude: Irrelevant to the research topic |
| [788] | ZHAO D. | Injuries In College Basketball Sports Based On Machine Learning From The Perspective Of The Integration Of Sports And Medicine | Exclude: Irrelevant to the research topic |
| [789] | ZHAO K, HOHMANN A, CHANG Y, et al. | Physiological, Anthropometric, And Motor Characteristics Of Elite Chinese Youth Athletes From Six Different Sports | Exclude: Irrelevant to the research topic |
| [790] | ZHAO L, CHEN W. | Detection And Recognition Of Human Body Posture In Motion Based On Sensor Technology | Exclude: Irrelevant to the research topic |
| [791] | ZHAO Q. | Modeling And Analysis Method Of National Fitness Big Data For Basketball Projects Based On A Multivariate Statistical Model | Exclude: Irrelevant to the research topic |
| [792] | ZHAO Y. | Deep Learning Of 3D High-Precision Model Digital Engraving Of Next-Generation Games Based On Artificial Intelligence | Exclude: Irrelevant to the research topic |
| [793] | ZHAO Y, LIU J, LIAN C, et al. | A Single Smart Ring For Monitoring 20 Kinds Of Multi-Intensity Daily Activities--From Kitchen Work To Fierce Exercises | Exclude: Irrelevant to the research topic |
| [794] | ZHAO Y, WANG X, LI J, et al. | Using Iot Smart Basketball And Wristband Motion Data To Quantitatively Evaluate Action Indicators For Basketball Shooting | Exclude: Irrelevant to the research topic |
| [795] | ZHAO Y, YANG R, CHEVALIER G, et al. | Applying Deep Bidirectional LSTM And Mixture Density Network For Basketball Trajectory Prediction | Exclude: Irrelevant to the research topic |
| [796] | ZHAO Y, ZHANG X, YANG M, et al. | Shooting Prediction Based On Vision Sensors And Trajectory Learning | Exclude: Irrelevant to the research topic |
| [797] | ZHENG S, YUE Y, LUCEY P. | Generating Long-Term Trajectories Using Deep Hierarchical Networks | Exclude: Irrelevant to the research topic |
| [798] | ZHENG W, YUAN M. | A Person Re-Identification Approach Focusing On The Occlusion Problem And Ranking Optimization | Exclude: Irrelevant to the research topic |
| [799] | ZHENG X. | NBA Winner Prediction: A Hybrid Framework Incorporating Internal And External Factors | Include |
| [800] | ZHENG Y. | Video Analysis And 3d Detection Simulation Of Jump Shot Precision For Basketball Players | Exclude: Irrelevant to the research topic |
| [801] | ZHENG Z, MA H, YAN W, et al. | Training Data Selection And Optimal Sensor Placement For Deep-Learning-Based Sparse Inertial Sensor Human Posture Reconstruction | Exclude: Irrelevant to the research topic |
| [802] | ZHI J, SUN Z, ZHANG R, et al. | Badminton Video Action Recognition Based On Time Network | Exclude: Irrelevant to the research topic |
| [803] | ZHONG S. | Application Of Artificial Intelligence And Big Data Technology In Basketball Sports Training | Exclude: Irrelevant to the research topic |
| [804] | ZHOU L, ZHANG C, WANG M. | Emotion Recognition Algorithm Of Basketball Players Based On Deep Learning | Exclude: Irrelevant to the research topic |
| [805] | ZHOU W, HE X, ZHONG W, et al. | Efficient Learning Of Quadratic Variance Function Directed Acyclic Graphs Via Topological Layers | Exclude: Irrelevant to the research topic |
| [806] | ZHOU W S, DAO S, KUO C C J. | On-Line Knowledge- And Rule-Based Video Classification System For Video Indexing And Dissemination | Exclude: Irrelevant to the research topic |
| [807] | ZHOU W S, KUO C C J. | Knowledge-Based Inference Engine For On-Line Video Dissemination | Exclude: Irrelevant to the research topic |
| [808] | ZHOU Y, WANG R, WANG Y, et al. | A Swarm Intelligence Assisted Iot-Based Activity Recognition System For Basketball Rookies | Exclude: Irrelevant to the research topic |
| [809] | ZHU N, DAI Q. | Basketball Data Analysis Based On Spark Framework And K-Means Algorithm | Exclude: Irrelevant to the research topic |
| [810] | ZIMMERMANN A. | Exploring Chance In NCAA Basketball | Exclude: Irrelevant to the research topic |
| [811] | ZIYI Z, TAKEDA K, FUJII K. | Cooperative Play Classification In Team Sports Via Semi-Supervised Learning | Exclude: Irrelevant to the research topic |
| [812] | ZOU W, JIN Z. | Feature Extraction Of Basketball Shooting Based On Apriori Algorithm | Exclude: Irrelevant to the research topic |
| [813] | ZUCCOLOTTO P, SANDRI M, MANISERA M. | Spatial Performance Analysis In Basketball With CART, Random Forest And Extremely Randomized Trees | Exclude: Irrelevant to the research topic |
| [814] | ZUO K, SU X. | Three-Dimensional Action Recognition For Basketball Teaching Coupled With Deep Neural Network | Exclude: Irrelevant to the research topic |
| [815] | 고기준, 안병철. | The Effect Of Calorie Restriction And Aerobic Exercise On Serum Lipids And Apolipoprotein Metabolism In Obses Children | Exclude: Irrelevant to the research topic |
| [816] | 박태곤. | Effects Of Aerobic Training Plus Diet On Blood Lipids And Apolipoproteins In Obese Children | Exclude: Irrelevant to the research topic |
| [817] | Cai W, Yu D, Wu Z, Du X, Zhou T. | A Hybrid Ensemble Learning Framework For Basketball Outcomes Prediction | Include |
| [818] | Giasemidis G. | Descriptive And Predictive Analysis Of Euroleague Basketball Games And The Wisdom Of Basketball Crowds | Include |
| [819] | Huang M-L, Lin Y-J. | Regression Tree Model For Predicting Game Scores For The Golden State Warriors In The National Basketball Association | Include |
| [820] | Khanmohammadi R, Saba-Sadiya S, Esfandiarpour S, Alhanai T, Ghassemi MM. | Mambanet: A Hybrid Neural Network For Predicting The NBA Playoffs | Include |
| [821] | Teno, González Dos Santos, Wang C , Carlsson N ,et al. | Predicting Season Outcomes For The NBA | Include |
| [822] | Wang J. | Predictive Analysis Of NBA Game Outcomes Through Machine Learning | Include |
| [823] | Song K, Gao Y, Shi J. | Making Real-Time Predictions For NBA Basketball Games By Combining The Historical Data And Bookmaker’S Betting Line | Include |
| [824] | Zhao K, Du C, Tan G. | Enhancing Basketball Game Outcome Prediction Through Fused Graph Convolutional Networks And Random Forest Algorithm | Include |
